# Exploring the antimicrobial activity of pantothenamides against uropathogenic *Escherichia coli*

**DOI:** 10.1128/spectrum.03069-24

**Published:** 2025-06-23

**Authors:** Alicia A. DeColli, David J. Meyers, Arina Ranjit, James Paule, Erika Serrano-Diaz, Kimberly M. Bockley, Laura M. Ensign, Rana Rais, Caren L. Freel Meyers

**Affiliations:** 1 Department of Pharmacology and Molecular Sciences, The Johns Hopkins University School of Medicine 224079, Baltimore, Maryland, USA; 2 Department of Neurology, Johns Hopkins University School of Medicine 1500, Baltimore, Maryland, USA; 3 Johns Hopkins Drug Discovery, Johns Hopkins University School of Medicine 466098, Baltimore, Maryland, USA; 4 Center for Nanomedicine at the Wilmer Eye Institute, Department of Ophthalmology, Johns Hopkins University School of Medicine 540150, Baltimore, Maryland, USA; 5 Department of Chemical and Biomolecular Engineering, Johns Hopkins University 198421, Baltimore, Maryland, USA; 6 Department of Biomedical Engineering, Johns Hopkins University 171259, Baltimore, Maryland, USA; 7 Department of Gynecology and Obstetrics, Johns Hopkins University School of Medicine 519921, Baltimore, Maryland, USA; 8 Division of Infectious Diseases, Department of Oncology, Johns Hopkins University School of Medicine 229385, Baltimore, Maryland, USA; University of Kentucky, Lexington, Kentucky, USA

**Keywords:** CoA biosynthesis, pantothenamides, N5-Pan, uropathogenic *E. coli*, urinary tract infection, DXP synthase, drug discovery, antimicrobial activity

## Abstract

Metabolic processes required for bacterial pathogens to adapt in the host are potential targets for the development of much-needed new antimicrobial agents. The bacterial central metabolic enzyme 1-deoxy-d-xylulose 5-phosphate synthase (DXPS) is required for the synthesis of essential isoprenoids, thiamin diphosphate (ThDP), and pyridoxal phosphate (PLP) and is believed to function in bacterial adaptations requiring these metabolites. DXPS inhibition impairs a PLP-dependent adaptation of Uropathogenic *Escherichia coli* (UPEC) to d-serine, a bacteriostatic host metabolite in urine that inhibits pantothenate production in Coenzyme A (CoA) biosynthesis. CoA is required for a functioning tricarboxylic acid (TCA cycle, which is critical for UPEC survival in the urinary tract. Accordingly, inhibition of DXPS also sensitizes UPEC to *N*-pentylpantothenamide (N5-Pan, **1**), an inhibitor of CoA synthesis. However, **1** is enzymatically hydrolyzed by pantetheinases, presenting challenges for its use in *in vivo* studies. We sought to identify a pantothenamide inhibitor candidate for studies to explore the *in vivo* efficacy of inhibitor combinations targeting DXPS and CoA syntheses. Here, we describe studies that highlight a truncated analog of **1**, pantetheinase-resistant N5-α-Pan (**6**), as a promising candidate for *in vivo* studies. Analog **6** exhibited comparable antimicrobial activity with **1** against UPEC grown in nutrient-limited culture conditions, including urine, and displayed enhanced activity in the presence of a DXPS inhibitor. In contrast to **1**, analog **6** was stable in the presence of mouse plasma and liver enzymes, making it suitable for studies to investigate an inhibitor combination targeting DXPS and CoA synthesis in UPEC by the *in vivo* ascending UTI assay.

**IMPORTANCE:**

New approaches are needed to control the emergence of drug resistance in bacterial pathogens that cause life-threatening infections. Targeting DXPS-dependent synthesis of vitamins is a promising approach to prevent pathogen metabolic adaptation. Metabolic processes requiring DXPS-dependent synthesis of pyridoxal phosphate (PLP) or thiamin diphosphate (ThDP) can become particularly vulnerable in a specific pathogen and/or host environment, under conditions of DXPS inhibition. We previously observed that UPEC grown in urine is particularly sensitive to an inhibitor combination targeting DXPS and CoA synthesis; however, the CoA inhibitor is readily hydrolyzed by a host pantetheinase. This study is significant, as it identifies a pantothenamide inhibitor of CoA synthesis, N5-α-Pan (**6**), that is stable to mouse plasma and liver enzymes and whose activity is enhanced in the presence of a DXPS inhibitor. Thus, **6** is suitable for studies to explore how a pathogen can become sensitized *in vivo* under conditions of DXPS inhibition.

## INTRODUCTION

**Fig 1:**
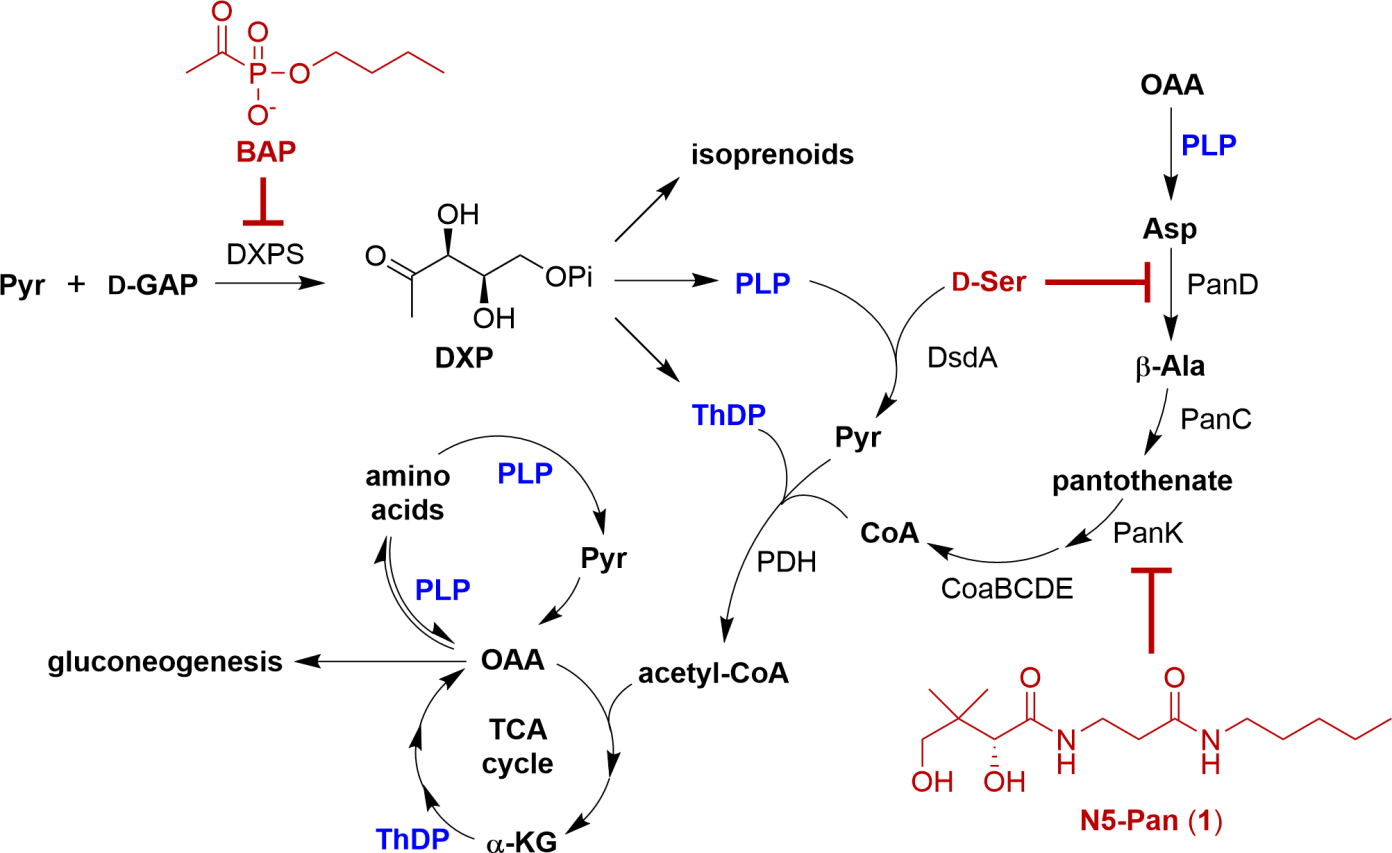
Formation of DXP from pyruvate and d-GAP. DXP is a branchpoint metabolite feeding into the synthesis of ThDP, PLP, and isoprenoids. PLP and ThDP are critical for UPEC adaptation to urine where amino acids are a main carbon source. UPEC also detoxifies d-Ser in a PLP-dependent manner. Processes requiring PLP or ThDP are highlighted in blue. Inhibition of DXPS, PanD, and PanK by BAP, d-serine, and N5-Pan, respectively, is highlighted in red. *Abbreviations:* acetyl-CoA (acetyl coenzyme A), ꞵ-Ala (ꞵ-alanine), Asp (aspartate), CoA (coenzyme A), , CoaB (4′-phosphopantothenoylcysteine synthetase), CoaC (4′-phosphopantothenoylcysteine decarboxylase), CoaD (4′-phosphopantetheine adenylyltransferase), CoaE (dephospho-CoA kinase), DsdA (d-serine deaminase), DXP (1-deoxy-d-xylulose 5-phosphate), DXPS (1-deoxy-d-xylulose 5-phosphate synthase), d-GAP (d-glyceraldehyde 3-phosphate), α-KG (α-ketoglutarate), OAA (oxaloacetate), PanC (pantothenate synthase), PanD (aspartate decarboxylase), PLP (pyridoxal 5-phosphate), PanK (pantothenate kinase), PDH (pyruvate dehydrogenase), Pyr (pyruvate), d-Ser (d-serine), and ThDP (thiamin diphosphate).

Targeting processes in pathogenic bacteria that are required for adaptation to the host environment is a promising strategy to combat and control the emergence of antimicrobial resistance (1, 2). Pathogenic bacteria are remarkably adept at remodeling their metabolism in response to changes in nutrient availability, having the capacity to thrive under nutrient limitation during infection and utilize a variety of carbon sources within host tissues, which is critical for pathogenesis (3–5). We have proposed that the central metabolic enzyme 1-deoxy-d-xylulose 5-phosphate synthase (DXPS) plays key roles in bacterial adaptation, and DXPS inhibition has the potential to create metabolic vulnerabilities in an infection-specific manner, which can be exploited in the development of narrow-spectrum combination therapies (6–9). DXPS catalyzes the ThDP-dependent decarboxylation of pyruvate and subsequent carboligation with d-glyceraldehyde 3-phosphate (d-GAP) to form DXP (Fig. 1). DXP is an essential metabolite found in most bacteria, including many bacterial pathogens, but not in humans (10–16). In its canonical role, DXP functions at a branch point at the heart of bacterial central metabolism, serving as a precursor in the biosynthesis of isoprenoids, ThDP, and PLP (17–20). DXPS-dependent synthesis of vitamins and isoprenoids is thought to be critical for the adaptation of pathogens moving between different nutrient niches.

The mechanism of DXPS is distinct from human ThDP-dependent enzymes (9, 21–26) and has inspired the development of selective inhibitors, such as butylacetylphosphonate (BAP, Fig. 1), to be used as functional probes of DXPS and potential starting points for antimicrobial development (6, 8, 27–33). BAP was recently employed in the first studies to interrogate DXPS-dependent metabolic adaptations of a clinically relevant bacterial pathogen, Uropathogenic *E. coli* (UPEC). UPEC causes 65%–75% of urinary tract infections (UTI), which are among the most common infections worldwide (34). Given the rise in mortality from UTI linked to the increasing prevalence of antibiotic-resistant UPEC, there is a renewed focus on developing new antimicrobial approaches against this pathogen (35). Furthermore, UTI represents an ideal infection model to investigate DXPS-dependent adaptations as antimicrobial targets. UPEC has an exceptional ability to remodel its central metabolism in response to fluctuations in nutrient availability, as it moves from the nutrient-rich niche of the gut, where it is non-pathogenic, to the nutrient-limited environment of the urinary tract, where it becomes pathogenic (3, 4, 36). In the urinary tract, where amino acids and peptides are the primary carbon source, UPEC relies heavily on ThDP and PLP to support TCA cycle function and gluconeogenesis from amino acids (36–39). In addition, UPEC is resistant to the bacteriostatic effects of d-serine (d-Ser), a host metabolite present at high concentrations in the urinary tract. We have demonstrated the role of DXPS in the PLP-dependent catabolism of d-Ser by deaminase DsdA in UPEC (Fig. 1) (7). UPEC is sensitive to the growth-inhibitory effects of BAP in urine, and BAP treatment sensitizes UPEC to the toxic effects of d-Ser, underscoring the importance of DXPS in this adaptation (7).

In addition, inhibition of DXPS creates a metabolic vulnerability in UPEC cultured in urine, where d-Ser accumulates as a result of PLP limitation caused by BAP-mediated DXPS inhibition. d-Ser exerts its toxic effects through the inhibition of PanD, an early step in the biosynthesis of CoA, which, together with ThDP and PLP, is required to replenish TCA cycle intermediates for gluconeogenesis in this amino acid-rich environment. Exploiting the increased reliance on ThDP, PLP, and CoA biosynthesis in this environment, we demonstrated that BAP-treated UPEC is particularly vulnerable to the coenzyme A (CoA) biosynthesis inhibitor *N*-pentylpantothenamide (N5-Pan) when grown in urine (**1**, Fig. 1) (7). Thus, inhibition of DXPS impairs the metabolic adaptation of UPEC in the urinary tract and causes a metabolic vulnerability that can be simultaneously targeted through a synergistic inhibitor combination.

A recent study evaluating BAP in an *in vivo* ascending UTI prophylaxis model determined that this DXPS inhibitor partially protects mice against UTI, suggesting that DXPS inhibition diminishes colonization (6). However, the observed potency was weak, presumably a result of the high inoculum density required to establish infection (inoculum effect) and/or the nutrient-rich medium used to generate the inoculum, a condition in which BAP permeability is low (6, 8). Based on our finding that BAP and **1** are synergistic against UPEC grown in urine, we hypothesize that a BAP/CoA synthesis inhibitor combination should be more potent *in vivo* relative to BAP alone. However, the use of **1** *in vivo* is limited by its susceptibility to cleavage by serum pantetheinases, such as vanin-1 protease (40–42). Two approaches can be taken to avoid vanin-1-dependent hydrolysis, including (i) the co-administration of **1** with a vanin-1 inhibitor (42, 43) or (ii) the development of pantetheinase-resistant pantothenamides (44, 45). The pantothenamide inhibitor class has been studied extensively, and many analogs have been developed and evaluated against bacterial and parasitic pathogens, most notably *Klebsiella pneumoniae*, *Staphylococcus aureus,* and the malaria parasite *Plasmodium falciparum* (40, 43, 46–52). However, with the exception of **1**, studies of pantothenamides against *E. coli* are more limited, and **1** is the only analog evaluated against UPEC. The objective of this study was to assess pantothenamide activities against UPEC toward identifying analogs that display higher potency and/or increased hydrolytic stability as potential candidates for *in vivo* studies. N5-α-Pan (**6**), a truncated pantothenamide previously shown to be non-toxic to mammalian cells and stable in the presence of serum pantetheinases (41), and the inverse amide of N5-Pan (**7**), also shown to be stable in the presence of serum pantetheinases (52), emerged from this study as promising candidates. Both analogs exhibit comparable potency to **1** under standard nutrient-limitation conditions and resist enzymatic hydrolysis in mouse liver and plasma. Although **7** lacks potent activity against UPEC grown in urine, **6** displays similar antimicrobial activity to **1**, and its activity is enhanced in the presence of BAP. These findings highlight **6** as a promising candidate for *in vivo* studies to investigate how a metabolic vulnerability caused by DXPS inhibition can be leveraged for the development of antimicrobial strategies to prevent or treat UTI.

## MATERIALS AND METHODS

### General methods and materials

Unless otherwise noted, all reagents were obtained from commercial sources. BAP, **1**, and N5-Pan analogs were synthesized according to previously reported procedures (31, 53). Urine was self-collected from adult males and females (not menstruating) between 24 and 57 years old (recruitment was open to donors 18–65 years old), who had not used anti-infectives or cranberry products for at least 7 days prior to collection. The collection protocol was approved by the Johns Hopkins University Institutional Review Boards under IRB study NA_00085130. Informed, written consent was obtained from all participants prior to collection. Urine was collected from at least 7 participants over 14 days, stored at 4°C upon collection, and then pooled, filter-sterilized, and frozen at −20°C. Urine Batch A included five male and three female donors with an average age of 33 years (range: 24–57). Urine Batch B included four male and five female donors with an average age of 26 years (range: 24–43). Samples from two donors (donations made at least 30 days apart) were included in both Batch A and Batch B. Urine Batch C included four male and three female donors with an average age of 27 years (range: 24–36). Urine Batch D included four male and three female donors with an average age of 27 years (range: 25–35). Solid urine agar plates were prepared by supplementing cooled 1.5% (wt/vol) agar with filter-sterilized urine in a 4:1 ratio. Liquid MOPS-glycerol was prepared by adding 10 × 3-(N-morpholino)propanesulfonic acid (MOPS) Buffer (Teknova) to water to a final 1× concentration, 1.32 mM K_2_HPO_4_, and 0.4% (vol/vol) glycerol and then filter-sterilizing. Unless otherwise noted, all media were supplemented with freshly prepared 20 µM FeSO_4_. OD_600_ analyses were performed on Mettler-Toledo UV5Bio UV/Visible spectrophotometer (Columbus, Ohio, USA). For antimicrobial studies, uropathogenic *E. coli* strain CFT073 (ATCC: BAA-2503) was provided by Kevin Schwartz from the lab of Rodney Welch. All manipulation of UPEC was conducted in a certified biosafety level 2 (BSL-2) laboratory while using BSL-2-associated safety procedures. Antimicrobial data were collected on a Biotek Epoch 2 microplate reader or a Biotek Synergy Neo 2 microplate reader. For metabolic stability studies, losartan potassium (PHR1602), used as an internal standard, was bought from Millipore Sigma (Burlington, MA). Methanol (A456-4) and acetonitrile (A955-4) were purchased from Fisher Chemical (Pittsburgh, PA). Water (9831-03) was purchased from JT Baker (Philipsburg, NJ). Dimethyl sulfoxide (DMSO)(154938-500mL) was purchased from Sigma Aldrich (St. Louis, MO). Mouse plasma (WT) was purchased from Innovative Research (Novi, MI). Mouse liver microsomes (M1000) were purchased from XenoTech LLC (Kansas City, KS).

### Antimicrobial susceptibility assays

All antimicrobial susceptibility assays were conducted as previously described using an aseptic technique with some modification (7). Briefly, 3–5 isolated colonies of CFT073 on LB agar or pooled urine agar were inoculated into 3 mL of liquid MOPS-glycerol medium or pooled urine, respectively, and grown overnight with shaking at 37°C. Saturated cultures were diluted 1:50 in fresh medium and grown to an OD_600_ of 0.4. These exponential phase cultures were then diluted 1:1,000 into fresh medium to yield the experimental inoculum, which was mixed 1:1 with medium containing **1** or N5-Pan analog at twice the desired final concentration in 96-well plates. The final concentration of bacteria in each well was approximately 10^5^ CFU/mL in a final volume of 200 µL. The concentration ranges of compounds evaluated in MOPS-glycerol were: **1** (0–312.5 μM), **2** (0–100 μM), **3** (0–500 μM), **4–5** (0–2,000 μM), **6–7** (0–100 μM), and **8–12** (0-2,000 μM). Analogs **1**, **6,** and **7** were evaluated over a range of 0–4,000 μM in four batches of pooled human urine. The OD600 was monitored for 24 h (MOPS-glycerol) or 16 h (pooled urine) at 37°C with periodic shaking. Consistency between experiments was verified using colony counts of the experimental inoculum by dilution and enumeration on LB agar for 16 h at 37°C. Fractional growth of drug-treated cells was determined at 24 h (MOPS-glycerol) or 6–8 h (pooled urine) relative to the no drug control. Experiments in MOPS-glycerol were performed in biological triplicate. Experiments in pooled human urine were performed in biological duplicate (urine batches A and B) or biological triplicate (urine batches C and D).

### Metabolic stability of pantothenamides 6 and 7

Stability studies were conducted in mouse liver microsomes, mouse liver tissue homogenate, and CD1 mouse plasma as previously described with minor modifications (54–56). Briefly, for Phase I mouse liver microsome stability, reactions were carried out with 200 mM potassium phosphate buffer, pH 7.4, with or without an NADPH-regenerating system (1.3 mM NADPH, 3.3 mM glucose 6-phosphate, 3.3 mM MgCl_2_, 0.4 U/mL glucose-6-phosphate dehydrogenase, and 50 µM sodium citrate). Reactions, in triplicate, were initiated by the addition of liver microsomes to the incubation mixture (compound final concentration was 1 µM; 0.2 mg/mL microsomes) and incubated at 37°C for the desired incubation time. For liver stability, the tissue homogenate was prepared by washing tissues with diluted 10-fold 0.1 M potassium phosphate buffer and homogenized using a probe sonicator over ice. To evaluate the stability of the drugs over time, each of the crude homogenates was aliquoted to 1 mL and then spiked with a final assay concentration of 10 µM of each drug, followed by incubation in an orbital shaker at 37°C for 1 h (in triplicate). For plasma stability, 1 mL of plasma was spiked with 10 µM of each drug, followed by incubation in an orbital shaker at 37°C for 1 h (in triplicate). Samples from each metabolism study were quenched with 3× volume of acetonitrile containing internal standard (losartan: 0.5 µM) at the desired incubation times. Samples from each incubation at predetermined time points were quenched with 3× volumes of acetonitrile containing the internal standard (IS; losartan: 0.5 µM). Samples were vortex-mixed for 30 s and centrifuged at 10,000 *× g* for 10 min at 4°C. Compound disappearance over time was monitored using a Dionex ultra-high-performance liquid chromatography system consisting of an analytical pump and an autosampler coupled with Q Exactive Focus orbitrap mass spectrometer (Thermo Fisher Scientific Inc., Waltham MA). Separation of the analyte from potentially interfering material was achieved at 35°C using Agilent Eclipse Plus column (100 × 2.1 mm i.d.) packed with a 1.8 µm C_18_ stationary phase. The mobile phase used was composed of 0.1% formic acid in water and 0.1% formic acid in acetonitrile with gradient elution. The total run time for each analyte was 5 min.

The mass spectrometer was operated with a HESI ion source in positive ionization mode where compounds were identified in the full scan mode (from m/z 75.00–1125.00) and controlled by the Xcalibur software 4.0.27.13 (Thermo Scientific). Data were acquired using Xcalibur software. Drugs were identified in the full-scan mode (from m/z 75.00 to 1125.00) by comparing t = 0 samples with t = 30 or 60 min samples, and structures were proposed based on the accurate mass information. The percentage of compound remaining for pantothenamides **1**, **6,** and **7** was determined from the analyte/internal standard (IS) area ratios obtained at t = 30 or 60 min relative to the mean ratios obtained at t = 0 min and calculated using equation 1:


$$\%\;Remaining=\left(\frac{\frac{Peak\;area\;of\;Analyte}{Preak\;area\;of\;IS}at\;t=30\;or\;60\;min}{Mean\frac{Peak\;area\;of\;Analyte}{Peak\;area\;of\;IS}at\;t=0\;min}\right)\times 100$$

### Checkerboard analysis

As previously described (7), an inoculum of 10^5^ CFU/mL CFT073 was prepared in pooled human urine Batch B (described above). Cells (100 µL) were added 1:1 to a 96-well plate containing varied combinations of BAP and **1** or N5-Pan analog at 2× the final concentration (100 µL). The OD600 was recorded at 37°C for 16 h with intermittent shaking. Fractional growth was calculated relative to the no drug control at 6 h. Experiments were performed in biological triplicate.

Fractional inhibitory concentration indices (*FIC_I_*) were determined as previously described (7, 8) and calculated using equation 2:


$$FIC_{I}=FIC_{A}+FIC_{B}=\left(\frac{\left[A\right]}{MIC_{A}}\right)+\left(\frac{\left[B\right]}{MIC_{B}}\right)$$

*MIC_A_* and *MIC_B_* are the lowest [*A*] or [*B*], respectively, required to suppress 90% of the bacterial growth. *FIC_A_* was calculated as [*A*] in the presence of *B* for a combination resulting in <10% growth, divided by *MIC_A_. FIC_B_* was calculated as [*B*] in the presence of *A* for a combination resulting in <10% growth, divided by *MIC_B_*. Isobolograms were prepared from the checkerboard analysis by plotting *FIC* for BAP and **1** or **6** over the range of BAP concentrations tested in combination with **1** or **6**. Points falling below the line of additivity indicate potentiation of activity. See Fig. S6 for the visual representation of the method of assembling isobolograms.

## RESULTS

### Pantothenamide selection and synthesis

N5-Pan (**1**) potency, as well as its relationship with the DXPS inhibitor BAP when used in combination with UPEC CFT073, is dependent upon the growth environment (7). The potency of **1** was highest against UPEC grown in MOPS-glycerol with a ≥ 30-fold drop in potency observed in urine. Despite its reduced potency as a single agent in urine, **1** combined with BAP displayed synergy against UPEC in urine but not in MOPS-glycerol. As noted above, the observed potentiation of the activity of **1** by BAP in urine indicates that CoA biosynthesis is particularly vulnerable in DXPS-inhibited UPEC in the context of UTI. However, exploiting this metabolic vulnerability of UPEC *in vivo* will be limited by the susceptibility of **1** to pantetheinase-mediated hydrolysis, which is expected to dramatically lower *in vivo* potency (40–45). This limitation, together with the scarcity of information on pantothenamide activity against uropathogens in relevant growth environments, rationalizes further investigation of pantothenamides against UPEC under conditions relevant to UTI, including analogs designed to be pantetheinase-resistant.

**Fig 2:**
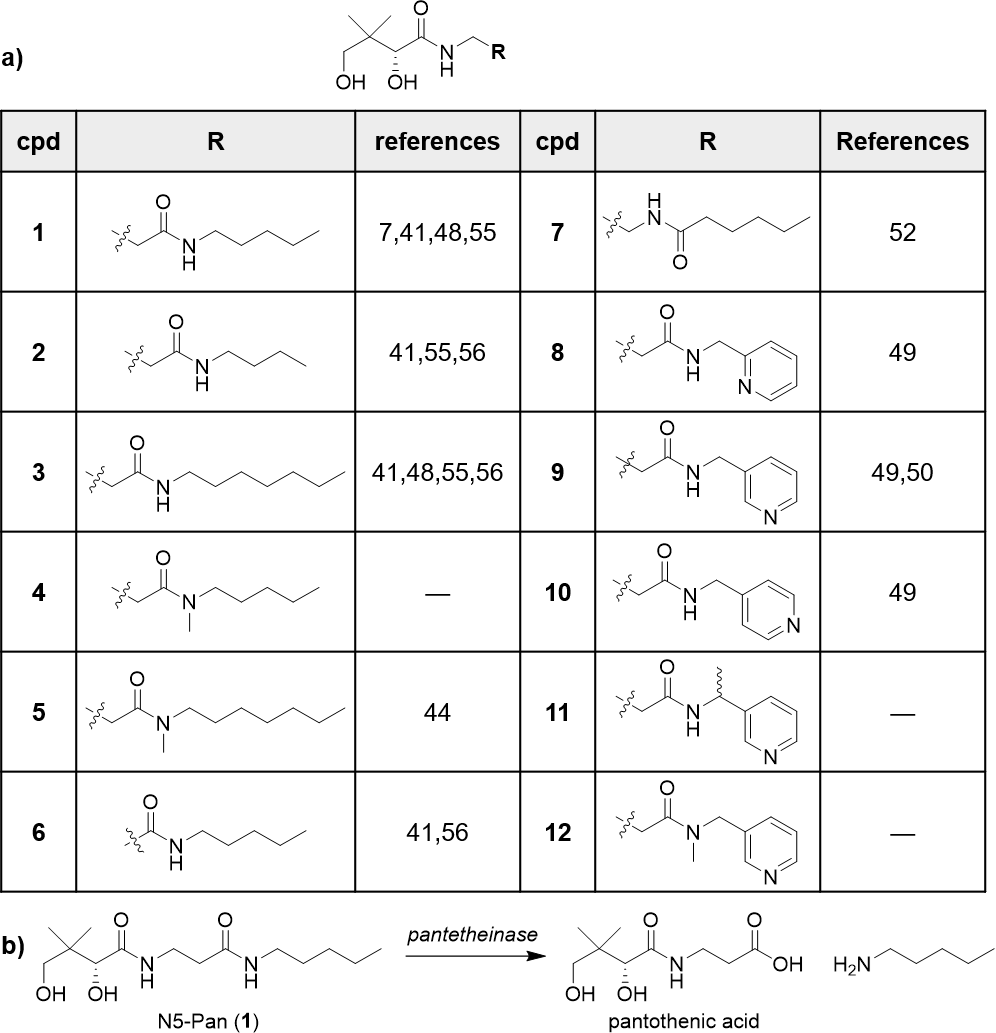
(**a**) Pantothenamides prepared for this study; (**b**) Susceptibility of N5-Pan (**1**) to hydrolysis by pantetheinase.

Pantothenamide activity against laboratory strains of *E. coli* is governed by *N*-alkyl chain length, with long-chain analogs displaying weaker activity due to TolC-mediated efflux (46, 57–60). To determine the effect of alkyl chain length on activity against UPEC, we prepared **1** as well as N4-Pan (**2**) and N7-Pan (**3**, Fig. 2a). Hydrolysis of **1** and **3** by pantetheinases in serum (40, 42) (Fig. 2b) suggests that they will be ineffective antimicrobial agents *in vivo*, which has prompted efforts to develop pantetheinase-resistant analogs with structural modifications near or at the site of amide hydrolysis (41, 44, 52). Thus, we also prepared sterically occluded *N*-methyl N5-Pan **4** (not reported previously), based on *N*-methyl N7-Pan **5**, known to be pantetheinase-resistant yet inactive against *E. coli* (44). In addition, we synthesized the truncated pantetheinase-resistant N5-α-pantothenamide (N5-α-Pan, **6**), which was previously deemed suitable for studies in *in vivo* models of malaria infection (41), as well as the inverse amide of N5-Pan (**7**), which was previously found to be resistant to pantetheinase (52); to our knowledge, **7** has not been previously evaluated against gram-negative bacteria. Finally, we prepared select pantothenamides bearing pyridyl substituents (**8–12**). Analogs **8–10** were previously predicted to engage *E. coli* PanK (*Ec*PanK) via π−π interactions and were found to be substrates for *Ec*PanK (49, 50); however, to our knowledge, their antimicrobial activities and/or susceptibility to pantetheinase-mediated hydrolysis have not been reported. We reasoned that since the turnover efficiencies of analogs **8** and **9** by *Ec*PanK were comparable with **1** (49), they may display comparable activity to N5-Pan against UPEC. Analog **10** was a poorer substrate and thus may display comparably lower antimicrobial activity. Sterically occluded analogs **11** and **12** were designed to be pantetheinase-resistant.

**Fig 3:**
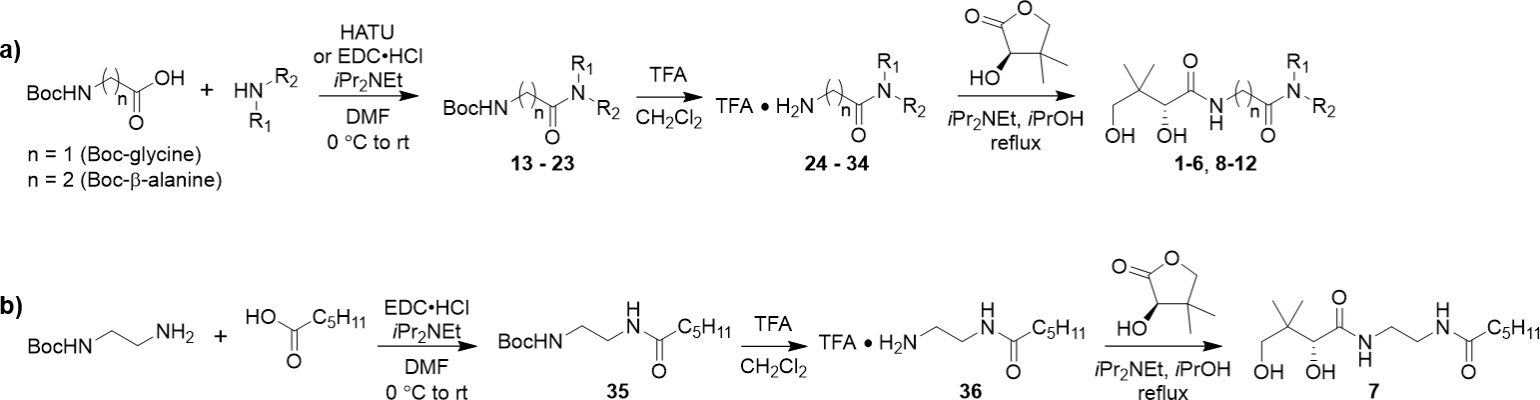
Synthesis of pantothenamide analogs used in this study. (**a**) Synthesis of **1–6** and **8–12**; (**b**) synthesis of inverse amide **7**. Detailed protocols and compound characterization data can be found in Supplemental Information.

Syntheses of **1** and its *N*-substituted derivatives **2–5** and **8–12**, as well as truncated analog **6,** were carried out as shown in Fig. 3a. Boc-β-alanine (*n* = 2, for the synthesis of **1–5** and **8–12**) or Boc-glycine (*n* = 1, for the synthesis of **6**) was coupled to the appropriate primary or *N*-methyl secondary amine using *N*-(3-Dimethylaminopropyl)-*N′*-ethylcarbodiimide hydrochloride (EDC•HCl) or hexafluorophosphate azabenzotriazole tetramethyl uronium (HATU) in the presence of diisopropylethylamine (*i*Pr_2_NEt) to give the corresponding amides (**13–23**) in 90%–97% yield. Removal of Boc protecting groups in the presence of trifluoroacetic acid (TFA) afforded amines **24–34** as the TFA salts (55%–99% yield), which were subsequently reacted with d-(-)-pantolactone under basic conditions (53) to give **1–6** and **8–12** (8%–93% yield). Synthesis of the inverse amide of N5-Pan (**7**) was carried out in a similar manner starting from hexanoic acid and *N*-Boc-ethylenediamine (Fig. 3b) using EDC•HCl.

### Antimicrobial activity of pantothenamides against UPEC under nutrient limitation

**Fig 4:**
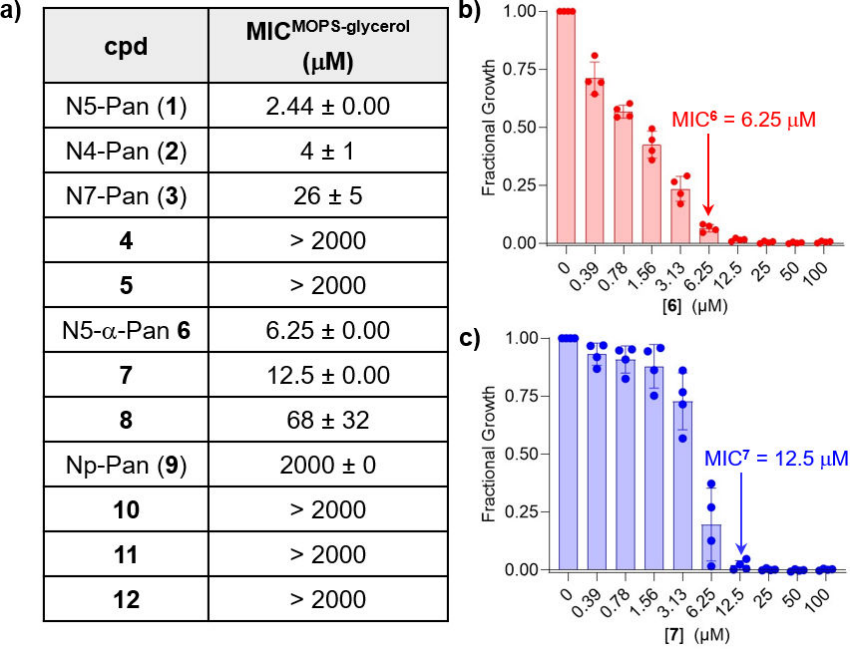
Antimicrobial activity of pantothenamides against UPEC grown in MOPS-glycerol. (**a**) Minimum inhibitory concentrations (MIC) were determined for each pantothenamide against UPEC; standard error in MIC was determined from four experiments. Dose-response data are shown in Fig. S1 and S2 for compounds **1–5** and **8–12**; (**b**) Dose-response for N5-α-Pan **6**; and (**c**) dose-response for inverse amide **7**; error bars represent standard deviation.

The antimicrobial activities of pantothenamides **2–12** were initially evaluated against UPEC strain CFT073 grown in MOPS-glycerol (Fig. 4; Fig. S1 and S2). Minimal inhibitory concentrations (MICs) were determined and compared with the MIC of **1** (Fig. 4a). Analogs **2** and **3**, previously reported to be significantly less potent than **1** against *E. coli* in defined minimal media (46, 57–60), exhibited growth inhibitory activity against UPEC in MOPS-glycerol with MIC values in the low micromolar range. Interestingly, **2** (MIC = 4 µM) displayed comparable activity with **1** (MIC = 2.44 µM), and **3** (MIC = 26 µM) was ~10-fold less potent than **1**. *N*-Methyl N5-Pan analog **4**, expected to have increased stability to pantetheinase compared with **1**, lacked activity against UPEC up to 2 mM. *N*-Methyl N7-Pan analog **5** was similarly inactive. Truncated analog **6** and inverse amide **7** exhibited growth inhibitory activity in the low micromolar range, comparable with **1** (MIC of **6** = 6.25 µM; MIC of **7** = 12.5 µM, Fig. 4b and c). Although pyridyl analogs **8–10** are substrates for *Ec*PanK, only **8** displayed activity against UPEC (MIC = 68 µM) with a ~ 28-fold increase in MIC compared with **1**.

### Metabolic stability of pantetheinase-resistant pantothenamides 6 and 7

**Fig 5:**
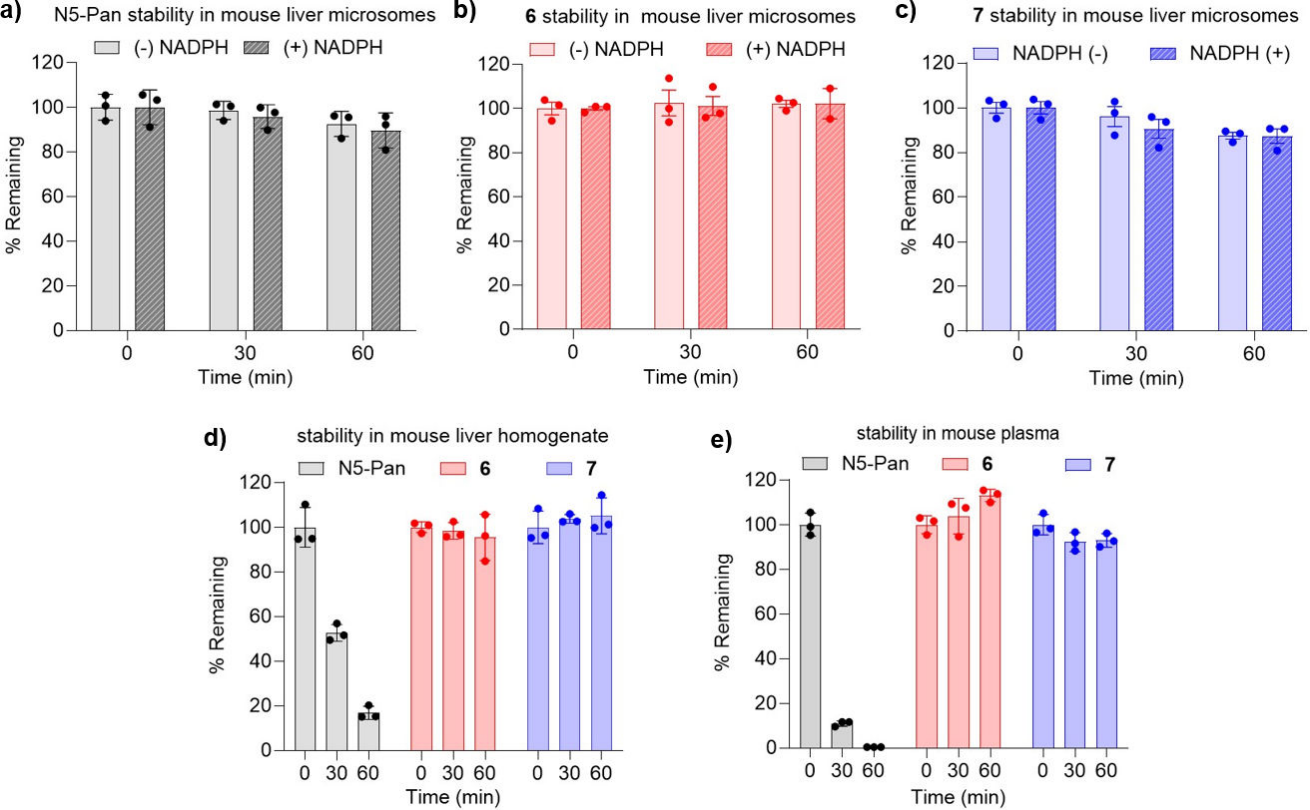
Metabolic stability of N5-Pan (**1**), N5-α-Pan (**6**), and inverse amide **7** in mouse liver microsomes, liver homogenate, and plasma. Pantothenamides **1** (**a**), **6** (**b**), and **7** (**c**) demonstrate good stability in mouse liver microsomes. In mouse liver homogenate (**d**), **1** is unstable, whereas **6** and **7** show good stability. Similarly, in mouse plasma (**e**), **1** is unstable, whereas **6** and **7** show good stability; *n* = 3, error bars represent standard deviation. Replicate data are shown in Tables S1 to S3.

As the most potent pantetheinase-resistant analogs against UPEC in MOPS-glycerol, **6** and **7** are potential candidates for *in vivo* studies exploring combination therapies for UTI targeting DXPS and CoA biosynthesis. Thus, they were evaluated to determine their stability to phase I liver metabolism as well as hydrolysis in liver homogenate and plasma. Each compound was incubated in mouse liver microsomes, mouse liver homogenate (10% wt/vol), or mouse plasma (Fig. 5; Tables S1 to S3). Analog **6** showed good stability in mouse liver microsomes (no degradation at 1 h in the presence or absence of NADPH, Fig. 5b), mouse liver homogenate (96% remaining at 1 h, Fig. 5d), and mouse plasma (no degradation at 1 h, Fig. 5e). Analog **7** also demonstrated good stability in mouse liver microsomes (87% remaining at 1 h in the presence or absence of NADPH, Fig. 5c), mouse liver homogenate (no degradation at 1 h, Fig. 5d), and mouse plasma (93% remaining at 1 h, Fig. 5e). For comparison, the stability of **1** in all three matrices was also determined; **1** demonstrated good stability in mouse liver microsomes (90% and 93% remaining at 1 h in the presence or absence of NADPH, respectively, Fig. 5a). However, **1** degraded rapidly in mouse liver homogenate (17% remaining at 1 h, Fig. 5d) and mouse plasma (<1% remaining at 1 h, Fig. 5e), as expected, due to hydrolysis of the amide linkage.

### Antimicrobial activities of 6 and 7 against UPEC in urine

**Fig 6:**
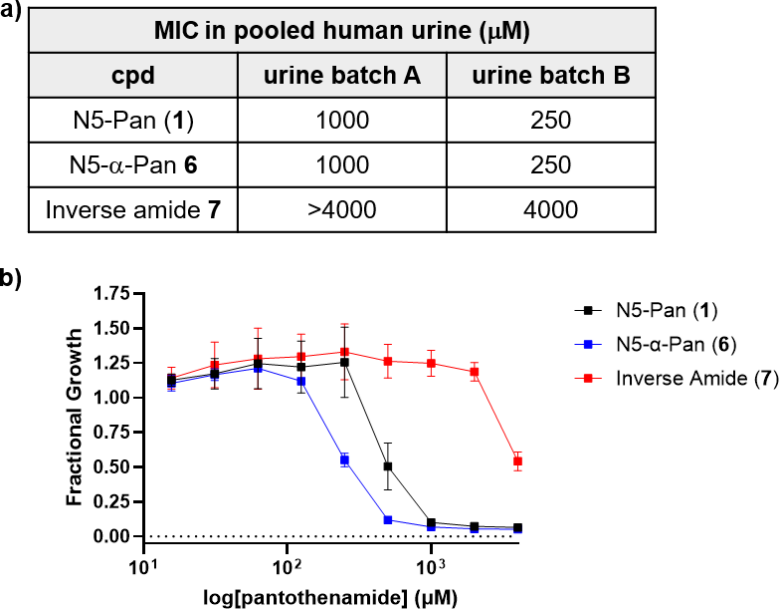
Antimicrobial activity of N5-Pan (**1**), N5-α-Pan (**6**), and inverse amide **7** against UPEC grown in pooled human urine. (**a**) MIC values determined for **1**, **6,** and **7** in two batches of pooled human urine (urine batches A and B); (**b**) dose-responses for **1**, **6,** and **7** against UPEC in urine batch A showing similar potencies for **1** and **6**, and low potency for **7**. Error bars represent standard deviation. MIC determinations for **1**, **6,** and **7** in addition to urine batches C and D can be found in Fig. S3.

As urine represents a relevant growth environment within the urinary tract, pantothenamide potencies measured in this culture condition are expected to more reliably predict *in vivo* antimicrobial activity. Previously, the antimicrobial activity of **1** against UPEC was evaluated in commercial pooled human urine (MIC = 156 µM) (7). However, the urine purchased for the current study did not support UPEC growth. We note that although vendors exclude donors with known infectious diseases, they do not exclude donors who take antimicrobial agents or other supplements that may be excreted through the urinary tract and interfere with bacterial growth. Thus, antimicrobial activities of **6** and **7** were evaluated in pooled human urine that we collected from adult males and females between 24 and 57 years old, who had not used anti-infectives or cranberry products for at least 7 days prior to collection (as described in Methods). The MIC of **1** was in the range 250–1,000 µM in four batches of pooled urine (MIC = 250 µM in urine batch B; MIC = 1,000 µM in urine batches A, C, and D; Fig. 6; Fig. S3), higher than we previously reported, with the measured MIC dependent on the batch of pooled urine used. The variability in MICs is not surprising and is likely attributed to differences in nutrient content that depend on a variety of factors including diet. Truncated analog (**6**) displayed antimicrobial activity against UPEC comparable with **1** in urine, with the MIC in the range 250–1,000 µM in four batches of pooled urine (MIC = 250 µM in urine batch B; MIC = 500 µM in urine batches C and D; MIC = 1,000 µM in urine batch A; Fig. 6; Fig. S3); these MICs are significantly higher (>40-fold) than the MIC of **6** in MOPS-glycerol (Fig. 4), following a similar trend to **1** with regard to growth medium dependence of activity (7). Interestingly, **7** displayed significantly weaker antimicrobial activity against UPEC grown in urine (MIC ≥4,000 µM in all urine batches) despite its comparable potency to **6** in MOPS-glycerol.

### BAP-treated UPEC are sensitized to 6

**Fig 7:**
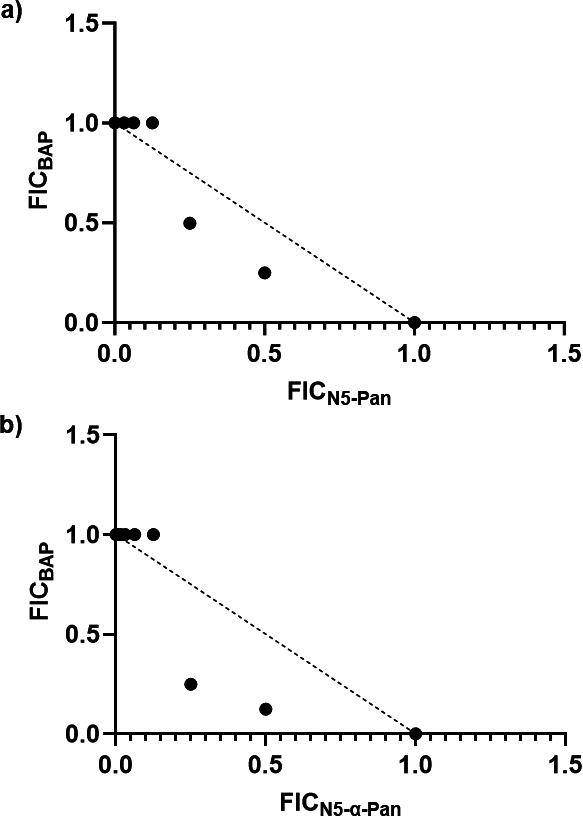
Isobologram analyses showing BAP potentiates the activity of N5-Pan (**a**) and N5-α-Pan (**b**). Checkerboard analyses are shown in Fig. S4 and S5.

Given that **6** showed activity against UPEC in urine and displayed good stability in mouse plasma and liver, we sought to determine if UPEC grown in human urine are sensitized to **6** in the presence of BAP, as previously shown for **1** (7). Thus, a checkerboard analysis was performed to assess the relationship between BAP and **6**. UPEC grown in urine (batch B) was treated with a range of concentrations of BAP and **6** in combination, and fractional inhibitory concentration index (*FIC_I_*) was determined for each combination. The results indicate a clear shift in the potency of **6** in the presence of BAP with *FIC_I_* in the range of 0.5–1.25 across experiments, demonstrating that BAP treatment potentiates the activity of **6**, similar to **1** (Fig. 7; Fig. S4 and S5).

## DISCUSSION

Processes required for bacterial adaptation in the host represent potential antimicrobial targets. Our prior work demonstrated that inhibiting the bacterial central metabolic enzyme DXPS not only impairs the adaptation of UPEC to components in urine but also creates a metabolic vulnerability in this condition, sensitizing UPEC to the prototypical *N*-pentyl pantothenamide inhibitor of CoA biosynthesis, N5-Pan (**1**). This finding suggests a potential new antimicrobial strategy—co-targeting of DXPS and a second pathway that relies on or works in concert with DXPS-dependent vitamin (or isoprenoid) synthesis in a pathogen- and/or growth medium-dependent manner. Testing this approach *in vivo* is an important next step. However, evaluating the *in vivo* efficacy of **1** in combination with BAP will be limited by the susceptibility of **1** to enzymatic hydrolysis. Here, we sought to investigate other pantothenamides against UPEC to identify analogs with enhanced potency and/or hydrolytic stability.

Of the analogs tested in a defined nutrient-limited medium (MOPS-glycerol), none were more potent than **1**, and several analogs were found to have little or no activity in this condition. Our study of the effect of alkyl chain length on the potency of pantothenamides against UPEC revealed different trends than previously reported for pantothenamides against *E. coli* strains. Along with **1**, the longer N7 pantothenamide prototype also serves as an important comparator as this analog was previously shown to be inactive against a laboratory strain of *E. coli*, due to efflux via the TolC transporter (46, 59, 60). However, our study and early studies by Clifton et al. (57) found it to inhibit UPEC growth, albeit with somewhat lower potency compared to **1**. It is plausible that UPEC TolC displays a different specificity for pantothenamides compared with the nonpathogenic strains previously used. Alternatively, differences in TolC expression or activity in different growth conditions could account for the different observed potencies. Interestingly, shortening the alkyl chain (N4 analog) did not significantly reduce pantothenamide potency against UPEC, in contrast to previous findings that this smaller pantothenamide had weaker antimicrobial activity against *E. coli* K12 and *E. coli* 9723 strains compared with **1** (57, 59). These differences in susceptibility between *E. coli* strains underscore the importance of evaluating pantothenamides against the pathogen used for *in vivo* studies.

A truncated analog of **1**, analog **6** (N5-α-Pan), as well as the inverse amide of **1**, emerged from the SAR study as promising leads, given their comparable antimicrobial activity to **1** under defined nutrient limitation conditions and their known resistance to pantetheinase. Both analogs were found to be metabolically stable, indicating good compatibility for future *in vivo* evaluation in the ascending UTI assay. However, only the truncated analog (**6**) displayed antimicrobial activity comparable with **1** in urine, a culture condition that is more representative of the urinary tract environment. The lack of activity observed for inverse amide may reflect decreased permeability in urine, or perhaps differences in potency against the target that are revealed in this condition. On a typical American diet, humans excrete an average of 2.6 mg of pantothenate per day (61, 62). Although urine is considered a relatively nutrient-limited *in vivo* environment, pantothenate and other nutrients in urine could offset pantothenamide-mediated inhibition of CoA biosynthesis; the extent to which this occurs depends on the diets and lifestyles of the subjects who donated urine. These pantothenamides presumably compete with pantothenate on UPEC PanK (60). At high concentrations, the toxicity of **1** is known to be irreversible even in the presence of pantothenate (60). Perhaps the observed decrease in potency against UPEC grown in urine compared with MOPS-glycerol, observed for all three analogs, reflects competition by pantothenate in the urine with the inverse amide potentially exhibiting lower affinity for PanK relative to pantothenate.

Notably, the antimicrobial activity of **1** in urine was found to be variable across four batches of pooled human urine in this study and compared with our previous report (7). The activity of the lead compound, truncated analog **6**, was also variable in different batches of pooled urine and comparable with **1** in each batch. The variability in activity observed in different urine batches is not surprising, given our previous observation that antimicrobial activity of **1** depends upon the growth environment (7), together with the fact that the properties of urine, including pH and metabolic profile, vary between individuals (63, 64).

Inhibitor combination studies conducted in a single batch of pooled human urine showed that, like the combination of BAP and **1**, BAP-treated UPEC are sensitized to **6**. This can be explained by the fact that UPEC depends heavily on PLP and ThDP as well as CoA for a functioning TCA cycle, which supports gluconeogenesis from amino acids in urine. BAP treatment limits PLP and ThDP, which in turn limits the ability of UPEC to replenish TCA cycle intermediates for gluconeogenesis. This metabolic burden makes UPEC more vulnerable to inhibitors of CoA, which is also required for the TCA cycle. In addition, it is likely that d-Ser, also present in urine, builds to toxic levels under conditions of DXPS inhibition, further hindering CoA synthesis and increasing UPEC vulnerability to **6** in the presence of BAP. It is possible that the degree to which BAP potentiates pantothenamide activity against UPEC would also depend on the urine composition. Depending on the diet, lifestyle, hydration status, and health of the subjects who donate urine, the amount of d-Ser, pantothenate, or other metabolic components, and/or differences in pH (65), ionic strength (66), or osmolality (67) could impact the potency and interaction between BAP and pantothenamides. However, for studies in the *in vivo* ascending UTI model, variability of urine composition is less likely to be an issue, given the controlled diet for mice.

Overall, our results indicate that truncation of the N5 pantothenamide scaffold confers resistance to mouse pantetheinase while maintaining activity against UPEC in urine, alone, or in combination with BAP, similar to **1**. Thus, **6** (N5-α-Pan) is a promising candidate for *in vivo* proof-of-concept studies to investigate how a pathogen can become sensitized in the host under conditions of DXPS inhibition. Future experiments using the ascending UTI model will explore how combining BAP and **6** at subtherapeutic doses can enhance the efficacy of either single agent. Such studies will validate this metabolic vulnerability of UPEC *in vivo* and demonstrate DXPS-dependent adaptation as a viable target space, which can be explored in the development of strategies to complement existing therapies to treat or prevent UTI.

## SUPPLEMENTAL MATERIAL

Supplemental material spectrum.03069-24-s0001.pdf Fig. S1 to S6; Tables S1 to S3; compound synthesis and characterization.

ASM does not own the copyrights to Supplemental Material that may be linked to, or accessed through, an article. The authors have granted ASM a non-exclusive, world-wide license to publish the Supplemental Material files. Please contact the corresponding author directly for reuse.
