# Supplementary material for: Exploring the antimicrobial activity of pantothenamides against uropathogenic Escherichia coli
Source: Microbiol Spectr. 2025 Jun 23;13(8):e03069-24. doi: 10.1128/spectrum.03069-24 (PMC12323621; doi:10.1128/spectrum.03069-24)
Supplement: Supplemental material — Fig. S1 to S6; Tables S1 to S3; compound synthesis and characterization. [file spectrum.03069-24-s0001.pdf]

Supplemental Information

Exploring antimicrobial activity of pantothenamides against uropathogenic *Escherichia coli*.

Alicia A. DeColli[1], David J. Meyers[1], Arina Ranjit[2,3], James Paule[2,3], Kimberly M. Bockley[1,4], Laura M. Ensign[1,4-6], Rana Rais[1,2,3] and Caren L. Freel Meyers[1]*

[1]Department of Pharmacology and Molecular Sciences, The Johns Hopkins University School of Medicine, Baltimore, MD, 21205, USA

[2]Department of Neurology, Johns Hopkins University School of Medicine, Baltimore, Maryland, USA

[3]Johns Hopkins Drug Discovery, Johns Hopkins University School of Medicine, Baltimore, MD, USA

[4]Center for Nanomedicine at the Wilmer Eye Institute, Department of Ophthalmology, Johns Hopkins University School of Medicine, Baltimore, MD, USA

[5]Department of Chemical & Biomolecular Engineering, Johns Hopkins University, Baltimore, MD, USA

Department of Biomedical Engineering, Johns Hopkins University, Baltimore, MD, USA

[6]Department of Gynecology and Obstetrics, Johns Hopkins University School of Medicine, Baltimore, MD, USA

Division of Infectious Diseases, Johns Hopkins University School of Medicine, Baltimore, Maryland, USA

Department of Oncology, Johns Hopkins University School of Medicine, Baltimore, MD, USA

*Email: cmeyers@jhmi.edu

Table of Contents **Page**

1. Supplementary Figures and Tables

Figure S1. Averaged dose response curves for compounds **1-5** in MOPS-glycerol 3

Figure S2. Averaged dose response curves for compounds **8-12** in MOPS-glycerol 4

Figure S3. Averaged dose response curves for compounds **1** and **6-7** in urine............................5

Figure S4. Replicate isobolograms and checkerboards for BAP/N5-Pan (**1**) in urine.................6

Figure S5. Replicate isobolograms and checkerboards for BAP/ N5-α-Pan (**6**) in urine 7

Figure S6. Method for analyzing checkerboard data and assembling isobolograms.................. 8

Table S1. Values of % compound remaining in mouse liver microsomes..................................9

Table S2. Values of % compound remaining in mouse liver homogenate..................................9

Table S3. Values of % compound remaining in mouse plasma 9

2. Chemistry

2.1 General Methods 10

2.2 Compound synthesis 11

3. Compound characterization

3.1 ^{1}H NMR spectra, ^{13}C NMR spectra, and high-resolution mass spectrometry (HRMS) for compounds **1-12**..24

3.2 ^{1}H NMR spectra for compounds **13-24** 72

3.3 ^{1}H and ^{13}C NMR spectra for compounds **24-27** 83

3.4 ^{1}H NMR spectra for compounds **28-29** 91

3.5 ^{1}H and ^{13}C NMR spectra for compounds **30-33** 93

3.6 ^{1}H NMR spectra for compounds **34-36** 101

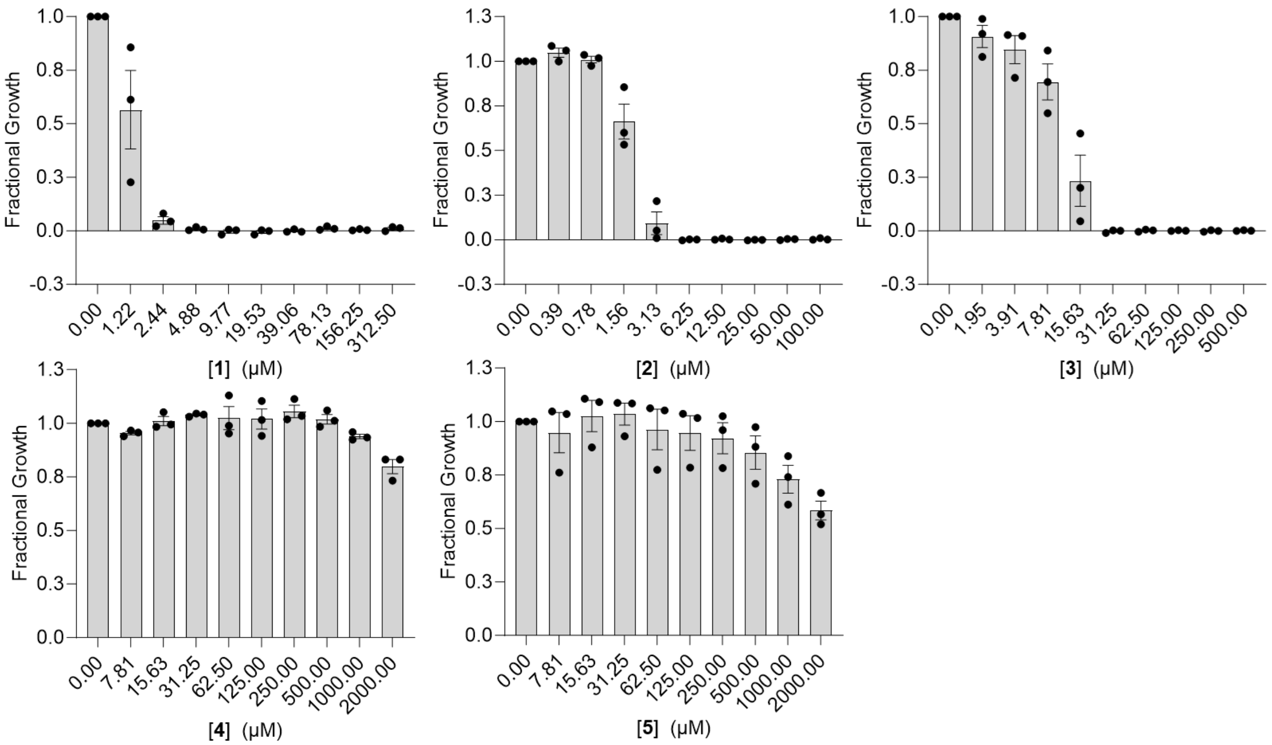

Figure S1. Averaged dose response curves for compounds **1-5** in MOPS-glycerol. Experiments performed in biological triplicate, error bars represent standard error of the mean.

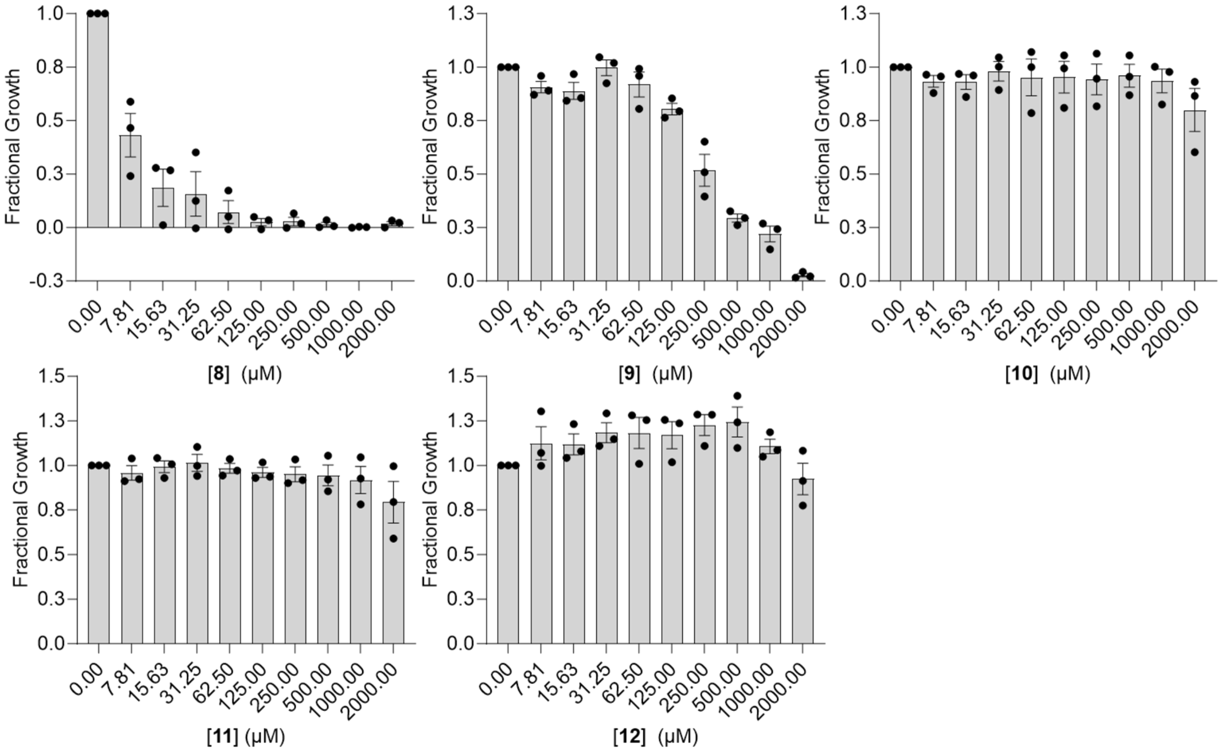

Figure S2. Averaged dose response curves for compounds **8-12** in MOPS-glycerol. Experiments performed in biological triplicate, error bars represent standard error of the mean.

a)

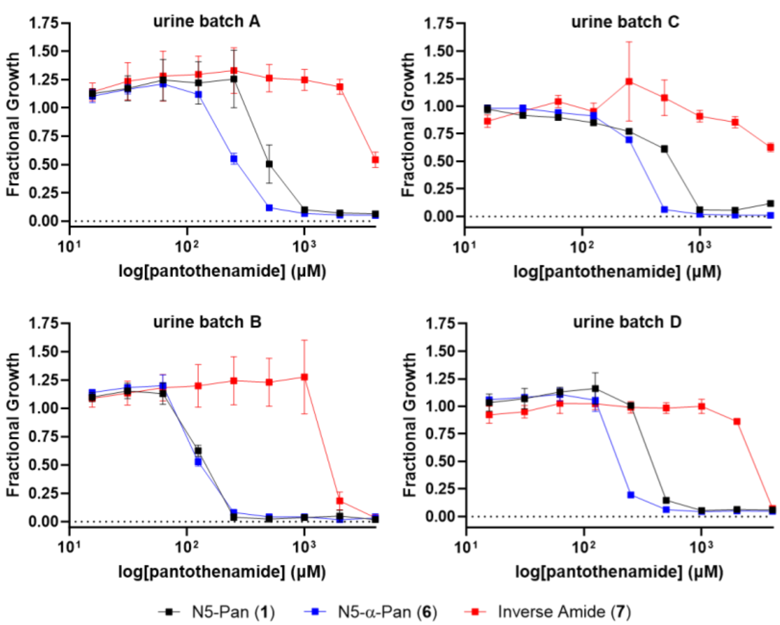

b)

MIC in pooled human urine (μM)				
cpd	urine batch A	urine batch B	urine batch C	urine batch D
N5-Pan (**1**)	1000	250	1000	1000
N5-α-Pan **6**	1000	250	500	500
Inverse amide **7**	>4000	4000	>4000	4000

c)

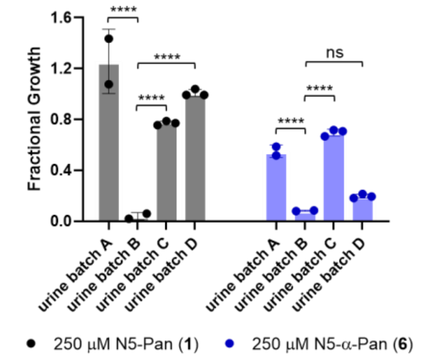

Figure S3. Activity of **1**, **6** and **7** in pooled human urine. **a)** Averaged dose response curves for compounds **1**, and **6-7** in pooled human urine batches A-D. Compounds were evaluated up to 4 mM; **b)** Minimum inhibitory concentrations (MICs) determined for **1**, **6**, and **7** in pooled human urine; **c)** The potencies of **1** and **6** vary in different batches of pooled human urine. Fractional growth of UPEC grown in urine batches A-D in the presence of **1** or **6** at 250 μM is shown to illustrate statistically significant differences is compound potency. Experiments performed in biological duplicate (batches A & B) or triplicate (batched C & D), error bars represent standard deviation. $p > 0.05$ (ns); $p \leq 0.0001$ (****)

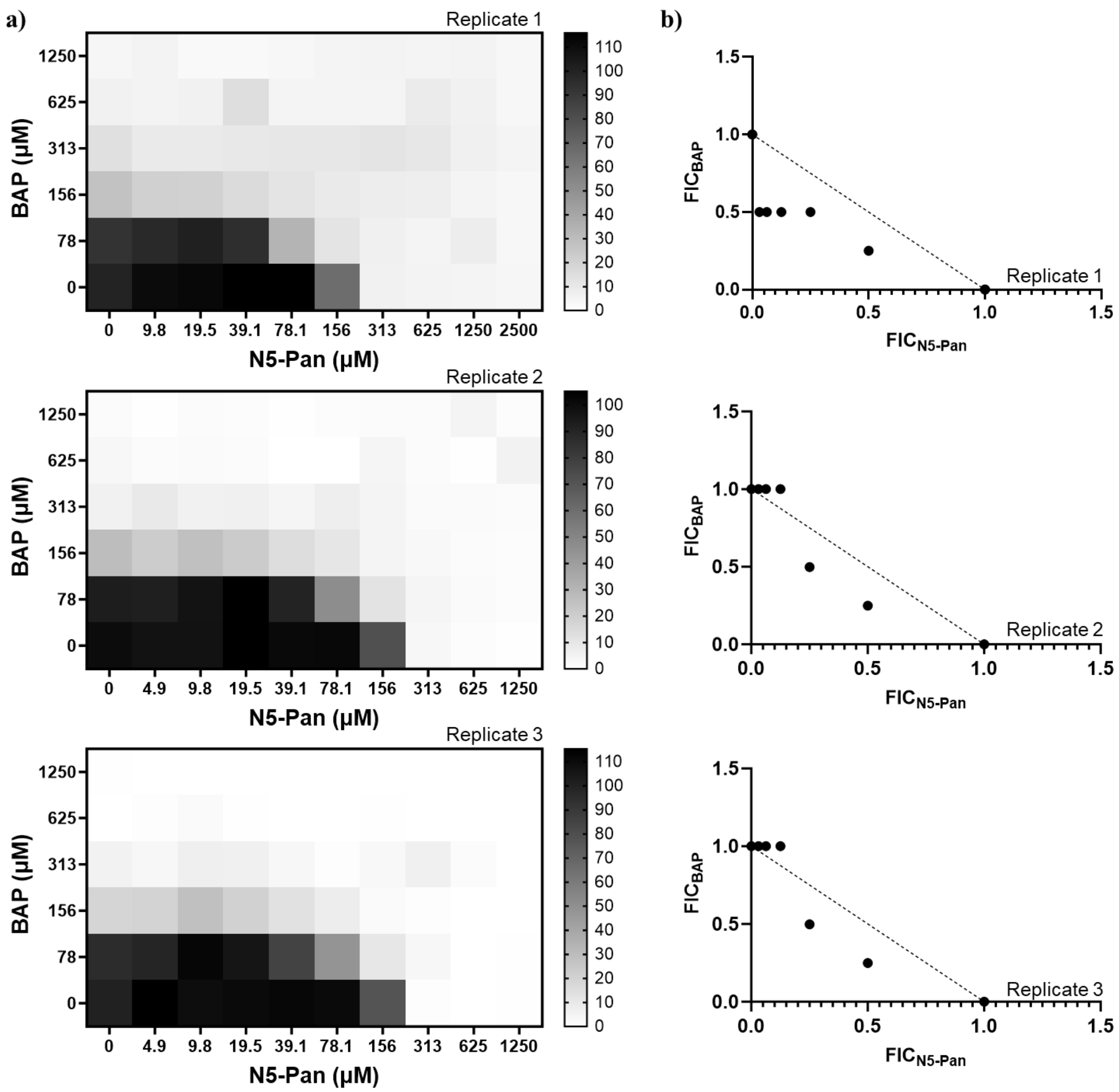

Figure S4. Replicate isobolograms and checkerboards for BAP/N5-Pan (**1**) in urine Batch B. **a)** Replicate checkerboard analysis with heat map indicating fractional growth at each BAP/N5-Pan combination; **b)** corresponding replicate isobolograms; dotted line represents the line of additivity where $FIC_I < 1.0$; Replicate 2 is shown in Figure 6a.

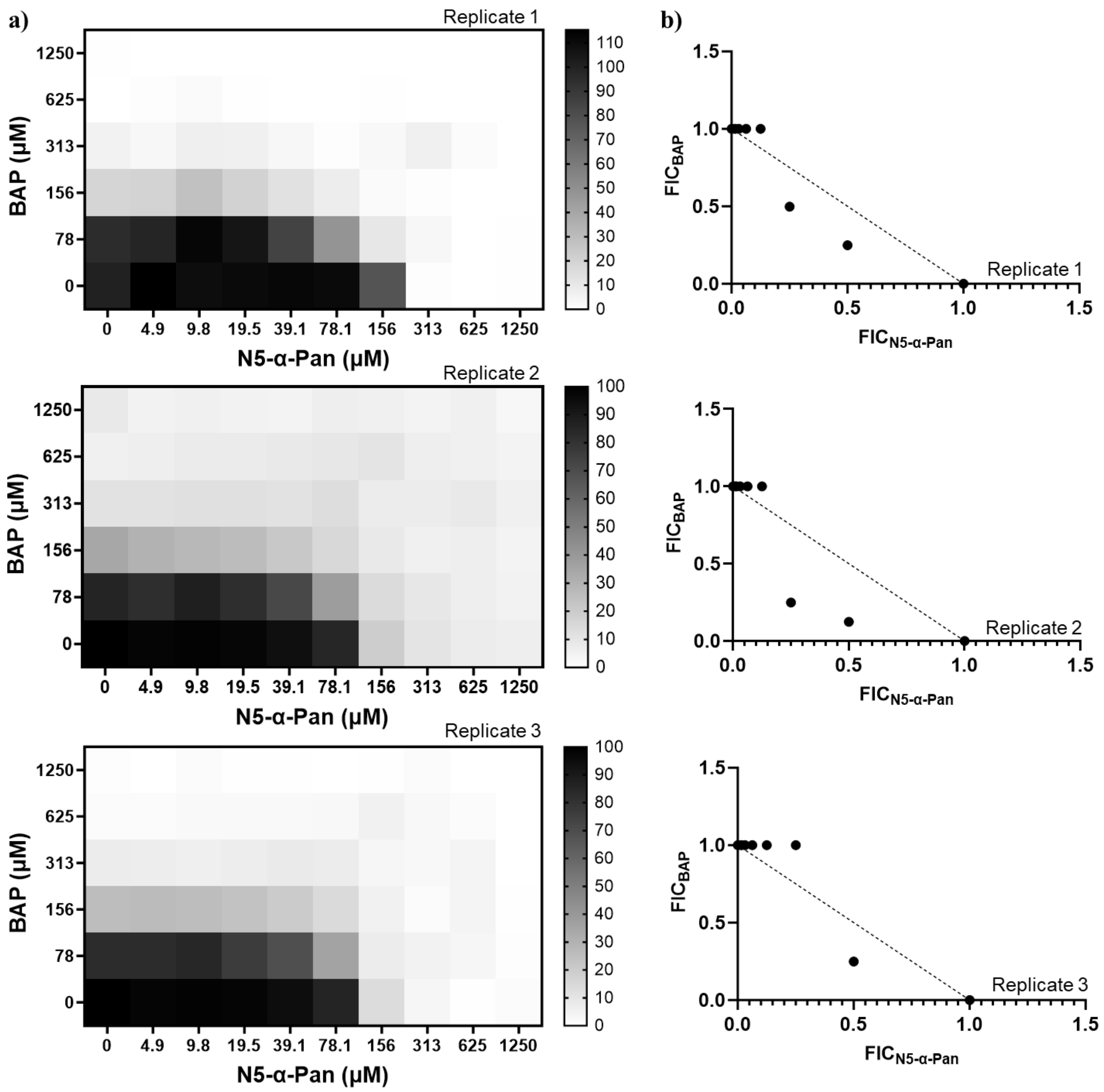

Figure S5. Replicate isobolograms and checkerboards for BAP/ N5-α-Pan (**6**) in urine Batch B. **a)** Replicate checkerboard analysis with heat map indicating fractional growth at each BAP/ N5-α-Pan (**6**) combination; **b)** corresponding replicate isobolograms; dotted line represents the line of additivity where $FIC_I < 1.0$; Replicate 2 is shown in Figure 6b.

$$\text{FIC index (FIC}_I\text{)} = \text{FIC}_A + \text{FIC}_B$$

$$= ([A]/\text{MIC}_A) + ([B]/\text{MIC}_B)$$

$$= (156\ \mu\text{M}/313\ \mu\text{M}) + (156\ \mu\text{M}/625\ \mu\text{M})$$

$$= 0.5 + 0.25$$

$$\text{FIC}_I = 0.75$$

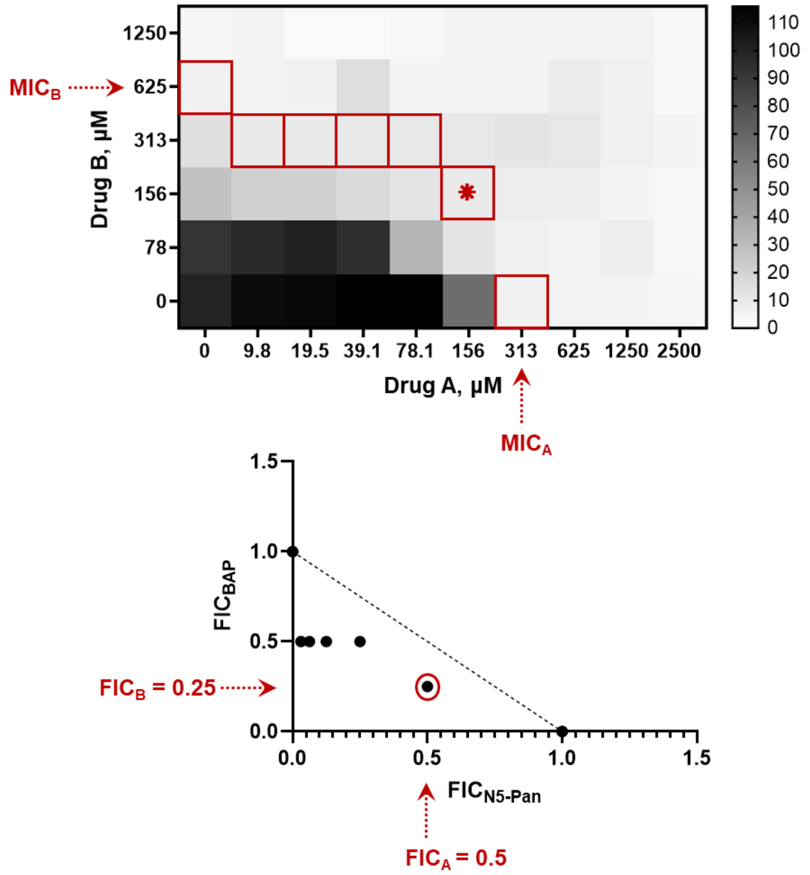

Figure S6. Method for analyzing checkerboard data and assembling isobolograms. Red boxes indicate wells with < 10% bacterial growth compared to the (-) drug control, over the range of Drug A concentrations tested in combination with Drug B. Calculation of FIC_A, FIC_B and FIC_I is shown for a selected drug combination (✱). This combination of Drug A & Drug B is represented by a single point on the isobologram (circled in red), where $\text{FIC}_A = 0.5$ and $\text{FIC}_B = 0.25$. This analysis was repeated for each growth inhibitory drug combination of the checkerboard analysis (wells boxed in red), to generate an isobologram. The FIC_I for this particular combination of Drug A and Drug B is 0.75. The FIC_I range for a given checkerboard analysis was determined by calculating FIC_I for each combination notated by the red boxes.

Phase-1 Mouse Liver Microsomal Stability						
Time (min)	N5-Pan		N5-α-Pan 6		Inverse amide 7	
	+NADPH (replicates) mean	-NADPH (replicates) mean	+NADPH (replicates) mean	-NADPH (replicates) mean	+NADPH (replicates) mean	-NADPH (replicates) mean
0	(91.15, 105.60, 103.26) 100	(93.93, 100.68, 105.39) 100	(98.24, 100.95, 100.82) 100	(94.49, 101.59, 103.92) 100	(101.64, 94.61, 103.74) 100	(101.57, 103.15, 95.28) 100
30	(99.92, 97.67, 89.83) 95.81	(100.54, 101.36, 93.98) 98.62	(95.94, 109.85, 97.84) 101.21	(93.90, 99.99, 113.71) 102.53	(82.15, 93.65, 95.89) 90.56	(87.66, 97.78, 102.74) 96.06
60	(95.93, 92.19, 80.92) 89.68	(95.50, 96.15, 86.09) 92.58	(95.33, 109.11) 102.22	(99.04, 104.56, 102.67) 102.09	(80.85, 90.51, 90.62) 87.33	(84.58, 89.43, 88.50) 87.50

Table S1. Values of % compound remaining in mouse liver microsomes.

Stability in Mouse Liver Homogenate			
Time (min)	N5-Pan (replicates) mean	N5-α-Pan 6 (replicates) mean	Inverse amide 7 (replicates) mean
0	(94.74, 110.24, 94.88) 99.96	(101.73, 100.96, 97.31) 100	(96.38, 108.38, 95.24) 100
30	(51.66, 56.88, 49.49) 52.68	(96.55, 102.55, 95.80) 98.30	(102.45, 105.95, 102.69) 103.70
60	(15.10, 20.33, 15.27) 16.90	(84.83, 96.15, 105.58) 95.52	(101.22, 99.77, 114.39) 105.13

Table S2. Values of % compound remaining in mouse liver homogenate.

Stability in Mouse Plasma			
Time (min)	N5-Pan (replicates) mean	N5-α-Pan 6 (replicates) mean	Inverse amide 7 (replicates) mean
0	(105.50, 95.38, 99.13) 100	(101.57, 102.99, 95.43) 100	(98.30, 96.53, 105.18) 100
30	(11.60, 9.63, 11.64) 10.95	(94.61, 109.22, 107.52) 103.78	(96.70, 91.62, 88.28) 92.20
60	(0.41, 0.36, 0.41) 0.41	(113.80, 109.99, 115.39) 113.06	(92.67, 90.05, 96.12) 92.95

Table S3. Values of % compound remaining in mouse plasma.

2. Chemistry

2.1 General Methods. All commercially available reagents and solvents were used without further purification unless otherwise stated. Automated flash chromatography was performed on a Teledyne Isco CombiFlash Rf+ or Grace Reveleris using Teledyne Isco, Grace or Buchi flash silica, and/or C18 flash cartridges. Spectra were recorded on a Jeol JNM-ECZL500R spectrometer equipped with a Royal HFX probe (^{1}H NMR at 500 MHz and ^{13}C NMR at 125 MHz) at 296 K in $CDCl_3$ (^{1}H NMR referenced to 7.27 ppm, ^{13}C NMR referenced to 77.00 ppm), CD_3OD (^{1}H NMR referenced to 3.31 ppm, ^{13}C NMR referenced to 49.15 ppm) or D_2O (4.75 ppm). Analytical LC/MS was performed using an Agilent 1260 equipped with autosampler (Agilent Poroshell 120 C18 column (50 mm x 4.6 mm i.d., 3.5 μm); 0.05% TFA in water/acetonitrile gradient; UV detection at 215 and 254 nm) and electrospray ionization. All final compounds showed purity greater than 95% at 215 and 254 nm using this method.

Abbreviations used:

DMF = *N*,*N*-dimethylformamide
equiv. = equivalent
EtOAc = ethyl acetate
HATU = 1-[Bis(dimethylamino)methylene]-1H-1,2,3-triazolo[4,5-b]pyridinium 3-oxid hexafluorophosphate
NMP = 1-methyl-2-pyrrolidinone
Rt = retention time
rt = room temperature
TFA = trifluoroacetic acid

2.2 Compound synthesis.

General procedure for amide formation using the synthesis of *tert*-butyl (3-oxo-3-(pentylamino)propyl)carbamate **13** as an example:

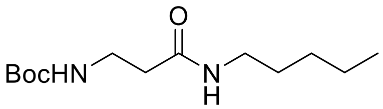

To a solution of BOC-β-alanine (1139.0 mg, 6.020 mmol, 1.0 equiv.) and HATU (2346.6 mg, 6.170 mmol, 1.025 equiv.) in anhydrous DMF (12 ml, ca. 0.5 M) was added *N*,*N*-diisopropylethylamine (1.57 ml, 9.03 mmol, 1.5 equiv.) in one portion at 0°C. After stirring at 0°C for ca. 1 min., the solution was allowed to stir at rt for 5 min. The solution was then again cooled to 0°C and 1-aminopentane (0.837 ml, 7.224 mmol, 1.2 equiv.) was added in one portion. After addition, the ice/water cooling bath was removed and the reaction was allowed to stir at rt. After stirring at rt for 24 h, the reaction was diluted with water, ethyl acetate and sat. aq. Na_2CO_3. The mixture was extracted with EtOAc (3 x 20 ml), and the organic layers were combined, washed with 5% aq. HCl, brine and dried with anhydrous $MgSO_4$. Note: the HCl wash was omitted for compounds containing a pyridyl motif. The volatiles were removed in vacuo, and the resulting residue was absorbed onto silica gel and purified via silica gel flash chromatography (gradient 0-100% hexanes/10% methanol in ethyl acetate) to provide 1.674 g light yellow solid (107% yield). The product was contaminated with DMF and tetramethylurea (2.807 ppm, s, 12H in $CDCl_3$) and used in the next step without further purification. ^{1}H NMR (500 MHz, CHLOROFORM-d) δ 5.68 (br. s., 1H), 5.17 (br. s., 1H), 3.40 (q, J = 6.02 Hz, 2H), 3.24 (q, J = 6.87 Hz, 2H), 2.39 (t, J = 5.87 Hz, 2H), 1.50 (quin, J = 7.23 Hz, 2H), 1.43 (s, 10H), 1.22 - 1.39 (m, 4H), 0.90 (t, J = 7.02 Hz, 3H), Molecular Weight: 258.36.

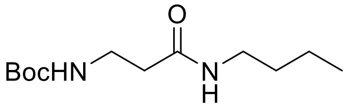

Tert-butyl (3-(butylamino)-3-oxopropyl)carbamate **14** was synthesized using the **General procedure for amide formation** and BOC-β-alanine and butan-1-amine as the coupling partners to provide 1.4406 g white solid (110% yield). NMP replaced DMF in the general procedure. The product was contaminated with water and used in the next step without further purification. ^{1}H NMR (500 MHz, CHLOROFORM-d) δ 5.62 (br. s., 1H), 5.14 (br. s., 1H), 3.39 (q, J = 6.30 Hz, 2H), 3.24 (dt, J = 5.73, 7.16 Hz, 2H), 2.37 (t, J = 5.87 Hz, 2H), 1.44 - 1.51 (m, 2H), 1.42 (s, 9H), 1.26 - 1.38 (m, 2H), 0.91 (t, J = 7.30 Hz, 3H). Molecular Weight: 244.34

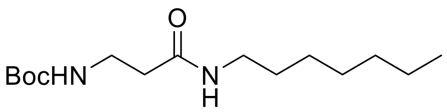

Tert-butyl (3-(heptylamino)-3-oxopropyl)carbamate **15** was synthesized using the **General procedure for amide formation** and BOC-β-alanine and 1-aminoheptane as the coupling partners to provide 1.5549 g white solid (96.9% yield). The product was contaminated with tetramethylurea and used in the next step without further purification. ^{1}H NMR (500 MHz, CHLOROFORM-d) δ 5.65 (br. s., 1H), 5.16 (br. s., 1H), 3.31 - 3.48 (m, 2H), 3.13 - 3.31 (m, 2H), 2.23 - 2.46 (m, 2H), 1.48 (d, J = 6.30 Hz, 2H), 1.43 (s, 10H), 1.16 - 1.36 (m, 9H), 0.79 - 0.96 (m, 3H) Molecular Weight: 286.42

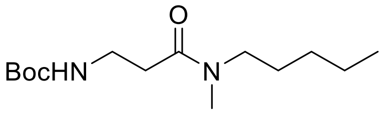

Tert-butyl (3-(methyl(pentyl)amino)-3-oxopropyl)carbamate **16** was synthesized using the **General procedure for amide formation** and BOC-β-alanine and *N*-methylpentylamine as the coupling partners to provide 1.3315 g clear colorless oil (90% yield). ^{1}H NMR (500 MHz, CHLOROFORM-d) δ 5.36 (br. s., 1H), 3.38 - 3.47 (m, 2H), 3.30 - 3.38 (m, 1H), 3.16 - 3.27 (m, 1H), 2.93 (d, J = 11.74 Hz, 3H), 2.49 (td, J = 5.59, 11.17 Hz, 2H), 1.52 (dt, J = 7.73, 15.75 Hz, 2H), 1.42 (s, 9H), 1.33 (quin, J = 7.16 Hz, 2H), 1.20 - 1.30 (m, 2H), 0.90 (dt, J = 5.59, 7.09 Hz, 3H), Molecular Weight: 272.39

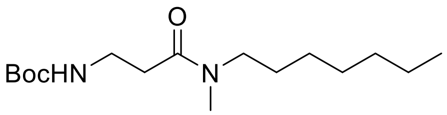

Tert-butyl (3-(heptyl(methyl)amino)-3-oxopropyl)carbamate **17** was synthesized using the **General procedure for amide formation** and BOC-β-alanine and *N*-methylheptan-1-amine as the coupling partners to provide 1.5963 g clear colorless oil (96.3% yield). The product was contaminated with trace tetramethylurea and used in the next step without further purification. ^{1}H NMR (500 MHz, CHLOROFORM-d) δ 5.36 (br. s., 1H), 3.38 - 3.49 (m, 2H), 3.30 - 3.38 (m, 1H), 3.16 - 3.27 (m, 1H), 2.93 (d, J = 11.74 Hz, 3H), 2.49 (td, J = 5.59, 11.74 Hz, 2H), 1.45 - 1.58 (m, 2H), 1.43 (s, 9H), 1.28 (d, J = 4.58 Hz, 8H), 0.81 - 0.95 (m, 3H), Molecular Weight: 300.44

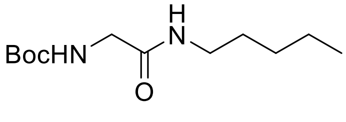

Tert-butyl (2-oxo-2-(pentylamino)ethyl)carbamate **18**. To a solution of Boc-Gly-OH (1177.7 mg, 6.723 mmol, 1.0 equiv.), EDC•HCl (1933 mg, 10.084 mmol, 1.5 equiv.) and HOBt•H2O (659.2 mg, 6.723 mmol, 1.0 equiv.) in anhydrous DMF (6.7 ml, ca. 1 M) was added 1-aminopentane (1.56 ml, 13.45 mmol, 2.0 equiv.) in one portion at rt. After stirring at rt for 24 h, the reaction was

diluted with water, ethyl acetate and sat. aq. Na_2CO_3. The mixture was extracted with EtOAc (3 x 20 ml), and the organic layers were combined, washed with 5% aq. HCl, brine and dried with anhydrous $MgSO_4$. The volatiles were removed in vacuo, and the resulting residue was purified via reverse phase C18 flash chromatography (gradient 0-100% H2O + 0.05% TFA/MeCN + 0.05% TFA) to provide to provide 1.49 g pale yellow foam after lyophilization (90.7% yield). ^{1}H NMR (500 MHz, METHANOL-d4) δ 3.66 (s, 2H), 3.19 (t, J = 7.02 Hz, 2H), 1.50 (quin, J = 7.09 Hz, 2H), 1.45 (s, 10H), 1.24 - 1.41 (m, 4H), 0.91 (t, J = 7.02 Hz, 3H). Molecular Weight: 244.34

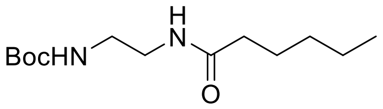

Tert-butyl (2-hexanamidoethyl)carbamate **35**. To a solution of hexanoic acid (0.54 ml, 4.304 mmol, 1.0 equiv.), EDC•HCl (907.7 mg, 4.735 mmol, 1.1 equiv.) and HOBt•H_2O (659.2 mg, 4.304 mmol, 1.0 equiv.) in anhydrous DMF (4.3 ml, ca. 1 M) was added *N,N*-diisopropylethylamine (1.87 ml, 10.76 mmol, 2.5 equiv.) dropwise at 0°C. After addition was complete, the ice/water bath was removed and the reaction allowed to stir at rt. After stirring for 5 min, *t*-butyl (2-aminoethyl)carbamate (0.82 ml, 5.17 mmol, 1.2 equiv.) was added in one portion at rt. After stirring for 24 h, the reaction was diluted with water, ethyl acetate and sat. aq. Na_2CO_3. The mixture was extracted with EtOAc (3 x 20 ml), and the organic layers were combined, washed with 5% aq. HCl, brine and dried with anhydrous $MgSO_4$. The volatiles were removed in vacuo, and the resulting residue was absorbed onto silica gel and purified via silica gel flash chromatography (gradient 0-100% hexanes/10% methanol in ethyl acetate) to provide 0.611 g clear colorless viscous oil (55% yield). ^{1}H NMR (500 MHz, METHANOL-d4) δ 3.23 (t, J = 6.16 Hz, 2H), 3.14 (t, J = 6.16 Hz, 2H), 2.17 (t, J = 7.59 Hz, 2H), 1.60 (quin, J = 7.52 Hz, 2H), 1.43 (s, 10H), 1.24 - 1.39 (m, 4H), 0.92 (t, J = 7.16 Hz, 3H). Molecular Weight: 258.36

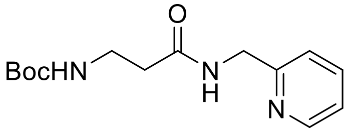

Tert-butyl (3-oxo-3-((pyridin-2-ylmethyl)amino)propyl)carbamate **19** was synthesized using the **General procedure for amide formation** and BOC-β-alanine and 2-(aminomethyl)pyridine as the coupling partners to provide 1.6005 g pale yellow oil (106.4% yield). The product was contaminated with DMF and used in the next step without further purification. ^{1}H NMR (500 MHz, CHLOROFORM-d) δ 8.55 (d, J = 4.87 Hz, 1H), 7.67 (dt, J = 1.72, 7.59 Hz, 1H), 7.26 (d, J = 7.73 Hz, 1H), 7.18 - 7.24 (m, 1H), 6.81 (br. s., 1H), 5.44 (br. s., 1H), 4.58 (d, J = 4.87 Hz, 2H), 3.46 (q, J = 5.92 Hz, 2H), 2.51 (t, J = 5.87 Hz, 2H), 1.43 (s, 9H), Molecular Weight: 279.34

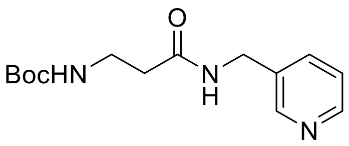

Tert-butyl (3-oxo-3-((pyridin-3-ylmethyl)amino)propyl)carbamate **20** was synthesized using the **General procedure for amide formation** and BOC-β-alanine and 3-(aminomethyl)pyridine as the coupling partners to provide 1.508 g of a clear colorless oil that slowly solidified to white solid (97.5% yield). The product was contaminated with DMF and an unknown and used in the next step without further purification. ^{1}H NMR (500 MHz, CHLOROFORM-d) δ 8.44 - 8.58 (m, 2H), 7.64 (d, J = 7.73 Hz, 1H), 7.27 - 7.30 (m, 1H), 6.36 (br. s., 1H), 5.14 (br. s., 1H), 4.46 (d, J = 6.02 Hz, 2H), 3.43 (q, J = 6.11 Hz, 2H), 2.47 (t, J = 5.87 Hz, 2H), 1.41 (s, 9H), Molecular Weight: 279.34

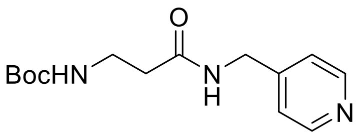

Tert-butyl (3-oxo-3-((pyridin-4-ylmethyl)amino)propyl)carbamate **21** was synthesized using the **General procedure for amide formation** and BOC-β-alanine and 4-(aminomethyl)pyridine as the coupling partners to provide 1.867 g pale yellow viscous oil (123.4% yield). The product was contaminated with DMF and EtOAc and used in the next step without further purification. ^{1}H NMR (500 MHz, CHLOROFORM-d) δ 8.56 (dd, J = 1.86, 4.44 Hz, 2H), 7.11 - 7.24 (m, 2H), 6.36 (br. s., 1H), 5.12 (br. s., 1H), 4.47 (d, J = 6.02 Hz, 2H), 3.46 (q, J = 6.30 Hz, 2H), 2.52 (t, J = 5.73 Hz, 2H), 1.43 (s, 10H) , Molecular Weight: 279.34

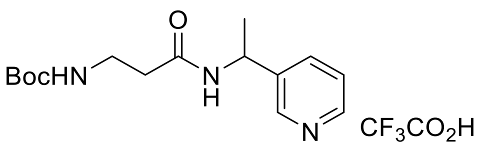

Tert-butyl (3-oxo-3-((1-(pyridin-3-yl)ethyl)amino)propyl)carbamate 2,2,2-trifluoroacetate **22** was synthesized using the **General procedure for amide formation** and BOC-β-alanine and 1-(pyridin-3-yl)ethan-1-amine as the coupling partners. The volatiles were removed in vacuo, and the resulting residue was diluted with acidic (pH=1) water and purified via reverse phase C18 flash chromatography (gradient 0-100% H_2O + 0.05% TFA/MeCN + 0.05% TFA) to provide to provide 0.8412 g pale yellow oily amorphous solid after lyophilization (98% yield). The product was contaminated with tetramethylurea and DMF and used in the next step without further purification. ^{1}H NMR (500 MHz, METHANOL-d4) δ 8.60 (s, 1H), 8.51 (d, J = 4.30 Hz, 1H), 8.01 (d, J = 8.02 Hz, 1H), 7.58 (dd, J = 5.16, 8.02 Hz, 1H), 5.06 (q, J = 7.16 Hz, 1H), 3.26 - 3.30 (m, 2H), 2.34 - 2.48 (m, 2H), 1.50 (d, J = 7.16 Hz, 3H), 1.42 (s, 9H). Molecular Weight of free base: 293.37; Molecular Weight of TFA salt: 407.39.

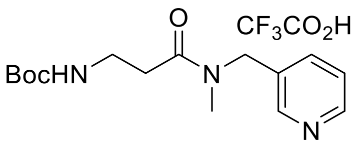

Tert-butyl (3-(methyl(pyridin-3-ylmethyl)amino)-3-oxopropyl)carbamate 2,2,2-trifluoroacetate **23** was synthesized using the **General procedure for amide formation** and BOC-β-alanine and 3-[(methylamino)methyl]pyridine as the coupling partners. The volatiles were removed in vacuo, and the resulting residue was diluted with acidic (pH=1) water and purified via reverse phase C18 flash chromatography (gradient 0-100% H_2O + 0.05% TFA/MeCN + 0.05% TFA) to provide to provide 0.9407 g pale yellow oil after lyophilization (97.5% yield). The product existed as a mixture of rotamers and was contaminated with trace tetramethylurea and used in the next step without further purification. ^1H NMR (500 MHz, METHANOL-d4) δ 8.47 - 8.59 (m, 2H), 7.94 (d, J = 8.02 Hz, 1H), 7.50 - 7.59 (m, 1H), 4.67 (s, 2H), 3.32 - 3.38 (m, 2H), 3.06 (s, 2H), 2.93 (s, 1H), 2.60 - 2.69 (m, 2H), 1.37 - 1.50 (m, 9H). Molecular Weight of free base: 293.37; Molecular Weight of TFA salt: 407.39.

General procedure for Boc deprotection using the synthesis of *3-oxo-3-(pentylamino)propan-1-aminium 2,2,2-trifluoroacetate* **24** as an example:

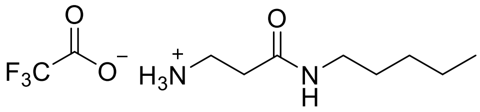

To a solution of *tert*-butyl (3-oxo-3-(pentylamino)propyl)carbamate **13** (1139.0 mg, 4.946 mmol, 1.0 equiv.) in dichloromethane (3.3 ml, ca. 1.5 M) was added triisopropylsilane (101 ul, 0.495 mmol, 0.1 equiv.), water (18 ul, 0.989 mmol, 0.2 equiv.) and TFA (1.89 ml, 24.73 equiv., 5.0 equiv.). The TFA was added over 5 min at rt. The reaction was monitored by LC/MS, and an additional 5 equiv. of TFA was added if the reaction still contained starting material after 8 h at rt. The volatiles were removed in vacuo, and the resulting residue was diluted with water (+0.05% TFA) and purified via reverse phase C18 flash chromatography (gradient 0-100% H_2O + 0.05% TFA/MeCN + 0.05% TFA) to provide 1.3237 g of **24** as a clear colorless oil after lyophilization (98% yield). ^1H NMR (500 MHz, DEUTERIUM OXIDE) δ 3.19 (t, J = 6.87 Hz, 2H), 3.12 (t, J = 7.02 Hz, 2H), 2.59 (t, J = 6.87 Hz, 2H), 1.44 (quin, J = 7.09 Hz, 2H), 1.14 - 1.30 (m, 4H), 0.74 - 0.87 (m, 3H). ^{13}C (CD3OD) δ 172.77, 164.21 (TFA), 163.92 (TFA), 117.48 (q, J C-F = 291.5 Hz), 40.6, 37.0, 33.1, 29.4, 29.0, 22.8, 14.4. Molecular Weight TFA salt: 272.27, Molecular Weight free base: 158.25.

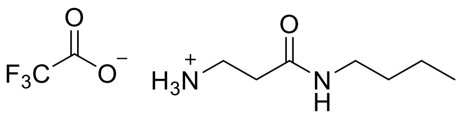

3-(Butylamino)-3-oxopropan-1-aminium 2,2,2-trifluoroacetate **25** was synthesized using the **General procedure for Boc deprotection** to provide 1.265 g of **25** as a clear colorless oil after lyophilization (83% yield). ^{1}H NMR (500 MHz, DEUTERIUM OXIDE) δ 3.19 (t, J = 6.73 Hz, 2H), 3.13 (t, J = 6.87 Hz, 2H), 2.59 (t, J = 6.73 Hz, 2H), 1.36 - 1.48 (m, 2H), 1.19 - 1.30 (m, 2H), 0.83 (t, J = 7.30 Hz, 3H). ^{13}C NMR (126 MHz, METHANOL-d4) δ 172.8, 163.87 (q, J C-F = 35 Hz), 117.54 (q, J C-F = 290 Hz), 40.3, 37.0, 33.1, 31.5, 20.6, 14.1. Molecular Weight TFA salt: 258.24, Molecular Weight free base: 145.23.

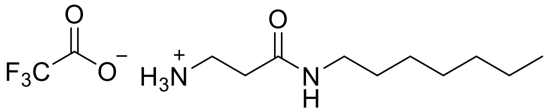

3-(Heptylamino)-3-oxopropan-1-aminium 2,2,2-trifluoroacetate **26** was synthesized using the **General procedure for Boc deprotection** to provide 1.5228 g of **26** as an amorphous white solid after lyophilization (93% yield). ^{1}H NMR (500 MHz, DEUTERIUM OXIDE) δ 3.19 (t, J = 6.87 Hz, 2H), 3.12 (t, J = 7.02 Hz, 2H), 2.59 (t, J = 6.87 Hz, 2H), 1.44 (quin, J = 6.87 Hz, 2H), 1.11 - 1.30 (m, 8H), 0.74 - 0.87 (m, 3H). ^{13}C NMR (126 MHz, METHANOL-d4) δ 172.8, 40.6, 37.0, 33.0, 32.3, 29.4, 29.3, 27.3, 23.2, 14.5. Molecular Weight TFA salt: 300.32, Molecular Weight free base: 186.30.

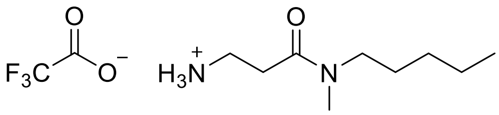

3-(Methyl(pentyl)amino)-3-oxopropan-1-aminium 2,2,2-trifluoroacetate **27** was synthesized using the **General procedure for Boc deprotection** to provide 1.3848 g of **27** as a clear pale-yellow oil after lyophilization (99% yield). ^{1}H NMR (500 MHz, DEUTERIUM OXIDE) δ 3.29 (dt, J = 4.15, 7.38 Hz, 2H), 3.19 (td, J = 3.19, 5.94 Hz, 2H), 2.96 (s, 2H), 2.85 (s, 1H), 2.78 (td, J = 6.41, 17.54 Hz, 2H), 1.42 - 1.60 (m, 2H), 1.12 - 1.32 (m, 4H), 0.82 (q, J = 7.16 Hz, 3H). ^{13}C NMR (126 MHz, METHANOL-d4) d 172.41, 172.38, (peaks from TFA 163.92, 163.64, 118.78, 116.46), 51.03, 36.96, 36.83, 36.08, 34.33, 30.97, 30.43, 29.50, 29.30, 28.12, 27.27, 23.01, 14.41, 14.38. (extra ^{13}C signals due to amide rotamers). Molecular Weight TFA salt: 286.3, Molecular Weight free base: 172.27.

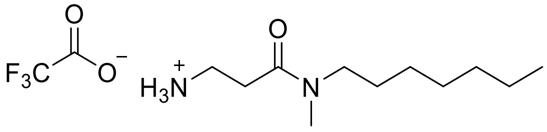

3-(Heptyl(methyl)amino)-3-oxopropan-1-aminium 2,2,2-trifluoroacetate **28** was synthesized using the **General procedure for Boc deprotection** to provide 1.2855 g of **28** as a clear colorless oil after lyophilization (77% yield). ^{1}H NMR (500 MHz, DEUTERIUM OXIDE) δ 3.28 (dt, J = 4.58, 7.45 Hz, 2H), 3.19 (dt, J = 3.01, 6.09 Hz, 2H), 2.95 (s, 2H), 2.85 (s, 1H), 2.79 (t, J = 6.30 Hz, 1H), 2.75 (t, J = 6.44 Hz, 1H), 1.54 (quin, J = 7.45 Hz, 1H), 1.47 (quin, J = 7.38 Hz, 1H), 1.10 - 1.31 (m, 9H), 0.72 - 0.85 (m, 3H). Molecular Weight TFA salt: 314.35, Molecular Weight free base: 200.33.

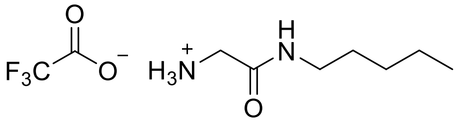

2-Oxo-2-(pentylamino)ethan-1-aminium 2,2,2-trifluoroacetate **29** was synthesized using the **General procedure for Boc deprotection** to provide 0.8795 g of **29** (55% yield). ^{1}H NMR (500 MHz, DEUTERIUM OXIDE) δ 3.68 (s, 2H), 3.15 (t, J = 7.02 Hz, 2H), 1.36 - 1.51 (m, 2H), 1.15 - 1.30 (m, 4H), 0.78 (dt, J = 1.29, 7.09 Hz, 3H). Molecular Weight TFA salt: 258.24, Molecular Weight free base: 144.22.

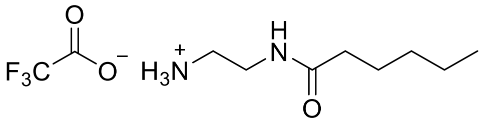

2-Hexanamidoethan-1-aminium 2,2,2-trifluoroacetate **36** was synthesized using the **General procedure for Boc deprotection** to provide 0.6455 g of **36** as a white amorphous solid after lyophilization (83% yield). ^{1}H NMR (500 MHz, DEUTERIUM OXIDE) δ 3.38 (t, J = 6.02 Hz, 2H), 3.03 (t, J = 5.87 Hz, 2H), 2.15 (t, J = 7.59 Hz, 2H), 1.47 (quin, J = 7.38 Hz, 2H), 1.07 - 1.29 (m, 4H), 0.75 (t, J = 6.87 Hz, 3H)Molecular Weight TFA salt: 314.35, Molecular Weight free base: 200.33.

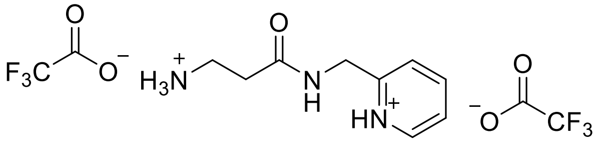

2-((3-Ammoniopropanamido)methyl)pyridin-1-ium 2,2,2-trifluoroacetate **30** was synthesized using the **General procedure for Boc deprotection** to provide 2.394 g of **30** as a cloudy colorless oil after lyophilization (102% yield). ^{1}H NMR (500 MHz, DEUTERIUM OXIDE) d 8.58 - 8.63 (m, 1H), 8.48 (dt, J = 1.58, 7.95 Hz, 1H), 7.86 - 7.92 (m, 2H), 4.71 (s, 2H), 3.20 (t, J = 6.59 Hz, 2H), 2.75 (t, J = 6.59 Hz, 2H). ^{13}C NMR (126 MHz, METHANOL-d4) d 174.3, 163.7 (TFA),

163.4 (TFA), 153.7, 148.5, 142.0, 127.0, 126.9, 117.29 (q, J C-F = 291.5 Hz), 41.7, 36.5, 32.7. Molecular Weight bis TFA salt: 407.27, Molecular Weight free base: 179.22.

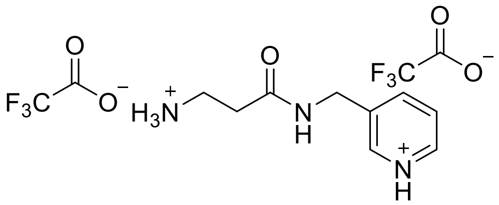

3-((3-Ammoniopropanamido)methyl)pyridin-1-ium 2,2,2-trifluoroacetate **31** was synthesized using the **General procedure for Boc deprotection** to provide 2.3155 g of **31** as a cloudy colorless oil after lyophilization (105% yield). ^{1}H NMR (500 MHz, DEUTERIUM OXIDE) δ 8.62 - 8.67 (m, 2H), 8.45 - 8.49 (m, 1H), 7.98 (dd, J = 6.02, 8.02 Hz, 1H), 4.55 (s, 2H), 3.20 (t, J = 6.73 Hz, 2H), 2.71 (t, J = 6.73 Hz, 2H). ^{13}C NMR (126 MHz, METHANOL-d4) δ□173.8, 163.72 (q, J C-F = 36 Hz), 147.0, 141.1, 140.9, 140.0, 128.4, 116.17 (q, J C-F = 291.3 Hz), 41.0, 36.6, 32.8. Molecular Weight bis TFA salt: 407.27, Molecular Weight free base: 179.22.

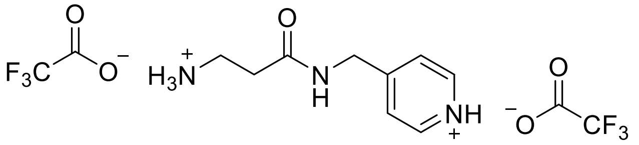

4-((3-Ammoniopropanamido)methyl)pyridin-1-ium 2,2,2-trifluoroacetate **32** was synthesized using the **General procedure for Boc deprotection** to provide 2.3097 g of **32** as a cloudy colorless oil after lyophilization (99% yield). ^{1}H NMR (500 MHz, DEUTERIUM OXIDE) δ 8.58 - 8.67 (m, 2H), 7.82 - 7.93 (m, 2H), 4.62 (s, 2H), 3.21 (t, J = 6.59 Hz, 2H), 2.76 (t, J = 6.73 Hz, 2H). ^{13}C NMR (126 MHz, METHANOL-d4) δ 174.0, 163.78 (q, J C-F = 36 Hz), 161.2, 142.0, 126.1, 117.32 (q, J C-F = 291.3 Hz), 43.6, 36.6, 32.8. Molecular Weight bis TFA salt: 407.27, Molecular Weight free base: 179.22.

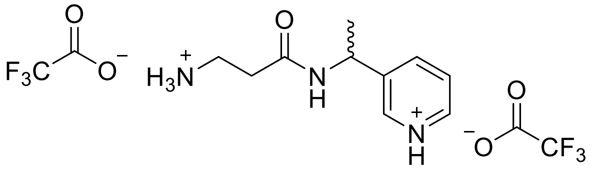

3-(1-(3-Ammoniopropanamido)ethyl)pyridin-1-ium 2,2,2-trifluoroacetate **33** was synthesized using the **General procedure for Boc deprotection** to provide 0.7139 g of **33** as a clear colorless oil after lyophilization (82% yield). ^{1}H NMR (500 MHz, DEUTERIUM OXIDE) δ 8.68 - 8.75 (m, 1H), 8.64 (d, J = 5.73 Hz, 1H), 8.53 (d, J = 8.31 Hz, 1H), 8.00 (dd, J = 5.87, 8.16 Hz, 1H), 5.08 (q, J = 7.16 Hz, 1H), 3.17 (t, J = 6.73 Hz, 2H), 2.58 - 2.78 (m, 2H), 1.49 (d, J = 7.16 Hz, 3H). ^{13}C NMR (126 MHz, METHANOL-d4) d 172.9, 164.02 (q, J C-F = 36 Hz), 145.7, 141.1, 140.2, 128.6, 116.28 (q, J C-F = 291.5 Hz), 48.2, 36.5, 32.7, 21.3. Molecular Weight bis TFA salt: 421.30, Molecular Weight free base: 193.25.

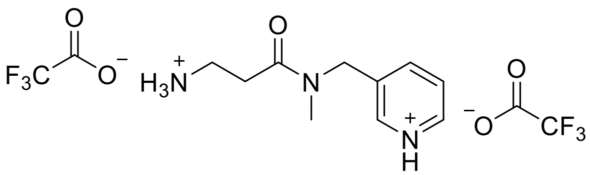

3-((3-Ammonio-N-methylpropanamido)methyl)pyridin-1-ium 2,2,2-trifluoroacetate **34** was synthesized using the **General procedure for Boc deprotection** to provide 0.9407 g of **34** as a clear yellow oil after lyophilization (97% yield). ^{1}H NMR (500 MHz, DEUTERIUM OXIDE) δ 8.63 (d, J = 5.44 Hz, 0.12 H), 8.53 - 8.60 (m, 1.67H), 8.33 - 8.41 (m, 1H), 7.96 (dd, J = 6.02, 8.02 Hz, 0.14H), 7.91 (dd, J = 6.02, 8.02 Hz, 0.86H), 4.76 (s, 0.35H), 4.66 (s, 1.82H), 3.14 (t, J = 6.30 Hz, 2H), 2.98 (s, 3H), 2.78 - 2.84 (m, 2.37H), 2.75 (t, J = 6.16 Hz, 0.33H). Partial proton integration reflects amide rotamers. Molecular Weight bis TFA salt: 421.30, Molecular Weight free base: 193.25.

General procedure for Pantothenamide formation using the synthesis of *(R)-2,4-dihydroxy-3,3-dimethyl-N-(3-oxo-3-(pentylamino)propyl)butanamide* **1** as an example:

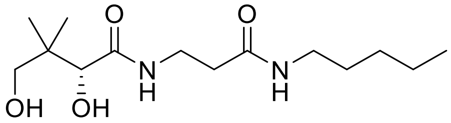

To a solution of 3-oxo-3-(pentylamino)propan-1-aminium 2,2,2-trifluoroacetate **24** (1323.7 mg, 4.862 mmol, 1.0 equiv.) in isopropanol (2.4 ml, ca. 2 M) and *N,N*-diisopropylethylamine (2.12 ml, 12.15 mmol, 2.5 equiv.) was added (R)-pantolactone (949 mg, 7.293 mmol, 1.5 equiv.). The reaction was heated to 82°C in a sealed vial for 18 h. The organic volatiles were removed in vacuo, and the resulting residue was diluted with water and purified via reverse phase C18 flash chromatography (gradient 0-100% H_2O/MeCN) to provide 1.2563 g of **1** as a white amorphous solid after lyophilization (89% yield). ^{1}H NMR (500 MHz, DEUTERIUM OXIDE) δ 3.91 (s, 1H), 3.36 - 3.55 (m, 3H), 3.32 (d, J = 11.17 Hz, 1H), 3.01 - 3.16 (m, 2H), 2.41 (t, J = 6.44 Hz, 2H), 1.43 (quin, J = 7.16 Hz, 2H), 1.13 - 1.31 (m, 4H), 0.75 - 0.88 (m, 10H). ^{13}C NMR (126 MHz, METHANOL-d4) δ 176.2, 174.6, 76.9, 69.5, 40.6, 39.7, 36.6, 36.4, 29.5, 29.1, 22.8, 21.6, 20.2, 14.4. HRMS (ESI+): m/z $[M + Na]^+$ calculated for $C_{14}H_{28}N_2O_4Na$ = 311.1947, found: 311.1941.

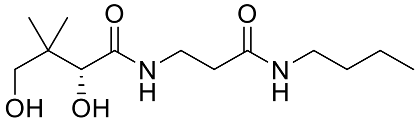

(R)-N-(3-(Butylamino)-3-oxopropyl)-2,4-dihydroxy-3,3-dimethylbutanamide **2** was synthesized using the **General procedure for Pantothenamide formation** except that starting material still remained after 18h of heating. To push the reaction to completion, an additional 1.5 eq. of (R)-pantolactone (3 equiv. total) was added and heated an additional 24 h to provide 0.9429 g of **2** as a pale yellow oil after lyophilization (90% yield). ^{1}H NMR (500 MHz, DEUTERIUM OXIDE) δ 3.91 (s, 1H), 3.36 - 3.52 (m, 3H), 3.32 (d, J = 11.17 Hz, 1H), 3.01 - 3.16 (m, 2H), 2.41 (t, J = 6.44 Hz, 2H), 1.33 - 1.47 (m, 2H), 1.17 - 1.32 (m, 2H), 0.76 - 0.88 (m, 9H). ^{13}C NMR (126 MHz,

METHANOL-d4 in DEUTERIUM OXIDE) δ 176.2, 174.6, 76.9, 69.5, 40.4, 39.7, 36.6, 36.4, 31.5, 21.6, 20.6, 20.2, 14.1. HRMS (ESI+): m/z $[M + Na]^+$ calculated for $C_{13}H_{26}N_2O_4Na$ = 297.1790, found: 297.1787.

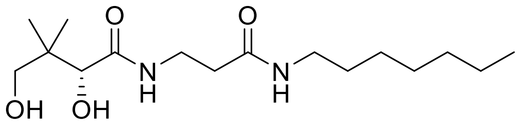

(R)-N-(3-(Heptylamino)-3-oxopropyl)-2,4-dihydroxy-3,3-dimethylbutanamide **3** was synthesized using the **General procedure for Pantothenamide formation** to provide 0.9612 g of **3** as a white amorphous solid after lyophilization (63% yield). ^{1}H NMR (500 MHz, DEUTERIUM OXIDE) δ 3.91 (s, 1H), 3.37 - 3.52 (m, 3H), 3.32 (d, J = 11.17 Hz, 1H), 3.02 - 3.15 (m, 2H), 2.41 (t, J = 6.44 Hz, 2H), 1.37 - 1.47 (m, 2H), 1.17 - 1.27 (m, 8H), 0.78 - 0.86 (m, 9H). ^{13}C NMR (126 MHz, METHANOL-d4) δ 176.2, 173.7, 77.4, 70.5, 40.6, 40.5, 36.6, 36.6, 33.1, 30.5, 30.3, 28.2, 23.8, 21.4, 21.0, 14.6. HRMS (ESI+): m/z $[M + Na]^+$ calculated for $C_{16}H_{32}N_2O_4Na$ = 339.2260, found: 339.2260.

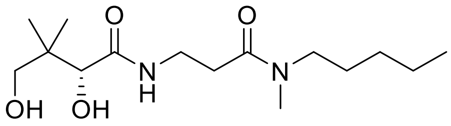

(R)-2,4-Dihydroxy-3,3-dimethyl-N-(3-(methyl(pentyl)amino)-3-oxopropyl)butanamide **4** was synthesized using the **General procedure for Pantothenamide formation** to provide 1.3028 g of **4** as a pale yellow oil after lyophilization (91% yield). ^{1}H NMR (500 MHz, DEUTERIUM OXIDE) δ 3.91 (d, J = 1.15 Hz, 1H), 3.36 - 3.52 (m, 3H), 3.19 - 3.36 (m, 3H), 2.98 (s, 1.5H, methyl amide rotamer), 2.84 (s, 1.5H, methyl amide rotamer), 2.62 (td, J = 6.59, 13.75 Hz, 2H), 1.54 (quin, J = 7.52 Hz, 1H), 1.46 (quin, J = 7.52 Hz, 1H), 1.12 - 1.31 (m, 4H), 0.75 - 0.88 (m, 9H). ^{13}C NMR (126 MHz, METHANOL-d4) δ 176.1, 176.1, 173.2, 77.3, 70.5, 51.1, 40.5, 36.2, 36.0, 35.9, 34.2, 33.9, 33.7, 30.2, 30.0, 29.1, 28.0, 23.6, 23.6, 21.4, 21.0, 21.0, 14.5, 14.5 (extra ^{13}C signals due to amide rotamers). HRMS (ESI+): m/z $[M + Na]^+$ calculated for $C_{15}H_{30}N_2O_4Na$ = 325.2103, found: 325.2100.

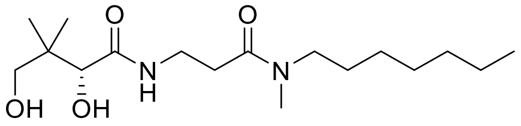

(R)-N-(3-(heptyl(methyl)amino)-3-oxopropyl)-2,4-dihydroxy-3,3-dimethylbutanamide **5** was synthesized using the **General procedure for Pantothenamide formation** to provide an oil after lyophilization that was insufficiently pure. Additional purification (silica gel, dichloromethane/MeOH) provided 0.9464 g of **5** as a clear colorless oil (73% yield). ^{1}H NMR (500 MHz, METHANOL-d4) δ 3.86 (s, 1H), 3.41 - 3.52 (m, 3H), 3.26 - 3.39 (m, 3H), 2.99 (s, 1.6H, methyl amide rotamer), 2.87 (s, 1.3H, methyl amide rotamer), 2.49 - 2.65 (m, 2H), 1.43 - 1.61 (m, 2H), 1.18 - 1.40 (m, 8H), 0.79 - 0.95 (m, 9H). ^{13}C NMR (126 MHz, METHANOL-d4) δ

176.0, 176.0, 173.2, 77.3, 70.5, 70.5, 51.1, 48.9, 40.5, 36.2, 36.0, 35.9, 34.2, 33.9, 33.7, 33.1, 33.0, 30.3, 30.3, 29.4, 28.3, 28.0, 27.8, 23.8, 23.8, 21.4, 21.1, 21.0, 14.6. (extra ^{13}C signals due to amide rotamers). HRMS (ESI+): m/z $[M + Na]^+$ calculated for $C_{17}H_{34}N_2O_4Na$ = 353.2416, found: 353.2411.

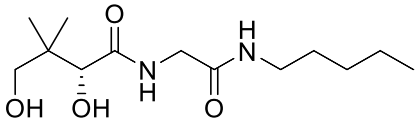

(R)-2,4-Dihydroxy-3,3-dimethyl-N-(2-oxo-2-(pentylamino)ethyl)butanamide **6** was synthesized using the **General procedure for Pantothenamide formation** to provide 0.7589 g of **6** as a clear colorless oil after lyophilization (45% yield). ^1H NMR (500 MHz, DEUTERIUM OXIDE) δ 3.97 (s, 1H), 3.84 (s, 2H), 3.34 - 3.51 (m, 2H), 3.14 (t, J = 7.02 Hz, 2H), 1.43 (quin, J = 7.09 Hz, 2H), 1.14 - 1.32 (m, 4H), 0.89 (d, J = 4.58 Hz, 6H), 0.81 (t, J = 7.02 Hz, 3H). ^{13}C NMR (126 MHz, METHANOL-d4) δ 176.7, 171.6, 77.6, 69.6, 43.1, 40.8, 40.5, 30.3, 30.2, 23.5, 22.2, 21.4, 14.5. HRMS (ESI+): m/z $[M + Na]^+$ calculated for $C_{13}H_{26}N_2O_4Na$ = 297.1790, found: 297.1788.

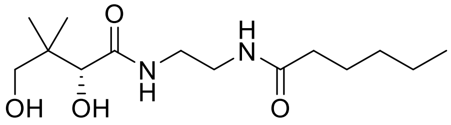

(R)-N-(2-(2,4-Dihydroxy-3,3-dimethylbutanamido)ethyl)hexanamide **7** was synthesized using the **General procedure for Pantothenamide formation** to provide 0.5073 g of **7** as a white amorphous solid after lyophilization (74% yield). ^1H NMR (500 MHz, DEUTERIUM OXIDE) δ 3.93 (s, 1H), 3.45 (d, J = 11.17 Hz, 1H), 3.22 - 3.41 (m, 5H), 2.16 (t, J = 7.45 Hz, 2H), 1.51 (quin, J = 7.38 Hz, 2H), 1.14 - 1.30 (m, 4H), 0.78 - 0.88 (m, 9H). ^{13}C NMR (126 MHz, METHANOL-d4) δ 176.9, 176.7, 77.5, 70.4, 40.5, 40.2, 39.8, 37.3, 32.7, 26.8, 23.6, 21.6, 21.1, 14.4. HRMS (ESI+): m/z $[M + Na]^+$ calculated for $C_{14}H_{28}N_2O_4Na$ = 311.1947, found: 311.1942.

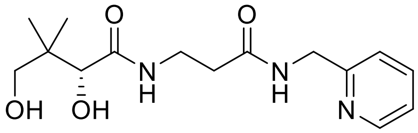

(R)-2,4-Dihydroxy-3,3-dimethyl-N-(3-oxo-3-((pyridin-2-ylmethyl)amino)propyl)butanamide **8** was synthesized using the **General procedure for Pantothenamide formation** to provide 0.1345 g of **8** as a clear colorless oil after lyophilization (7.7% yield). ^1H NMR (500 MHz, METHANOL-d4) δ 8.44 - 8.50 (m, 1H), 7.80 (dt, J = 1.72, 7.73 Hz, 1H), 7.38 (d, J = 7.73 Hz, 1H), 7.27 - 7.33 (m, 1H), 4.49 (s, 2H), 3.90 (s, 1H), 3.48 - 3.59 (m, 2H), 3.46 (d, J = 11.17 Hz, 1H), 3.39 (d, J = 10.88 Hz, 1H), 2.54 (t, J = 6.59 Hz, 2H), 0.91 (s, 6H). ^{13}C NMR (126 MHz, METHANOL-d4) δ 176.2, 174.1, 159.2, 149.9, 139.0, 123.9, 123.2, 77.4, 70.4, 45.6, 40.5, 36.5, 36.5, 21.5, 21.0. HRMS (ESI+): m/z $[M + Na]^+$ calculated for $C_{15}H_{23}N_3O_4Na$ = 332.1586, found: 332.1583.

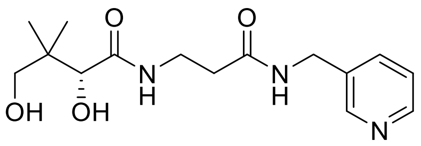

(R)-2,4-Dihydroxy-3,3-dimethyl-N-(3-oxo-3-((pyridin-3-ylmethyl)amino)propyl)butanamide **9** was synthesized using the **General procedure for Pantothenamide formation** to provide 0.4981 g of **9** as a clear colorless oil after lyophilization (29.8 % yield). ^{1}H NMR (500 MHz, METHANOL-d4) δ 8.46 - 8.52 (m, 1H), 8.43 (dd, J = 1.29, 4.73 Hz, 1H), 7.74 - 7.82 (m, 1H), 7.35 - 7.47 (m, 1H), 4.41 (s, 2H), 3.89 (s, 1H), 3.41 - 3.57 (m, 3H), 3.34 - 3.41 (m, 1H), 2.49 (t, J = 6.73 Hz, 2H), 0.89 (s, 6H). ^{13}C NMR (126 MHz, METHANOL-d4) δ 176.2, 173.9, 149.6, 149.0, 137.8, 136.7, 125.4, 77.3, 70.4, 41.7, 40.5, 36.5, 36.4, 21.4, 21.0. HRMS (ESI+): m/z $[M + H]^+$ calculated for $C_{15}H_{24}N_3O_4$ = 310.1767, found: 310.1763.

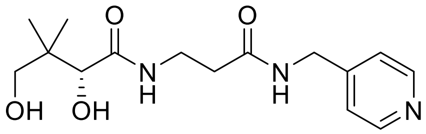

(R)-2,4-Dihydroxy-3,3-dimethyl-N-(3-oxo-3-((pyridin-4-ylmethyl)amino)propyl)butanamide **10** was synthesized using the **General procedure for Pantothenamide formation** to provide 0.234 g of **10** as a clear colorless oil after lyophilization (13.9 % yield). ^{1}H NMR (500 MHz, METHANOL-d4) δ 8.42 - 8.50 (m, 2H), 7.31 - 7.40 (m, 2H), 4.42 (s, 2H), 3.90 (s, 1H), 3.43 - 3.62 (m, 3H), 3.34 - 3.43 (m, 1H), 2.54 (t, J = 6.59 Hz, 2H), 0.84 - 0.96 (m, 6H). ^{13}C NMR (126 MHz, METHANOL-d4) δ 176.2, 174.1, 150.9, 150.2, 124.0, 77.3, 70.4, 43.1, 40.5, 36.5, 36.4, 21.5, 21.0. HRMS (ESI+): m/z $[M + H]^+$ calculated for $C_{15}H_{24}N_3O_4$ = 310.1767, found: 310.1773.

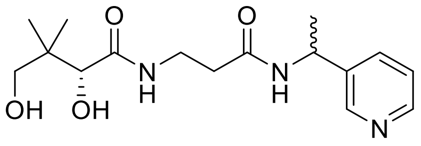

(2R)-2,4-Dihydroxy-3,3-dimethyl-N-(3-oxo-3-((1-(pyridin-3-yl)ethyl)amino)propyl)butanamide **11** was synthesized using the **General procedure for Pantothenamide formation** to provide 0.2639 g of **11** as a clear colorless oil after lyophilization (53.7 % yield). ^{1}H NMR (500 MHz, METHANOL-d4) δ 8.52 (s, 1H), 8.42 (dd, J = 1.43, 4.87 Hz, 1H), 7.81 (dd, J = 1.58, 7.88 Hz, 1H), 7.40 (dd, J = 4.87, 8.02 Hz, 1H), 5.04 (q, J = 6.87 Hz, 1H), 3.89 (d, J = 4.58 Hz, 1H), 3.34 - 3.55 (m, 4H), 2.38 - 2.57 (m, 2H), 1.47 (d, J = 7.16 Hz, 3H), 0.81 - 0.97 (m, 6H). ^{13}C NMR (126 MHz, METHANOL-d4) δ 176.17, 176.13, 173.07, 173.04, 148.77, 148.51, 148.47, 141.76, 136.16, 136.15, 125.39, 77.28, 77.26, 70.43, 48.21, 48.20, 40.51, 40.48, 36.46, 36.45, 36.38, 36.34, 22.15, 21.47, 21.44, 21.03, 20.99 (mixture of diastereomers). HRMS (ESI+): m/z $[M + H]^+$ calculated for $C_{16}H_{26}N_3O_4$ = 324.1923, found: 324.1923.

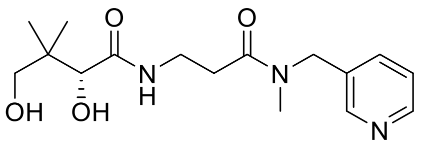

(R)-2,4-Dihydroxy-3,3-dimethyl-N-(3-(methyl(pyridin-3-ylmethyl)amino)-3-oxopropyl)butanamide **12** was synthesized using the **General procedure for Pantothenamide formation** to provide 0.2152 g of **12** as a clear colorless oil after lyophilization (35.7 % yield). Partial proton integration reflects amide rotamers. ^{1}H NMR (500 MHz, METHANOL-d4) δ 8.37 - 8.50 (m, 2H), 7.71 - 7.76 (m, 0.77H), 7.67 - 7.71 (m, 0.24H), 7.41 - 7.47 (m, 0.23H), 7.39 (ddd, J = 0.72, 4.87, 7.88 Hz, 0.78H), 4.55 - 4.70 (m, 2H), 3.87 (s, 1H), 3.41 - 3.58 (m, 3H), 3.32 - 3.40 (m, 1H), 3.00 (s, 2.27H), 2.91 (s, 0.71H), 2.67 (t, J = 6.44 Hz, 2H), 0.84 - 0.93 (m, 6H). ^{13}C NMR (126 MHz, METHANOL-d4) δ 176.1, 174.0, 173.9, 149.8, 149.6, 149.2, 149.0, 138.0, 136.9, 135.4, 134.9, 125.7, 125.5, 77.3, 70.5, 51.8, 49.6, 40.6, 40.5, 36.1, 36.0, 35.8, 34.4, 34.1, 34.0, 21.4, 21.1. (extra ^{13}C signals due to amide rotamers). HRMS (ESI+): m/z $[M + H]^+$ calculated for $C_{16}H_{26}N_3O_4$ = 324.1923, found: 324.1921.

^{1}H NMR of **1**

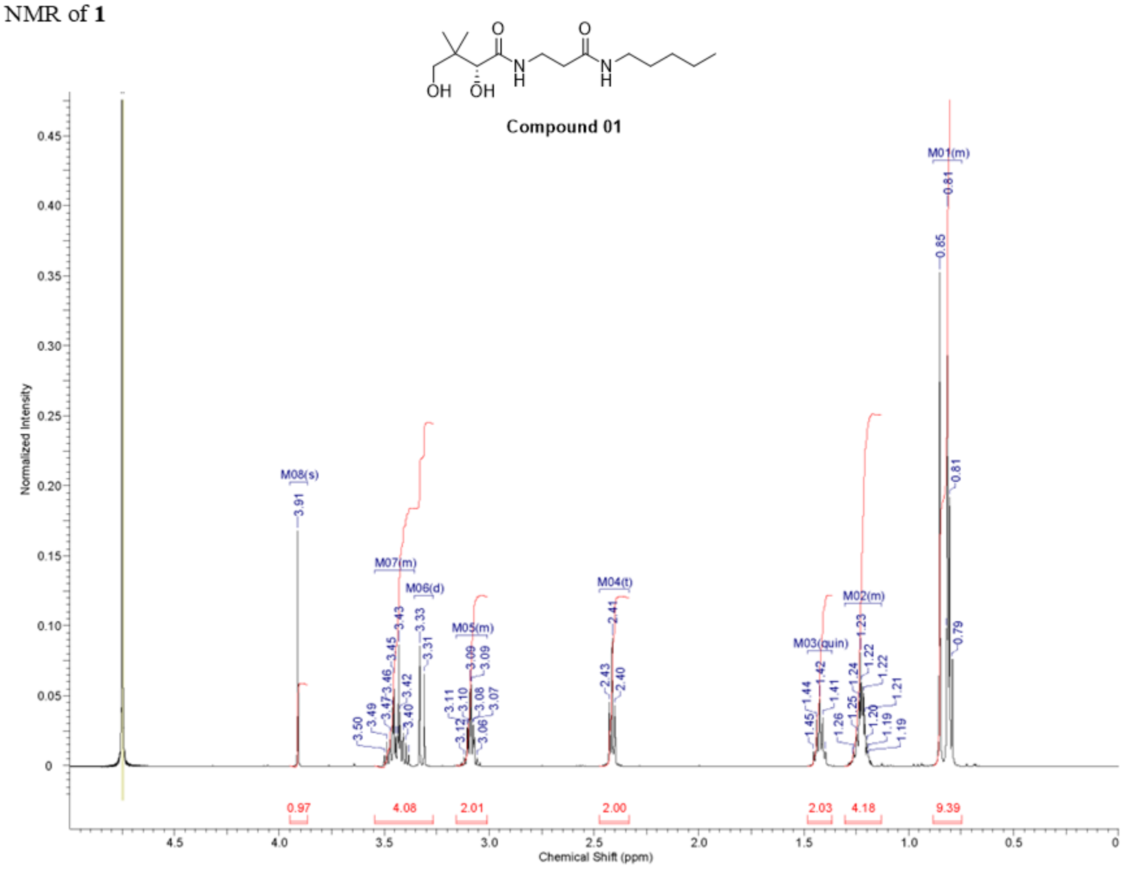

Compound 01
OH
OH
O
O
N
H
N
H
M08(s)
3.91
M07(m)
M06(d)
M05(m)
M04(t)
M03(quin)
M02(m)
M01(m)
0.97
4.08
2.01
2.00
2.03
4.18
9.39
Normalized Intensity
Chemical Shift (ppm)

^{13}C NMR of **1**

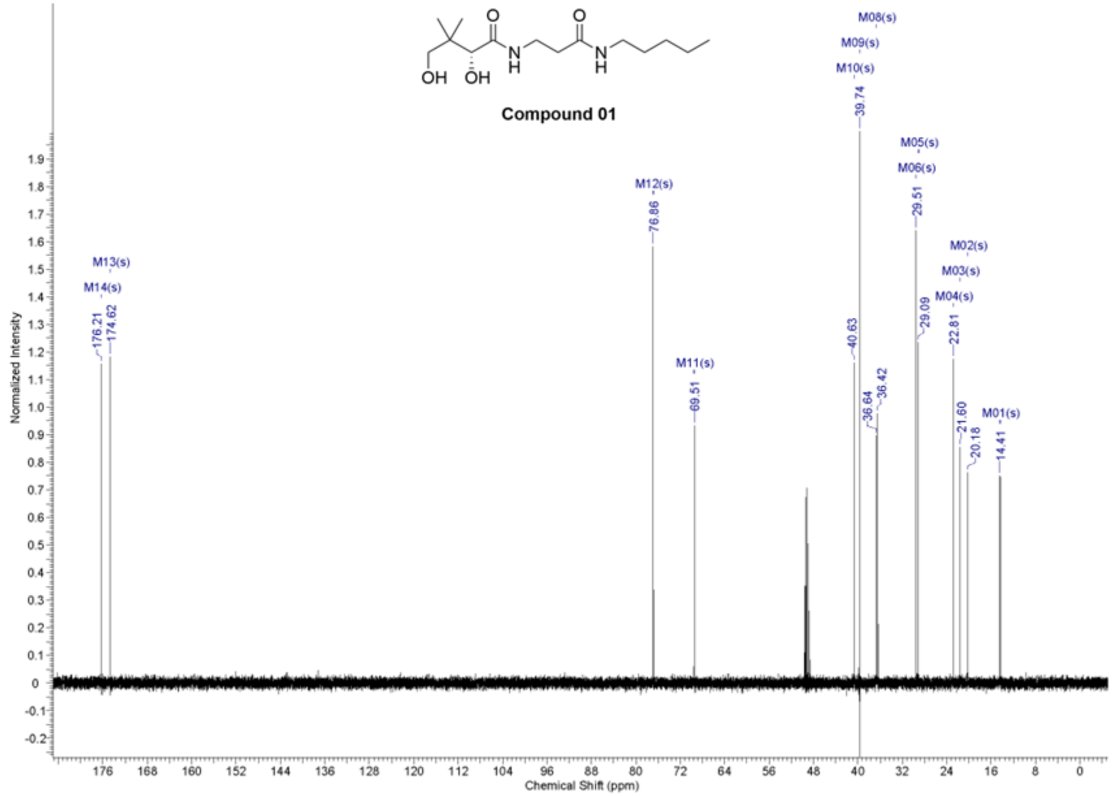
Compound 01
M01(s)
M02(s)
M03(s)
M04(s)
M05(s)
M06(s)
M08(s)
M09(s)
M10(s)
M11(s)
M12(s)
M13(s)
M14(s)
176.21
174.62
76.86
69.51
40.63
39.74
36.64
36.42
29.51
29.09
22.81
21.60
20.18
14.41
Normalized Intensity
Chemical Shift (ppm)

HRMS of **1**

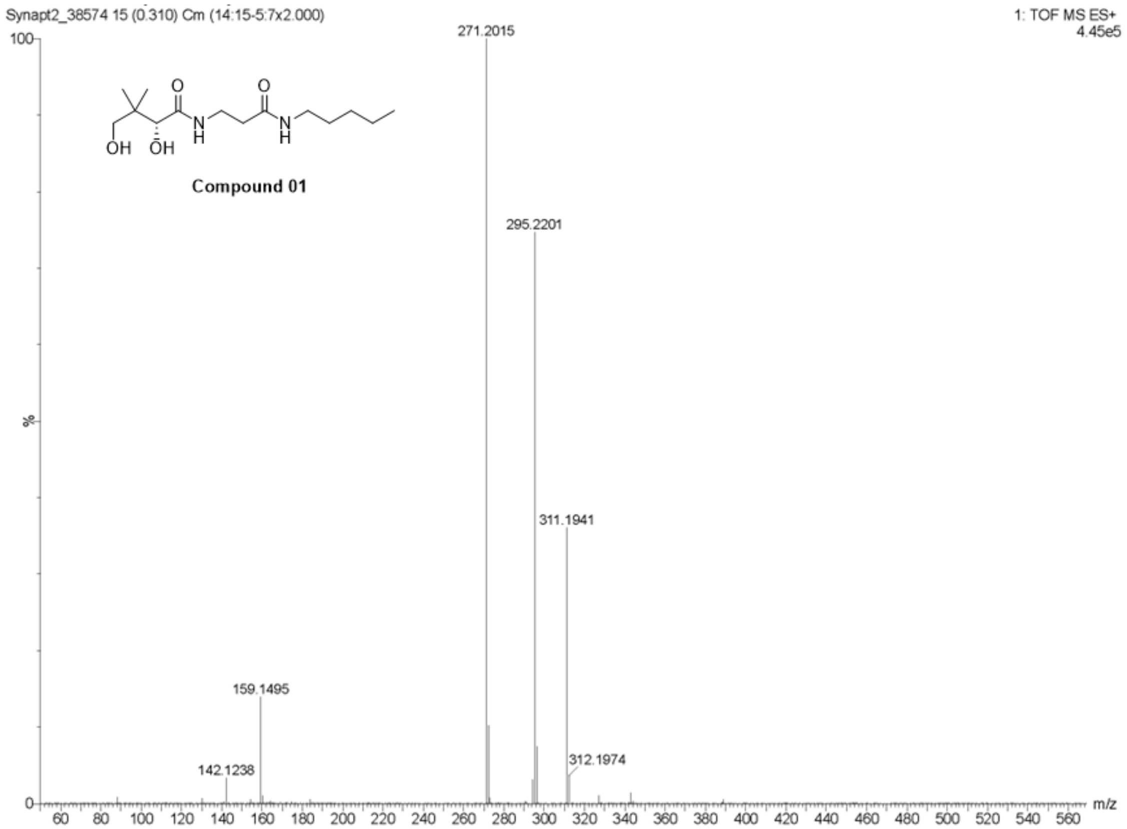

Synapt2_38574 15 (0.310) Cm (14:15-5:7x2.000)
1: TOF MS ES+
4.45e5
Compound 01
271.2015
295.2201
311.1941
159.1495
142.1238
312.1974
%
m/z

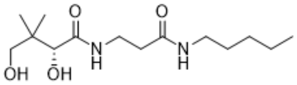

Compound 01

Elemental Composition Report

Single Mass Analysis

Tolerance = 5.0 PPM / DBE: min = -1.5, max = 50.0
Element prediction: Off
Number of isotope peaks used for i-FIT = 3

Monoisotopic Mass, Even Electron Ions
348 formula(e) evaluated with 2 results within limits (up to 50 best isotopic matches for each mass)
Elements Used:
C: 0-50 H: 0-80 N: 0-5 O: 0-7 Na: 0-1

Order# 22608 Meyers Lab djm-14-25-01
Synapt2_38574 15 (0.310) Cm (14:15-5:7x2.000)

MSL, SCS, UIUC

SYNAPTG2-Si#UGA354
1: TOF MS ES+
1.61e+005

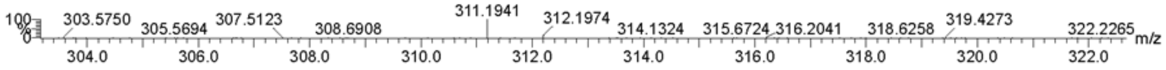

Minimum:				-1.5				
Maximum:		5.0	5.0	50.0				
Mass	Calc. Mass	mDa	PPM	DBE	i-FIT	Norm	Conf(%)	Formula
311.1941	311.1947	-0.6	-1.9	1.5	73.2	0.122	88.54	C14 H28 N2 O4 Na
	311.1931	1.0	3.2	0.5	75.2	2.166	11.46	C11 H27 N4 O6

^{1}H NMR of **2**

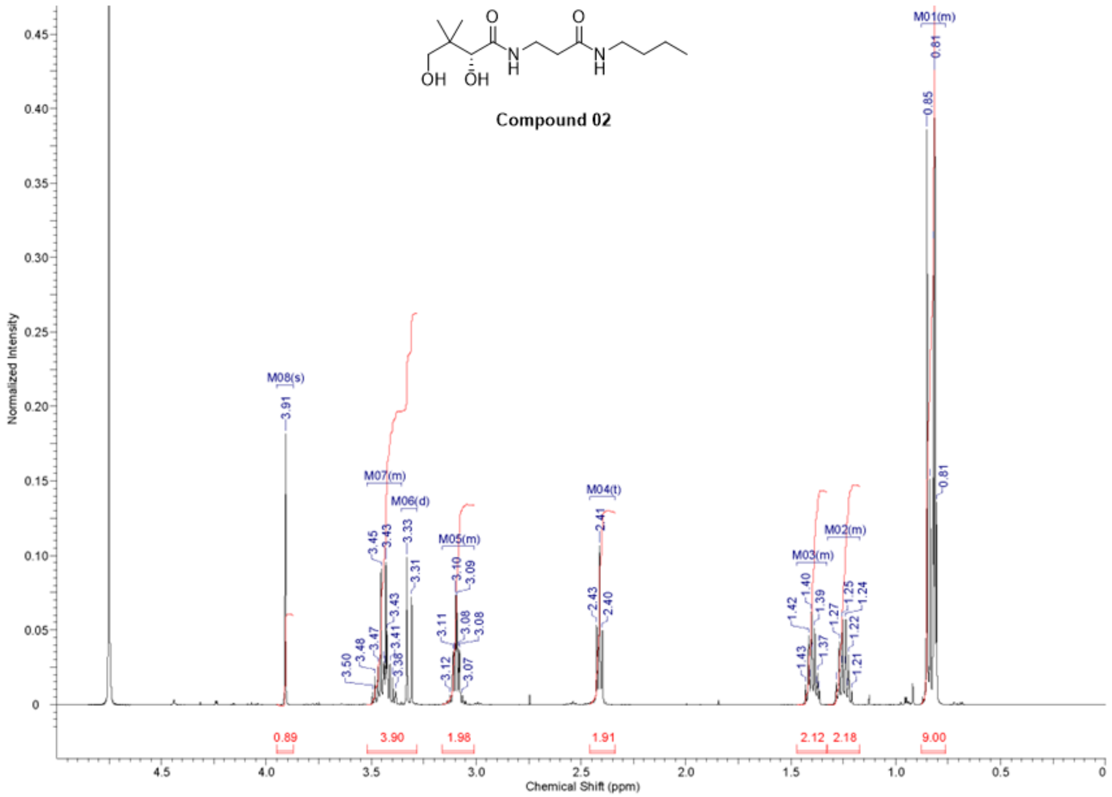
Compound 02
Normalized Intensity
Chemical Shift (ppm)
M08(s)
M07(m)
M06(d)
M05(m)
M04(t)
M03(m)
M02(m)
M01(m)
0.89
3.90
1.98
1.91
2.12
2.18
9.00

^{13}C NMR of **2**

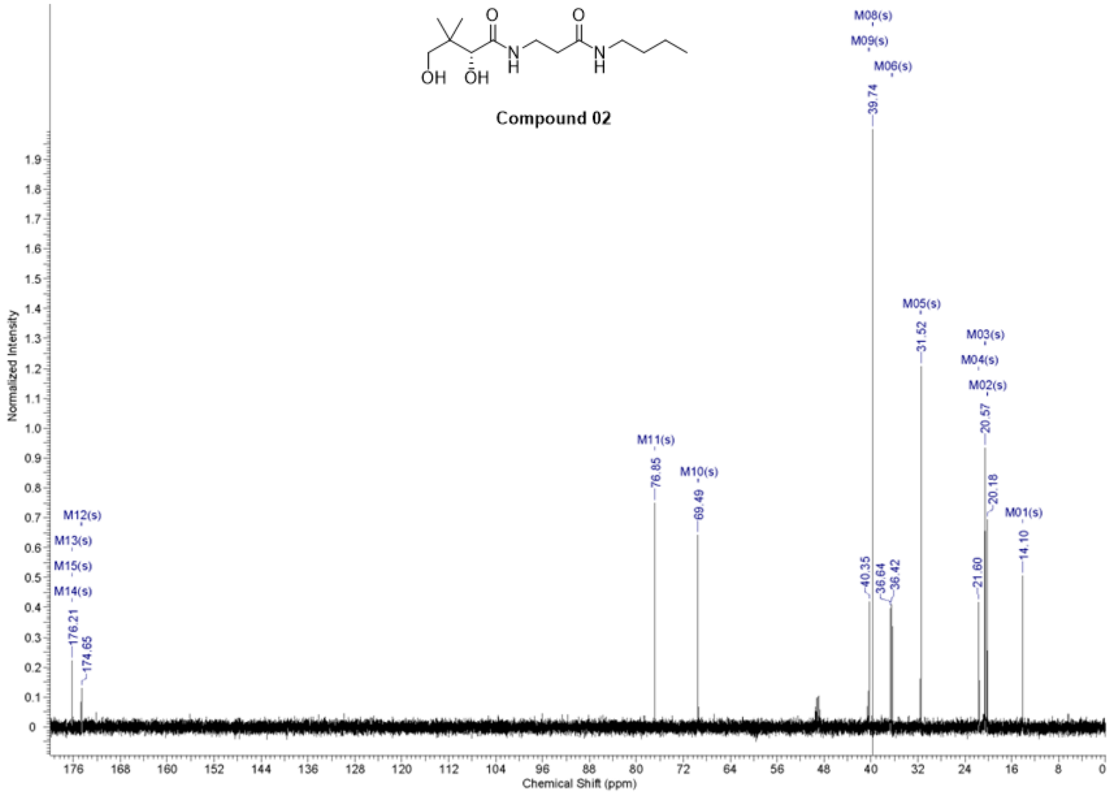

Compound 02
M08(s)
M09(s)
M06(s)
39.74
M05(s)
31.52
M03(s)
M04(s)
M02(s)
20.57
20.18
M01(s)
14.10
21.60
40.35
36.64
36.42
M11(s)
76.85
M10(s)
69.49
M12(s)
M13(s)
M15(s)
M14(s)
176.21
174.65
Normalized Intensity
Chemical Shift (ppm)

HRMS of **2**

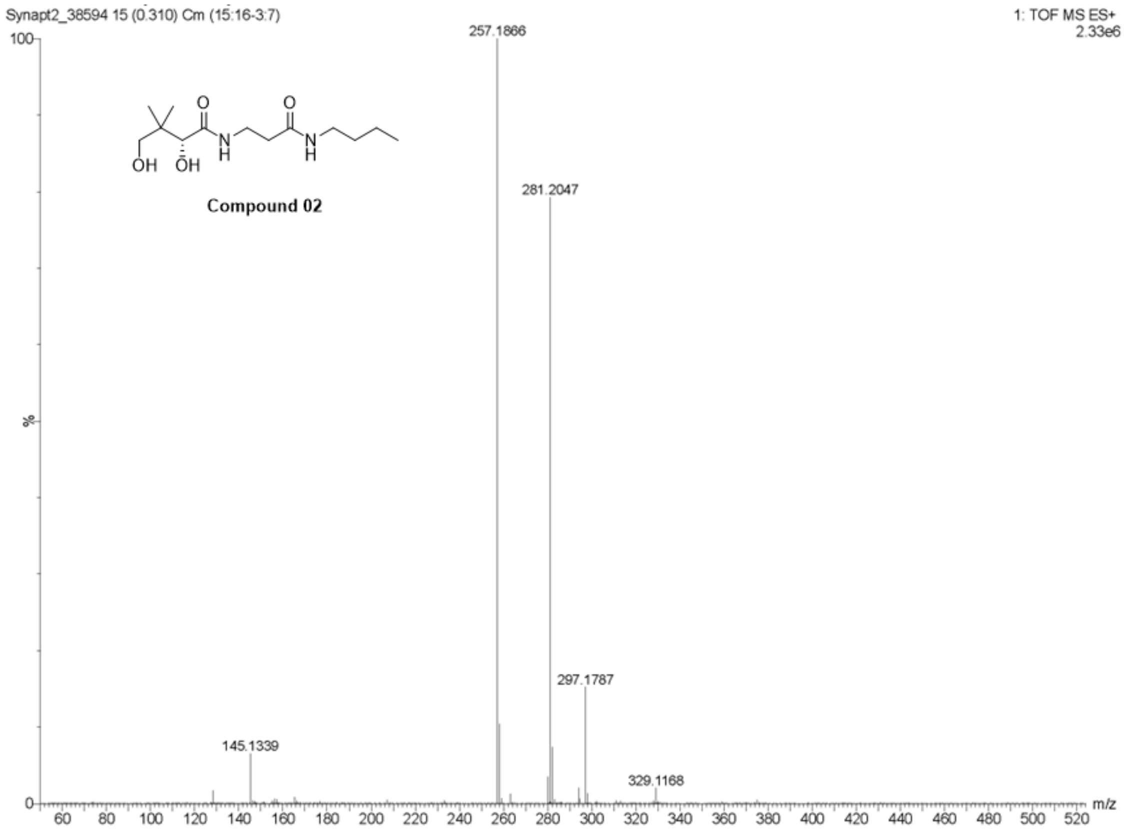

Synapt2_38594 15 (0.310) Cm (15:16-3:7)
1: TOF MS ES+
2.33e6
100
%
0
257.1866
281.2047
297.1787
145.1339
329.1168
Compound 02
OH
OH
N
H
O
m/z

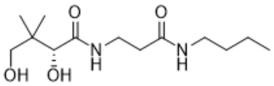

Elemental Composition Report **Compound 02** **Page 1**

Single Mass Analysis
Tolerance = 5.0 PPM / DBE: min = -1.5, max = 50.0
Element prediction: Off
Number of isotope peaks used for i-FIT = 3

Monoisotopic Mass, Even Electron Ions
332 formula(e) evaluated with 2 results within limits (up to 50 best isotopic matches for each mass)
Elements Used:
C: 0-50 H: 0-80 N: 0-5 O: 0-7 Na: 0-1
Order# 22635 Meyers Lab djm-14-25J-01 MSL, SCS, UIUC SYNAPTG2-Si#UGA354
Synapt2_38594 15 (0.310) Cm (15:16-3:7) 1: TOF MS ES+
3.55e+005

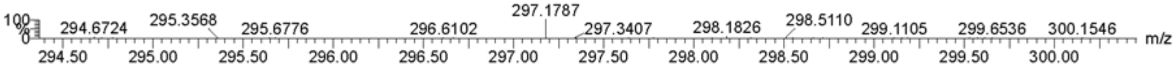

Minimum:				-1.5				
Maximum:		5.0	5.0	50.0				
Mass	Calc. Mass	mDa	PPM	DBE	i-FIT	Norm	Conf(%)	Formula
297.1787	297.1790	-0.3	-1.0	1.5	153.6	0.053	94.88	C13 H26 N2 O4 Na
	297.1774	1.3	4.4	0.5	156.5	2.971	5.12	C10 H25 N4 O6

^{1}H NMR of **3**

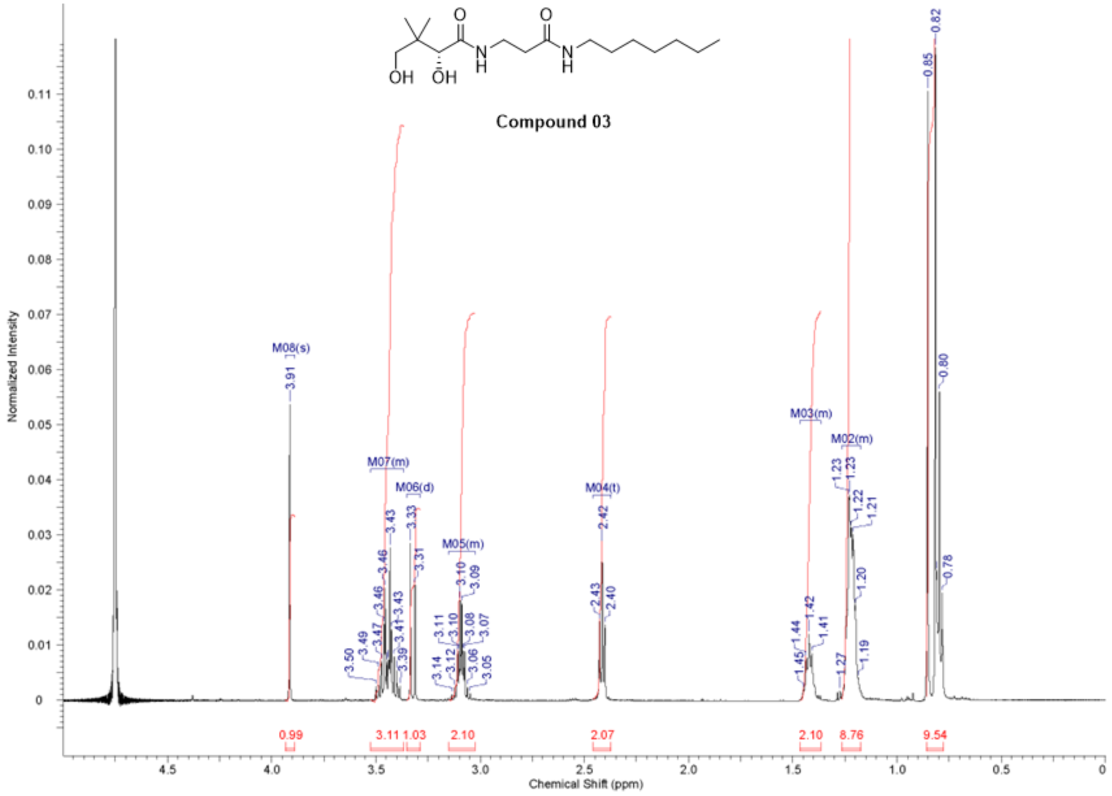

Compound 03
OH
OH
O
O
N
H
N
H
Normalized Intensity
Chemical Shift (ppm)
M08(s)
M07(m)
M06(d)
M05(m)
M04(t)
M03(m)
M02(m)
0.99
3.11
1.03
2.10
2.07
2.10
8.76
9.54

^{13}C NMR of **3**

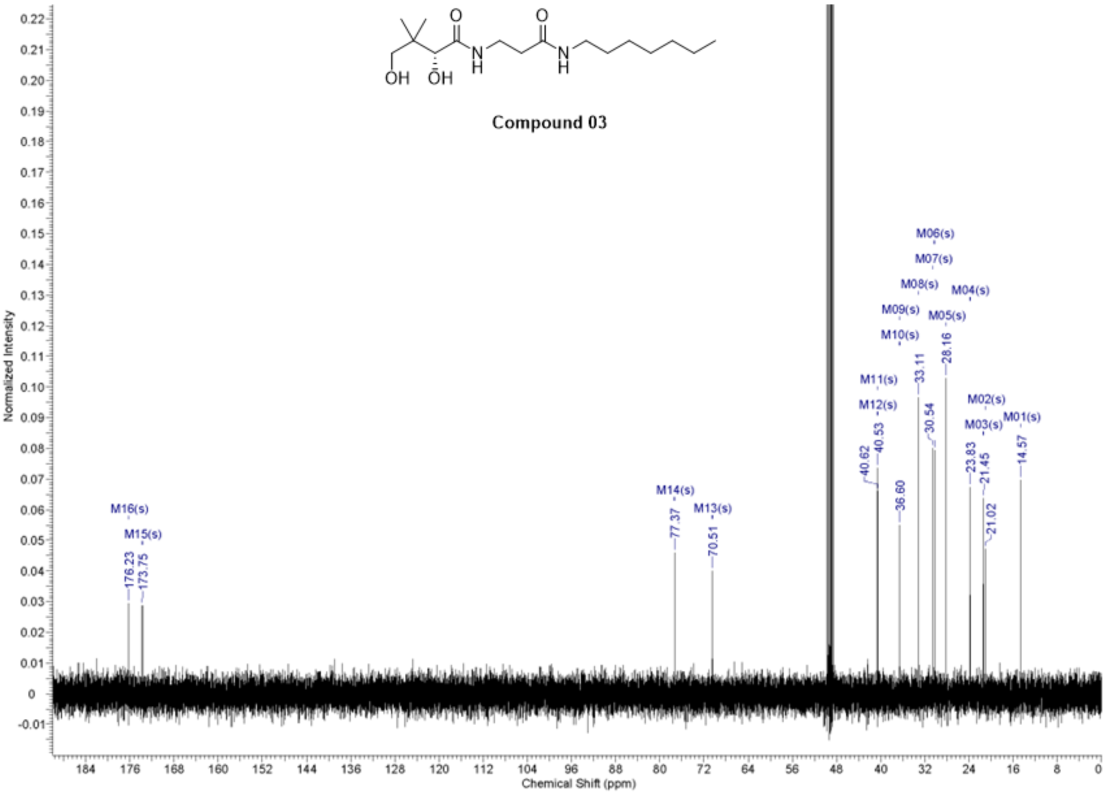

Compound 03
Normalized Intensity
Chemical Shift (ppm)
M16(s) 176.23
M15(s) 173.75
M14(s) 77.37
M13(s) 70.51
M12(s) 40.53
M11(s) 40.62
M10(s) 36.60
M09(s) 33.11
M08(s) 30.54
M07(s)
M06(s)
M05(s) 28.16
M04(s)
M03(s) 21.45
M02(s) 23.83
M01(s) 14.57
21.02

HRMS of **3**

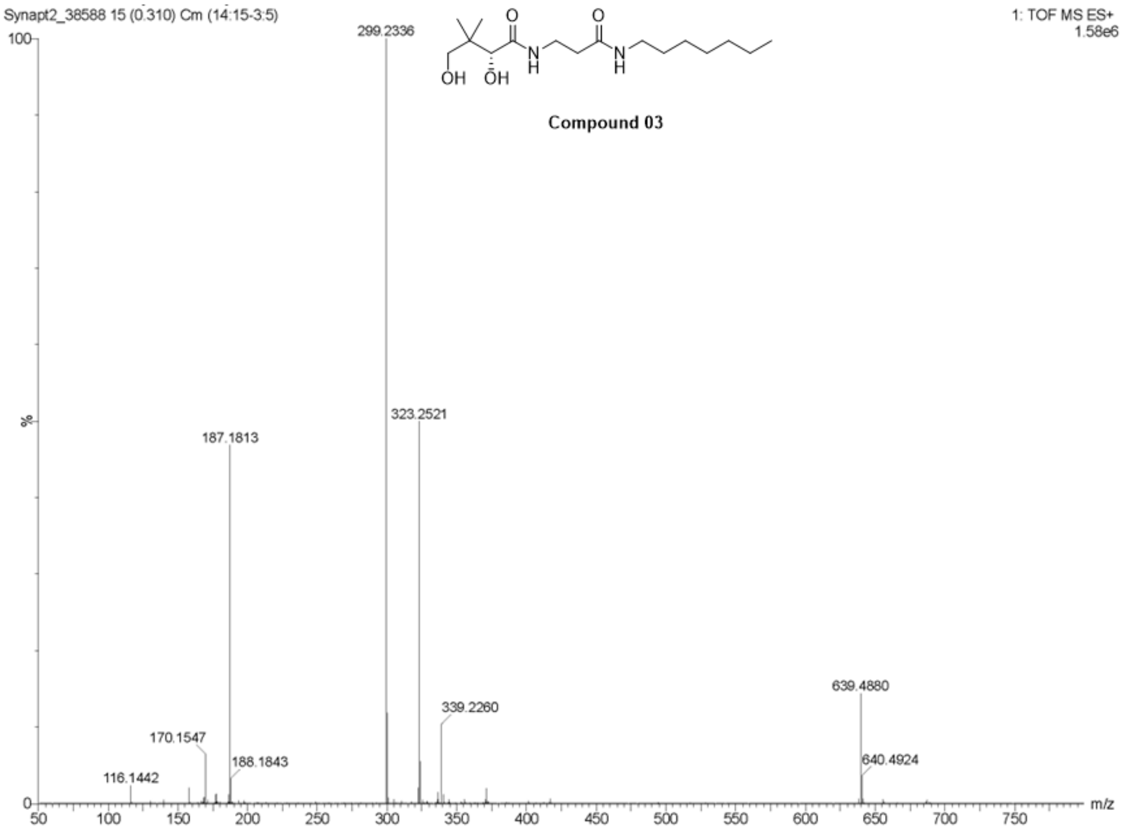
Synapt2_38588 15 (0.310) Cm (14:15-3:5)
1: TOF MS ES+
1.58e6
100
%
0
299.2336
323.2521
187.1813
639.4880
339.2260
170.1547
188.1843
116.1442
640.4924
50
100
150
200
250
300
350
400
450
500
550
600
650
700
750
m/z
OH
OH
O
O
N
H
N
H
Compound 03

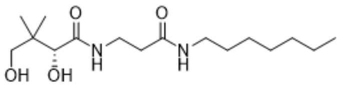

Elemental Composition Report | **Compound 03** | **Page 1**

Single Mass Analysis

Tolerance = 5.0 PPM / DBE: min = -1.5, max = 50.0
Element prediction: Off
Number of isotope peaks used for i-FIT = 3

Monoisotopic Mass, Even Electron Ions
380 formula(e) evaluated with 2 results within limits (up to 50 best isotopic matches for each mass)
Elements Used:
C: 0-50 H: 0-80 N: 0-5 O: 0-7 Na: 0-1

Order# 22624 Meyers Lab djm-14-25C-01 | MSL, SCS, UIUC | SYNAPTG2-Si#UGA354
Synapt2_38588 15 (0.310) Cm (14:15-3:5) | 1: TOF MS ES+ | 1.64e+005

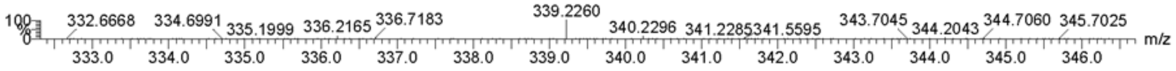

Mass	Calc. Mass	mDa	PPM	DBE	i-FIT	Norm	Conf(%)	Formula
Minimum:				-1.5				
Maximum:		5.0	5.0	50.0				
339.2260	339.2244	1.6	4.7	0.5	219.9	0.140	86.98	C13 H31 N4 O6
	339.2260	0.0	0.0	1.5	221.8	2.039	13.02	C16 H32 N2 O4 Na

^{1}H NMR of **4**

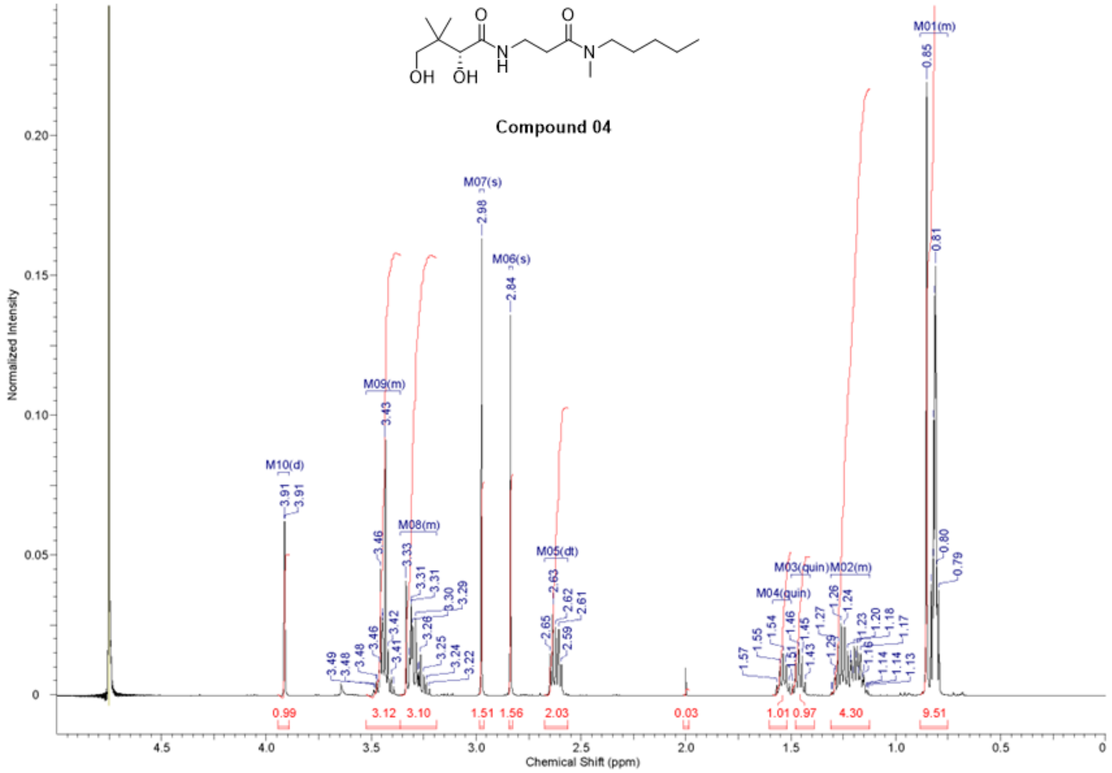
Compound 04
M01(m)
M02(m)
M03(quin)
M04(quin)
M05(dt)
M06(s)
M07(s)
M08(m)
M09(m)
M10(d)
Normalized Intensity
Chemical Shift (ppm)

^{13}C NMR of **4**

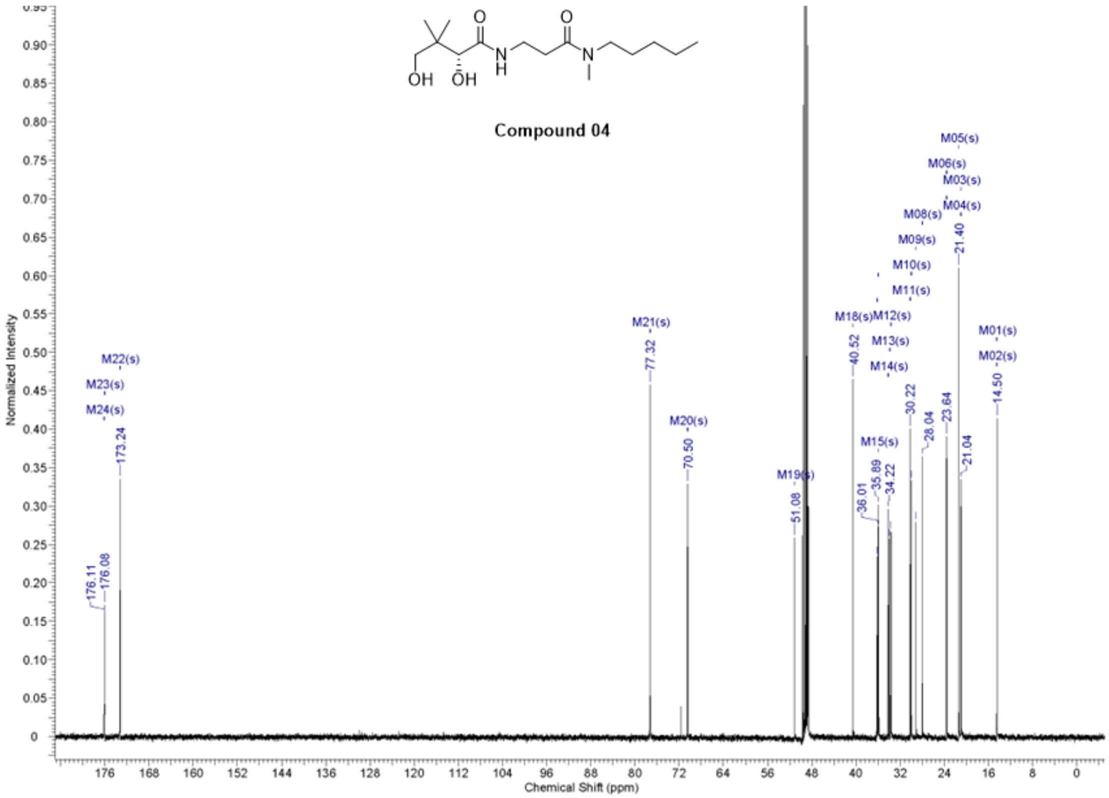
Compound 04
Normalized Intensity
Chemical Shift (ppm)
176.11
176.08
173.24
77.32
70.50
51.08
40.52
36.01
35.89
34.22
30.22
28.04
23.64
21.40
21.04
14.50

HRMS of **4**

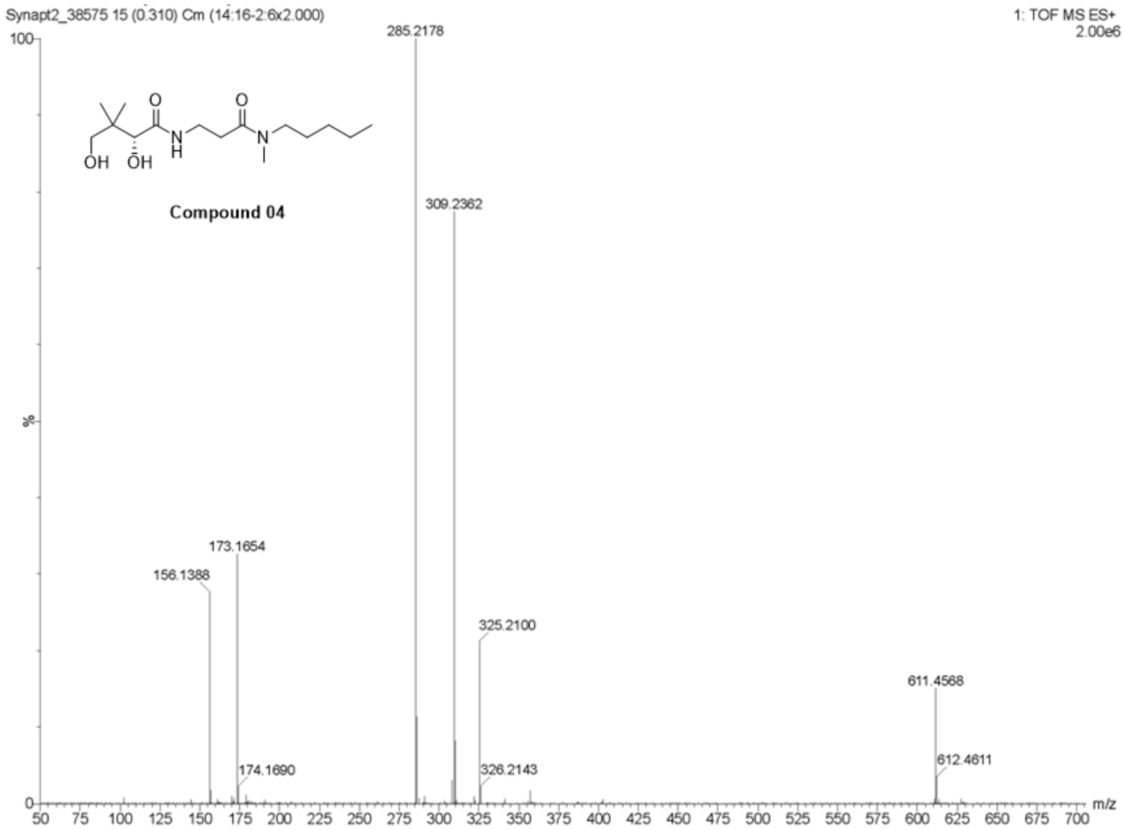

Synapt2_38575 15 (0.310) Cm (14:16-2:6x2.000)
1: TOF MS ES+
2.00e6
100
%
0
285.2178
309.2362
173.1654
156.1388
325.2100
611.4568
612.4611
174.1690
326.2143
Compound 04
OH
OH
N
H
O
O
N
m/z
50 75 100 125 150 175 200 225 250 275 300 325 350 375 400 425 450 475 500 525 550 575 600 625 650 675 700

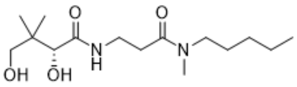

Compound 04

Elemental Composition Report **Page 1**

Single Mass Analysis
Tolerance = 5.0 PPM / DBE: min = -1.5, max = 50.0
Element prediction: Off
Number of isotope peaks used for i-FIT = 3

Monoisotopic Mass, Even Electron Ions
364 formula(e) evaluated with 2 results within limits (up to 50 best isotopic matches for each mass)
Elements Used:
C: 0-50 H: 0-80 N: 0-5 O: 0-7 Na: 0-1
Order# 22610 Meyers Lab djm-14-25A-01
Synapt2_38575 15 (0.310) Cm (14:16-2:6x2.000)
MSL, SCS, UIUC
SYNAPTG2-Si#UGA354
1: TOF MS ES+
4.23e+005

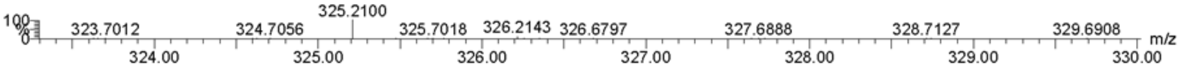

Minimum:				-1.5				
Maximum:		5.0	5.0	50.0				
Mass	Calc. Mass	mDa	PPM	DBE	i-FIT	Norm	Conf(%)	Formula
325.2100	325.2103	-0.3	-0.9	1.5	105.7	0.167	84.65	C15 H30 N2 O4 Na
	325.2087	1.3	4.0	0.5	107.4	1.874	15.35	C12 H29 N4 O6

^{1}H NMR of **5**

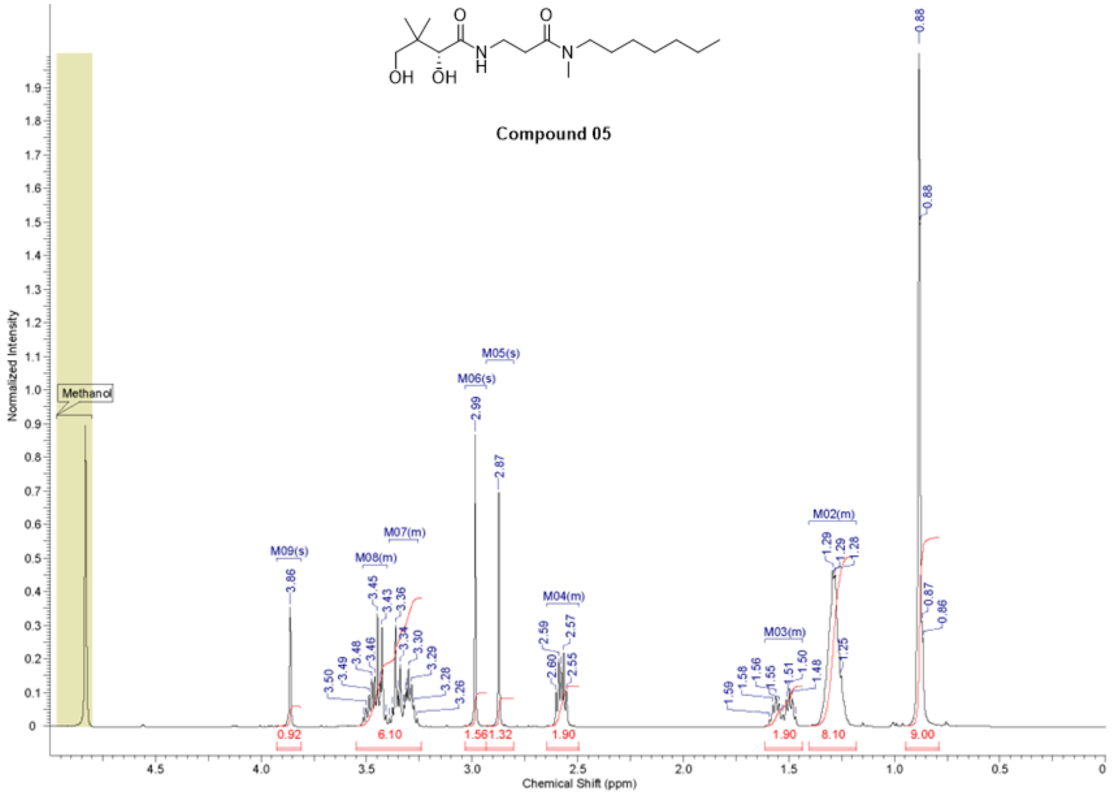
Compound 05
Methanol
M09(s)
M08(m)
M07(m)
M06(s)
M05(s)
M04(m)
M03(m)
M02(m)
Normalized Intensity
Chemical Shift (ppm)

^{13}C NMR of **5**

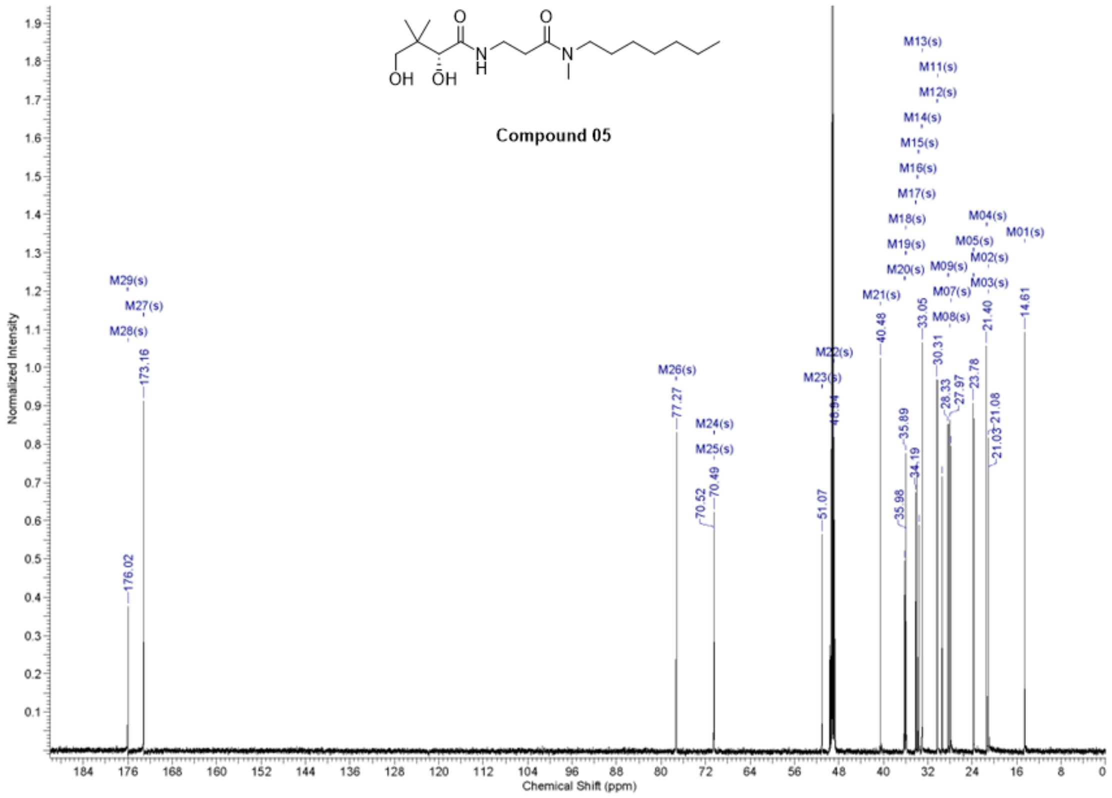

Compound 05
Normalized Intensity
Chemical Shift (ppm)
176.02
173.16
77.27
70.52
70.49
51.07
48.94
40.48
35.98
35.89
34.19
33.05
30.31
28.33
27.97
23.78
21.40
21.08
21.03
14.61

HRMS of **5**

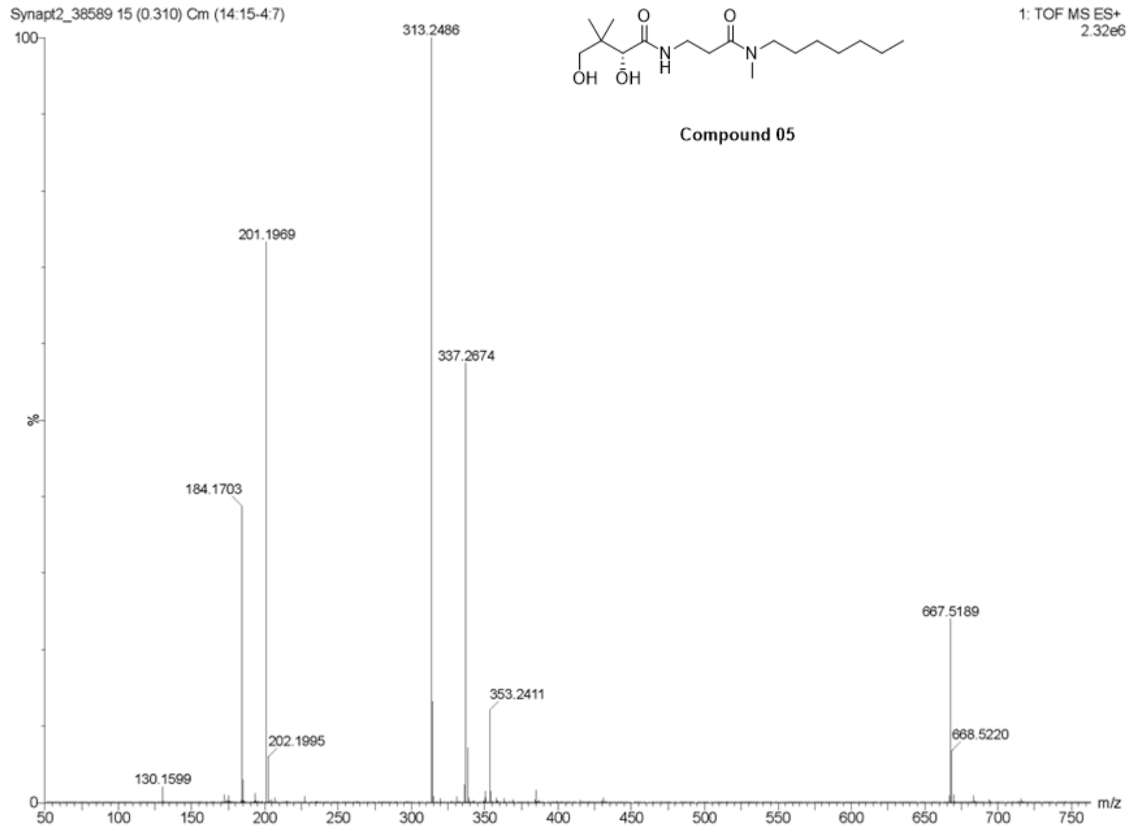
Synapt2_38589 15 (0.310) Cm (14:15-4:7)
1: TOF MS ES+
2.32e6
Compound 05
OH
OH
N
H
O
O
N
313.2486
201.1969
337.2674
184.1703
667.5189
353.2411
202.1995
668.5220
130.1599
%
100
0
m/z
50
100
150
200
250
300
350
400
450
500
550
600
650
700
750

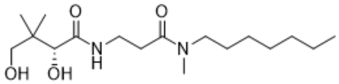

Elemental Composition Report Compound 05 **Page 1**

Single Mass Analysis
Tolerance = 5.0 PPM / DBE: min = -1.5, max = 50.0
Element prediction: Off
Number of isotope peaks used for i-FIT = 3

Monoisotopic Mass, Even Electron Ions
396 formula(e) evaluated with 2 results within limits (up to 50 best isotopic matches for each mass)
Elements Used:
C: 0-50 H: 0-80 N: 0-5 O: 0-7 Na: 0-1

Order# 22625 Meyers Lab djm-14-25D-01 MSL, SCS, UIUC SYNAPTG2-Si#UGA354
Synapt2_38589 15 (0.310) Cm (14:15-4:7) 1: TOF MS ES+
2.79e+005

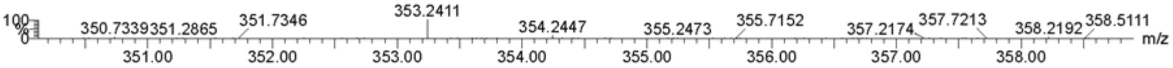

Minimum:				-1.5				
Maximum:		5.0	5.0	50.0				
Mass	Calc. Mass	mDa	PPM	DBE	i-FIT	Norm	Conf(%)	Formula
353.2411	353.2416	-0.5	-1.4	1.5	177.6	0.074	92.83	C17 H34 N2 O4 Na
	353.2400	1.1	3.1	0.5	180.2	2.635	7.17	C14 H33 N4 O6

^{1}H NMR of **6**

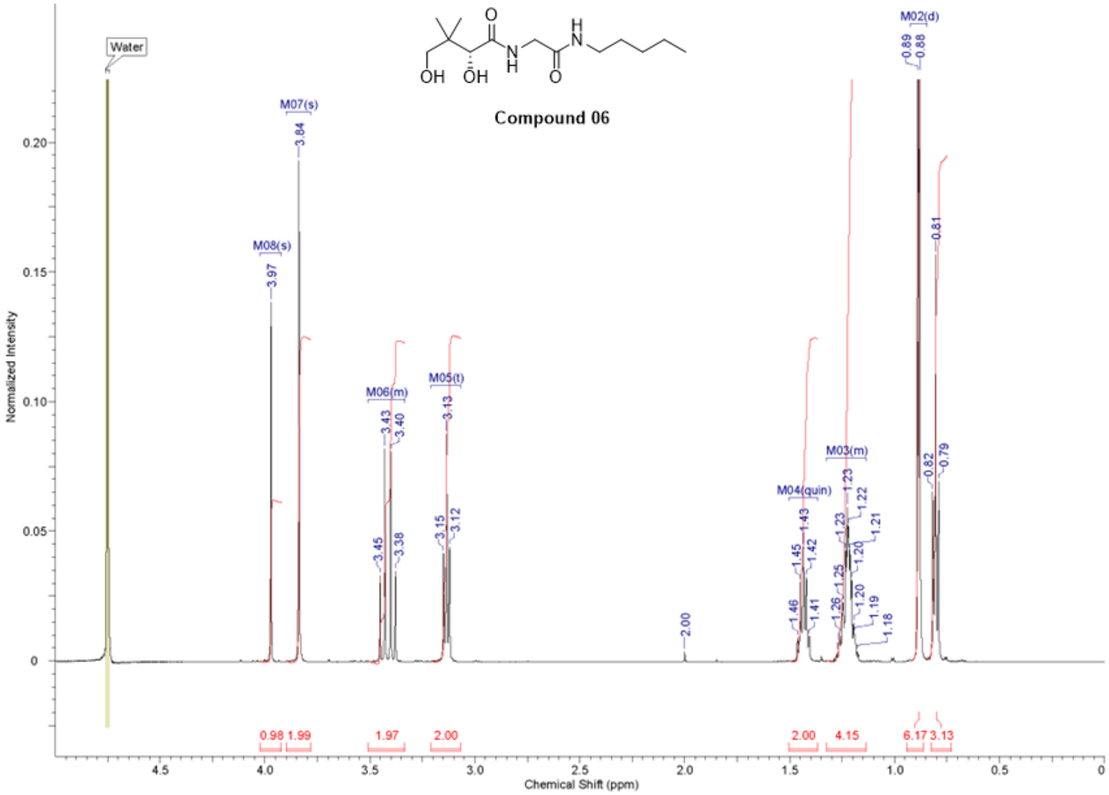
Compound 06
Water
M07(s)
M08(s)
M06(m)
M05(t)
M04(quin)
M03(m)
M02(d)
Normalized Intensity
Chemical Shift (ppm)
0.98 1.99 1.97 2.00 2.00 4.15 6.17 3.13

^{13}C NMR of **6**

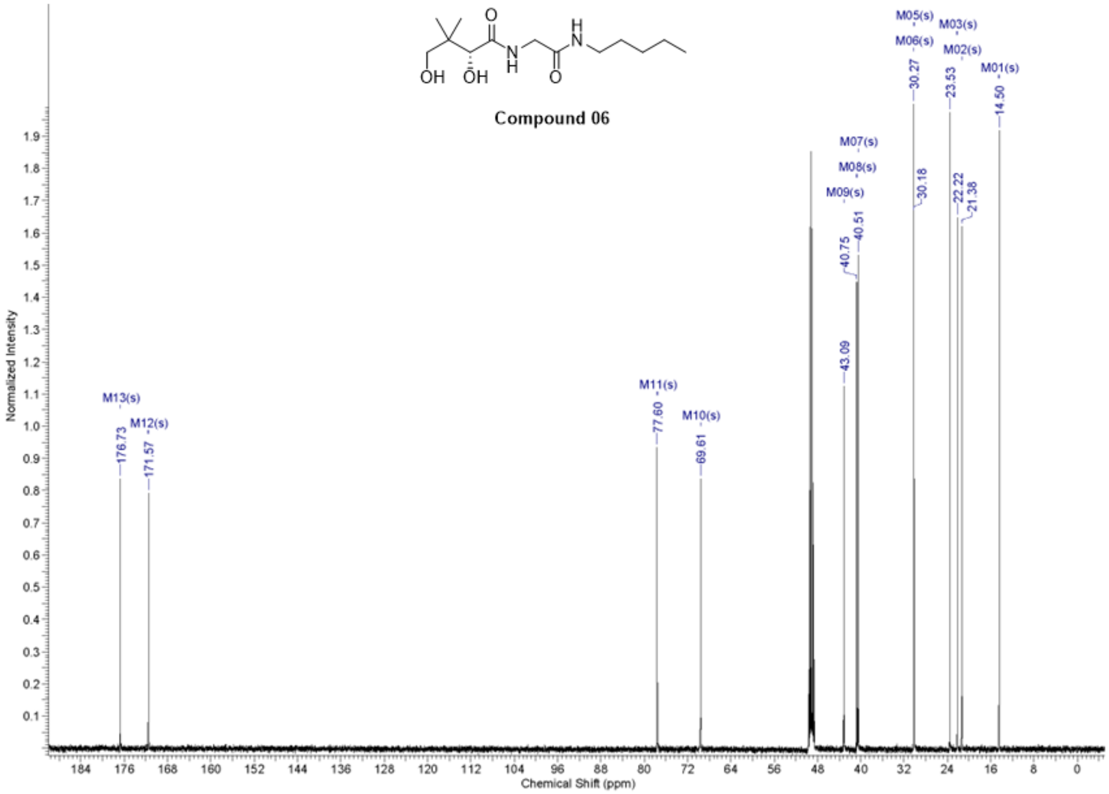
Compound 06
M13(s)
176.73
M12(s)
171.57
M11(s)
77.60
M10(s)
69.61
43.09
M09(s)
40.75
M08(s)
40.51
M07(s)
M05(s)
M06(s)
30.27
30.18
M03(s)
M02(s)
23.53
22.22
21.38
M01(s)
14.50
Normalized Intensity
Chemical Shift (ppm)

HRMS of **6**

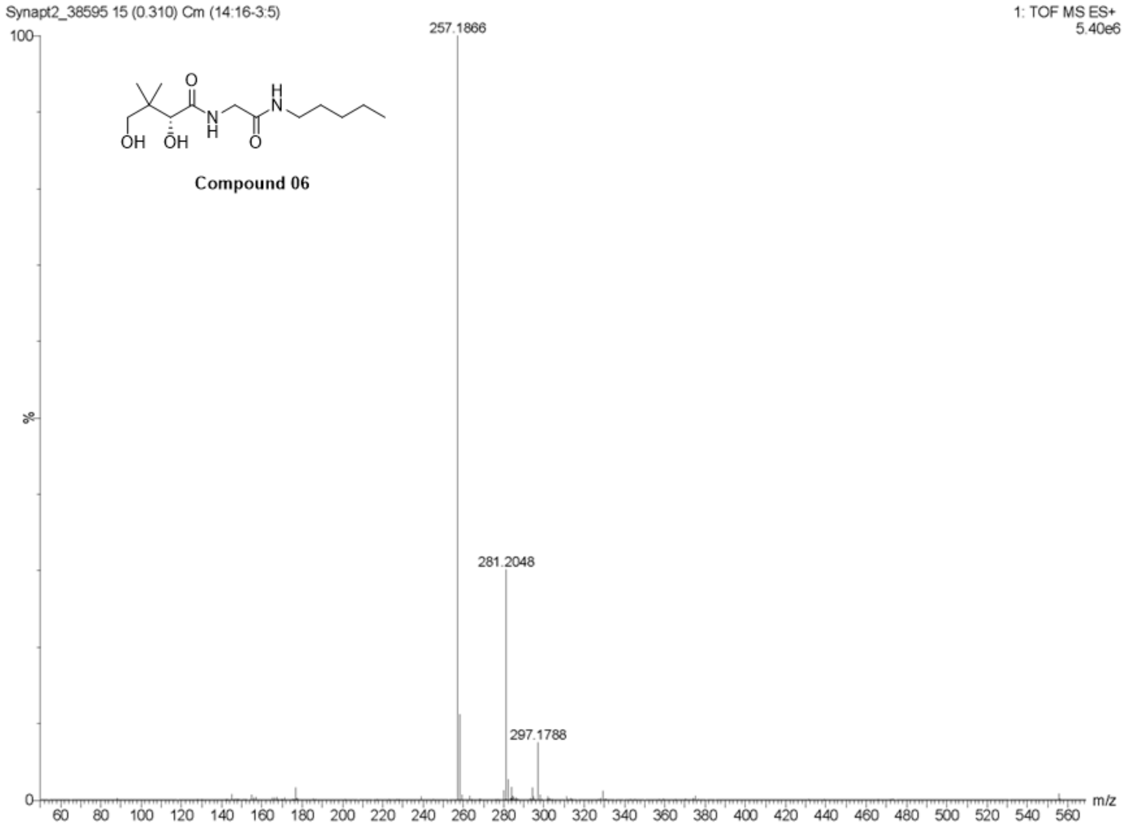

Synapt2_38595 15 (0.310) Cm (14:16-3:5)
1: TOF MS ES+
5.40e6
257.1866
281.2048
297.1788
Compound 06
%
m/z

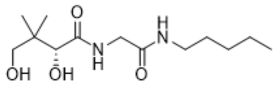

Compound 06

Elemental Composition Report

Page 1

Single Mass Analysis

Tolerance = 5.0 PPM / DBE: min = -1.5, max = 50.0
Element prediction: Off
Number of isotope peaks used for i-FIT = 3

Monoisotopic Mass, Even Electron Ions
332 formula(e) evaluated with 2 results within limits (up to 50 best isotopic matches for each mass)
Elements Used:
C: 0-50 H: 0-80 N: 0-5 O: 0-7 Na: 0-1

Order# 22639 Meyers Lab djm-13-144E-01-c18-01-f16 MSL, SCS, UIUC SYNAPTG2-Si#UGA354
Synapt2_38595 15 (0.310) Cm (14:16-3:5) 1: TOF MS ES+
4.08e+005

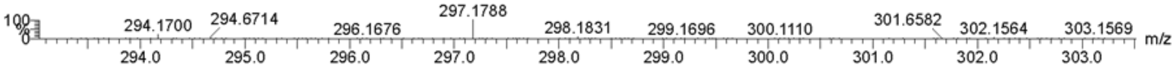

Minimum:				-1.5				
Maximum:		5.0	5.0	50.0				
Mass	Calc. Mass	mDa	PPM	DBE	i-FIT	Norm	Conf(%)	Formula
297.1788	297.1774	1.4	4.7	0.5	346.6	0.152	85.88	C10 H25 N4 O6
	297.1790	-0.2	-0.7	1.5	348.4	1.958	14.12	C13 H26 N2 O4 Na

^{1}H NMR of **7**

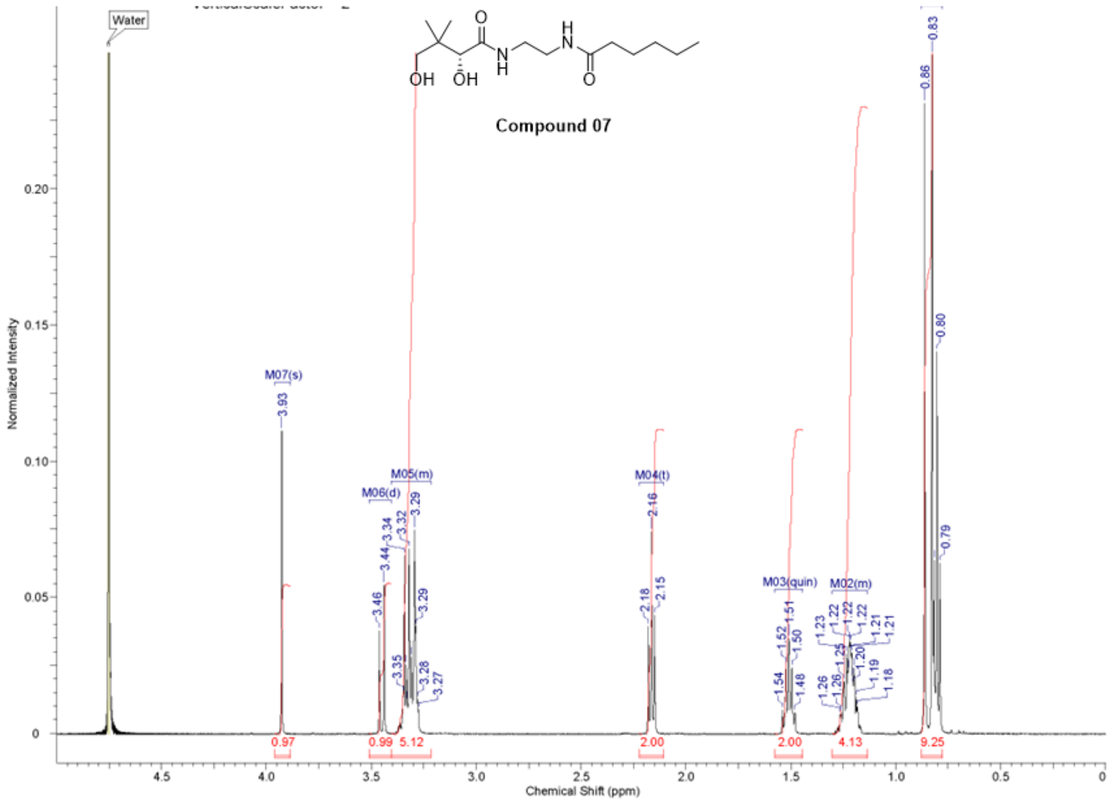
Water
Compound 07
M07(s)
M06(d)
M05(m)
M04(t)
M03(quin)
M02(m)
Normalized Intensity
Chemical Shift (ppm)

^{13}C NMR of 7

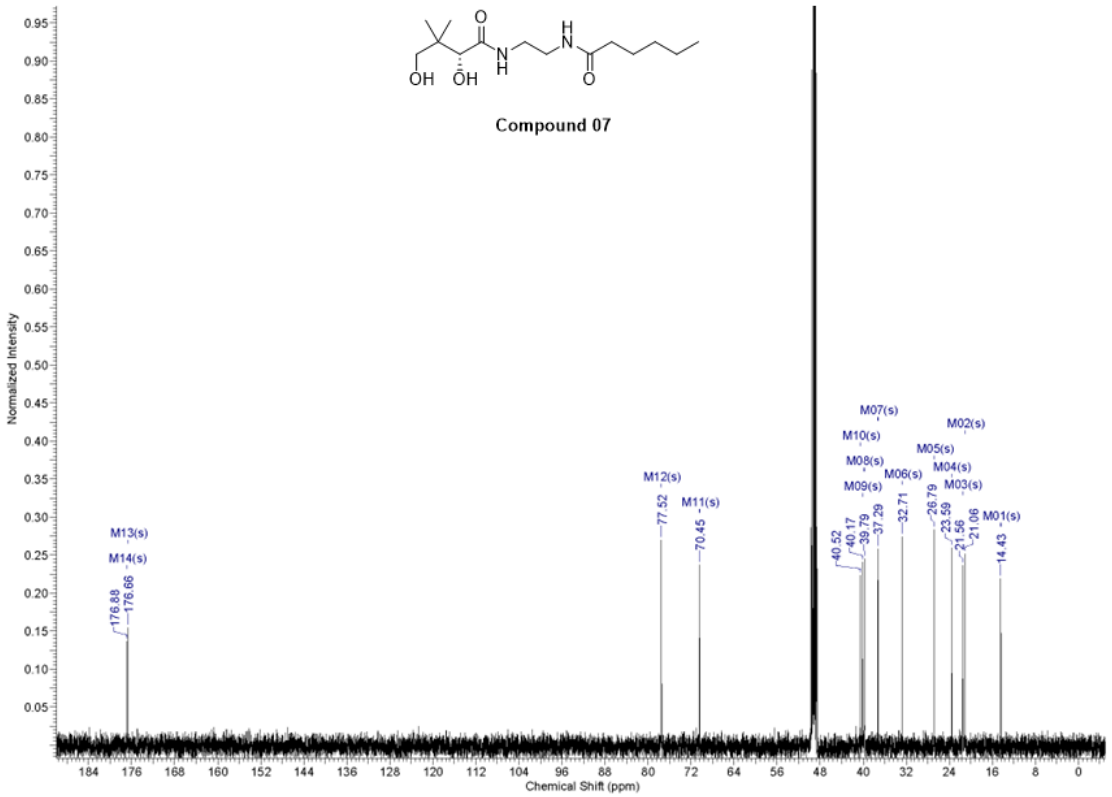
Compound 07
Normalized Intensity
Chemical Shift (ppm)
M13(s)
M14(s)
176.88
176.66
M12(s)
77.52
M11(s)
70.45
M10(s)
M08(s)
M09(s)
M07(s)
40.52
40.17
39.79
37.29
M06(s)
32.71
M05(s)
26.79
M02(s)
M04(s)
M03(s)
23.59
21.56
21.06
M01(s)
14.43

HRMS of 7

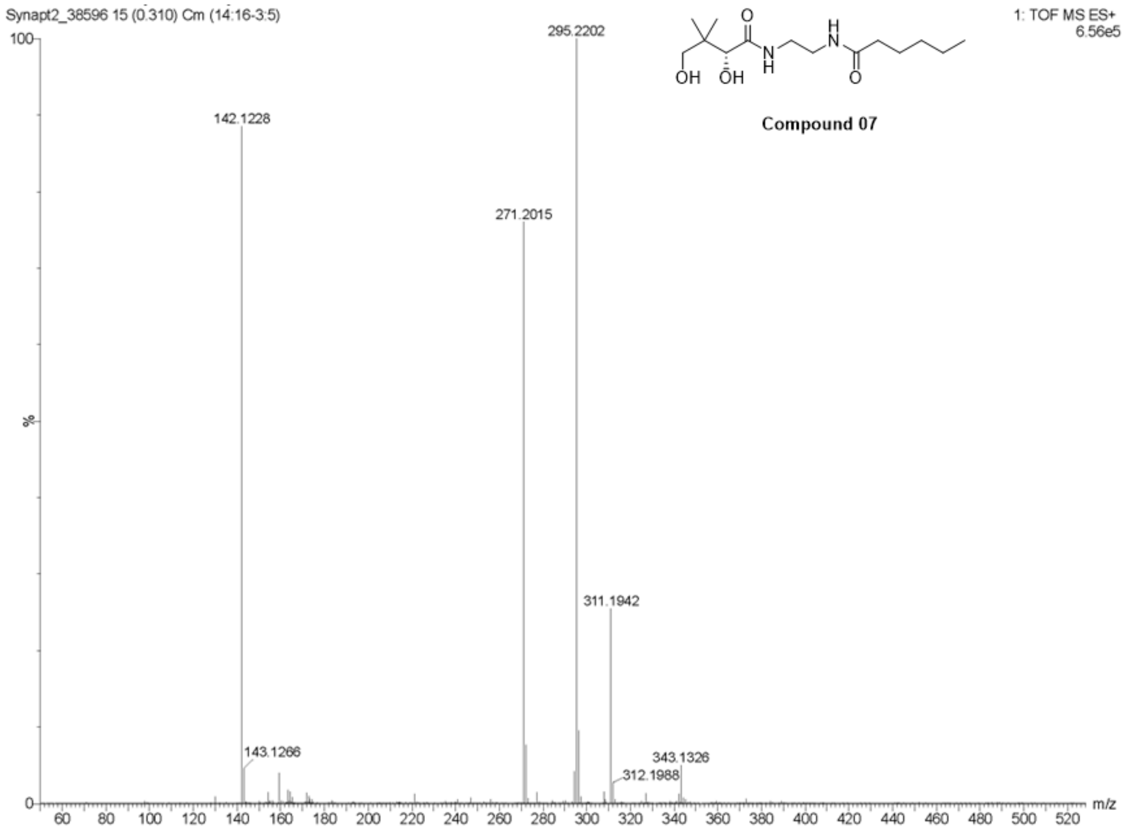

Synapt2_38596 15 (0.310) Cm (14:16-3:5)
1: TOF MS ES+
6.56e5
Compound 07
OH
OH
N
H
H
N
O
O
295.2202
142.1228
271.2015
311.1942
143.1266
343.1326
312.1988
100
%
0
60
80
100
120
140
160
180
200
220
240
260
280
300
320
340
360
380
400
420
440
460
480
500
520
m/z

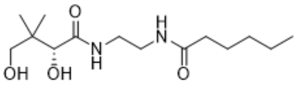

Elemental Composition Report **Compound 07** **Page 1**

Single Mass Analysis

Tolerance = 5.0 PPM / DBE: min = -1.5, max = 50.0
Element prediction: Off
Number of isotope peaks used for i-FIT = 3

Monoisotopic Mass, Even Electron Ions
348 formula(e) evaluated with 2 results within limits (up to 50 best isotopic matches for each mass)
Elements Used:
C: 0-50 H: 0-80 N: 0-5 O: 0-7 Na: 0-1

Order# 22640 Meyers Lab djm-13-144F-01-C18-01 f37-38 MSL, SCS, UIUC SYNAPTG2-Si#UGA354
Synapt2_38596 15 (0.310) Cm (14:16-3:5) 1: TOF MS ES+
1.67e+005

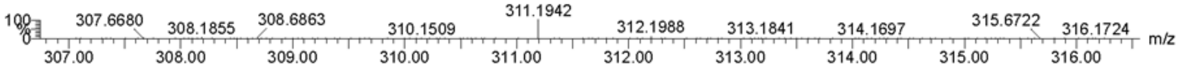

Minimum:				-1.5				
Maximum:		5.0	5.0	50.0				
Mass	Calc. Mass	mDa	PPM	DBE	i-FIT	Norm	Conf(%)	Formula
311.1942	311.1931	1.1	3.5	0.5	317.1	0.096	90.88	C11 H27 N4 O6
	311.1947	-0.5	-1.6	1.5	319.4	2.394	9.12	C14 H28 N2 O4 Na

^{1}H NMR of **8**

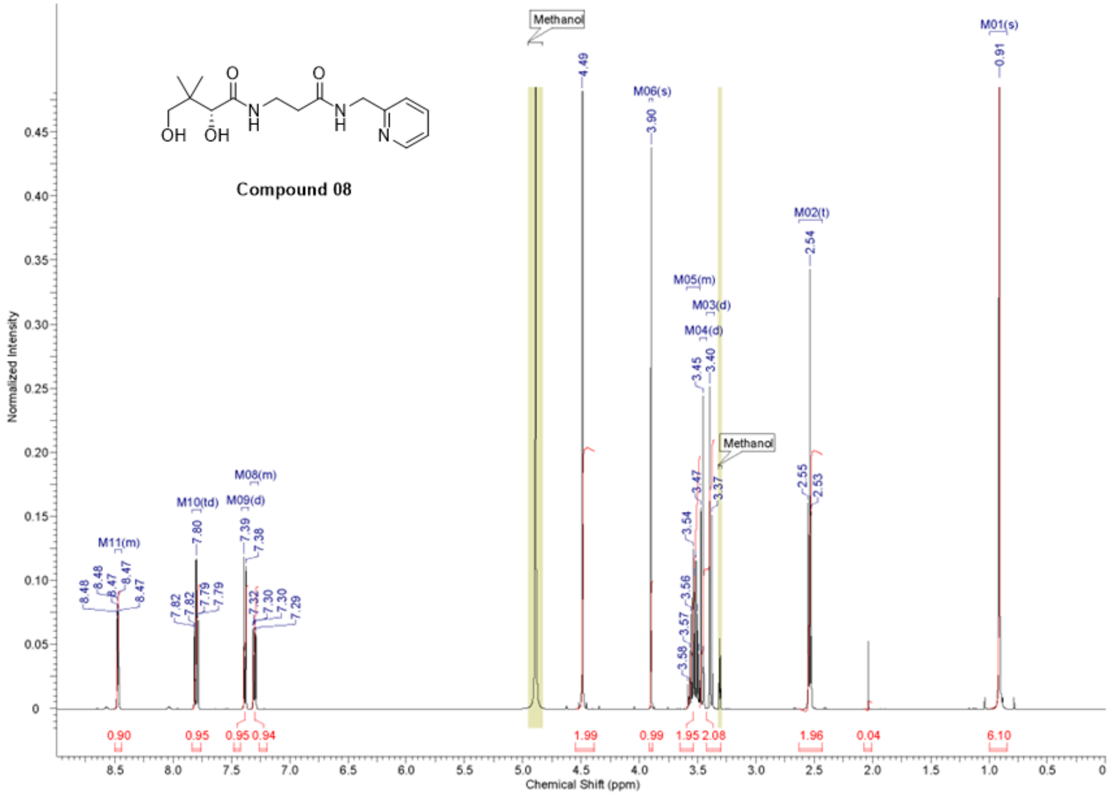
Compound 08
Methanol
M01(s)
M02(t)
M03(d)
M04(d)
M05(m)
M06(s)
M08(m)
M09(d)
M10(td)
M11(m)
Normalized Intensity
Chemical Shift (ppm)

^{13}C NMR of **8**

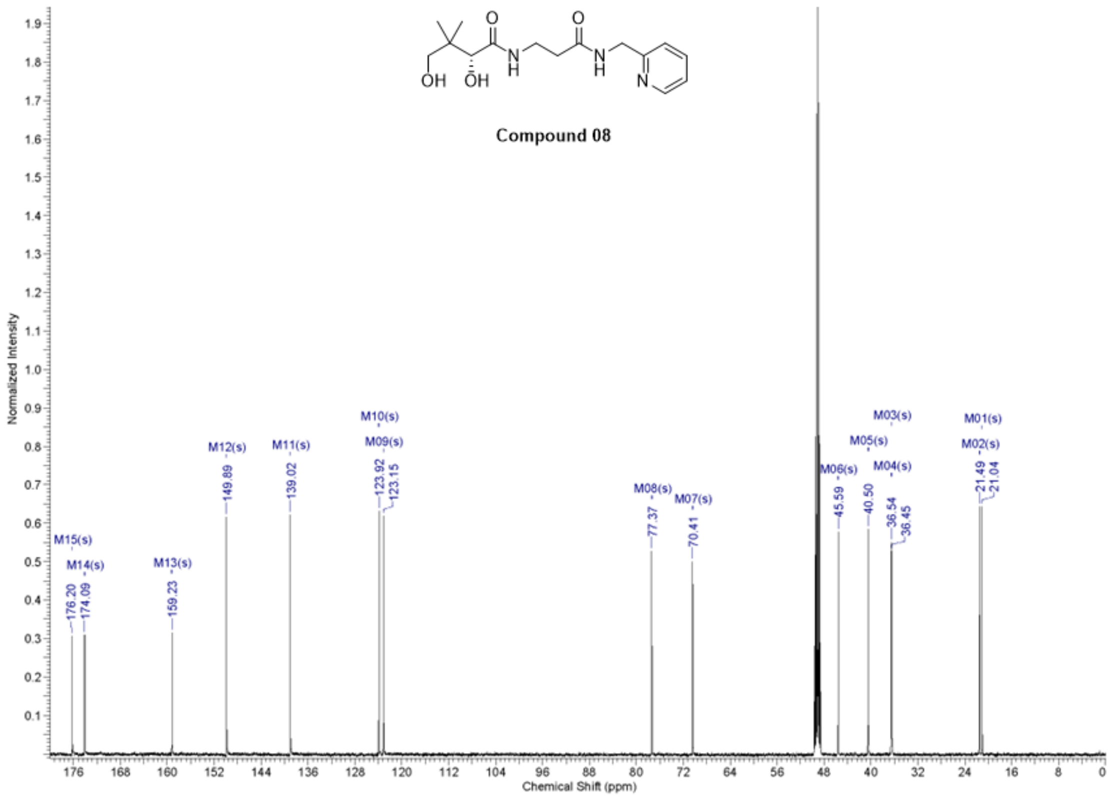
Compound 08
Normalized Intensity
Chemical Shift (ppm)
M15(s)
176.20
M14(s)
174.09
M13(s)
159.23
M12(s)
149.89
M11(s)
139.02
M10(s)
M09(s)
123.92
123.15
M08(s)
77.37
M07(s)
70.41
M06(s)
45.59
M05(s)
40.50
M03(s)
M04(s)
36.54
36.45
M01(s)
M02(s)
21.49
21.04

HRMS of **8**

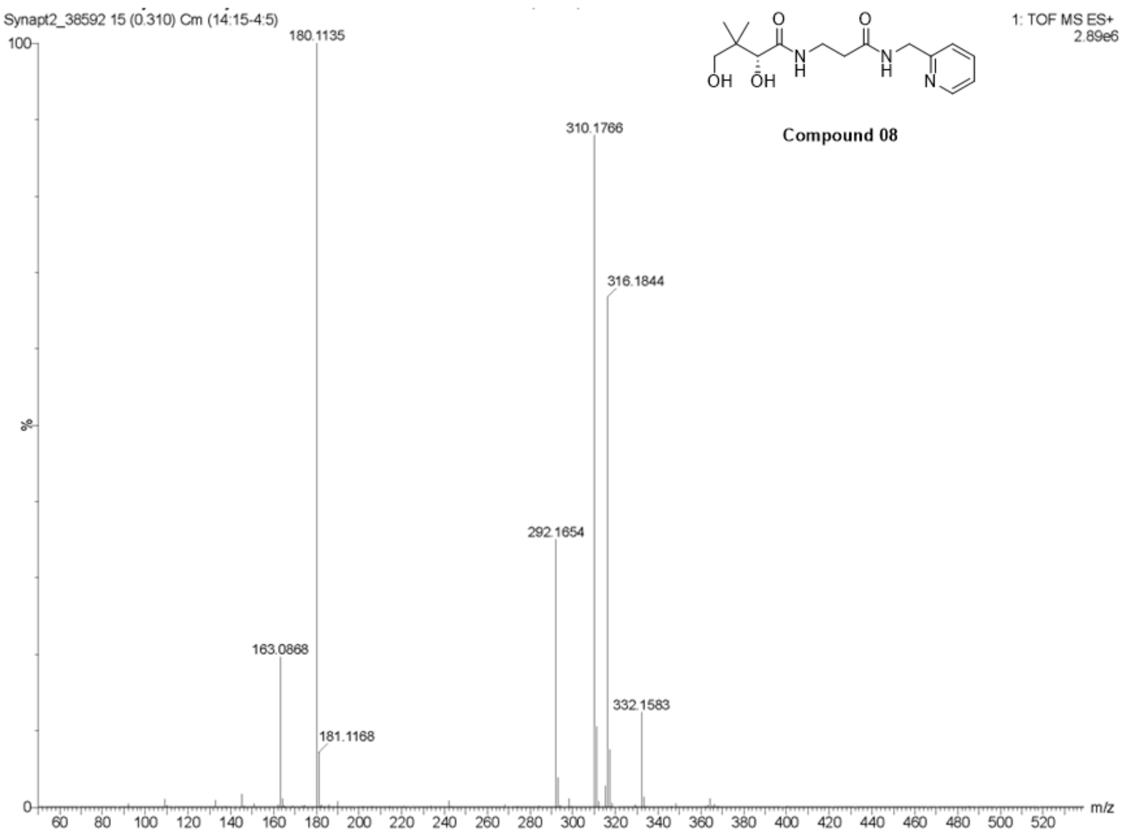
Synapt2_38592 15 (0.310) Cm (14:15-4:5)
1: TOF MS ES+
2.89e6
Compound 08
180.1135
310.1766
316.1844
292.1654
163.0868
332.1583
181.1168
%
m/z

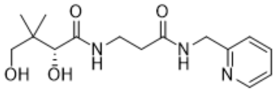

Elemental Composition Report **Compound 08** **Page 1**

Single Mass Analysis
Tolerance = 5.0 PPM / DBE: min = -1.5, max = 50.0
Element prediction: Off
Number of isotope peaks used for i-FIT = 3

Monoisotopic Mass, Even Electron Ions
374 formula(e) evaluated with 2 results within limits (up to 50 best isotopic matches for each mass)
Elements Used:
C: 0-50 H: 0-80 N: 0-5 O: 0-7 Na: 0-1

Order# 22630 Meyers Lab djm-14-25G-01 MSL, SCS, UIUC SYNAPTG2-Si#UGA354
Synapt2_38592 15 (0.310) Cm (14:15-4:5) 1: TOF MS ES+
3.59e+005

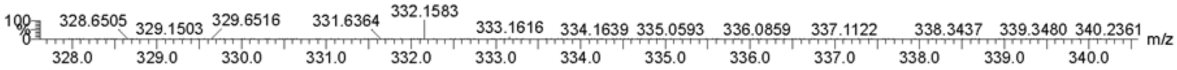

Minimum:				-1.5				
Maximum:		5.0	5.0	50.0				
Mass	Calc. Mass	mDa	PPM	DBE	i-FIT	Norm	Conf(%)	Formula
332.1583	332.1586	-0.3	-0.9	5.5	256.9	0.048	95.32	C15 H23 N3 O4 Na
	332.1570	1.3	3.9	4.5	259.9	3.062	4.68	C12 H22 N5 O6

^{1}H NMR of **9**

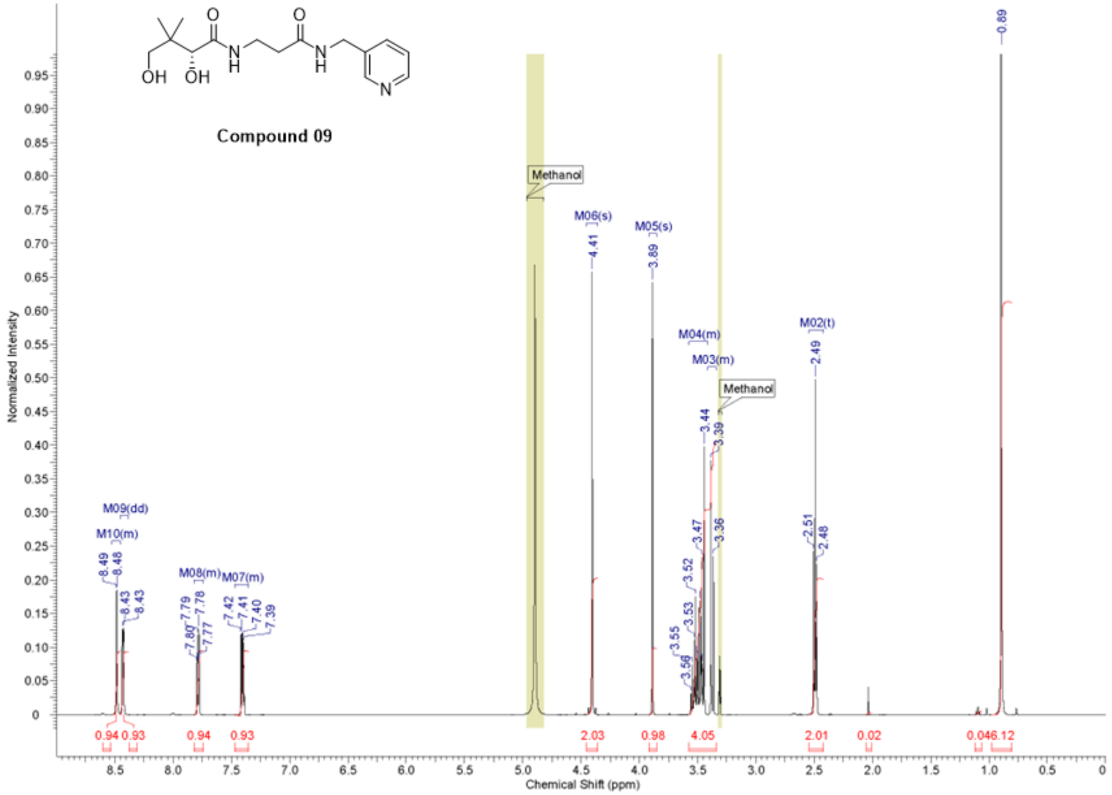

Compound 09
Methanol
Normalized Intensity
Chemical Shift (ppm)
M10(m)
M09(dd)
M08(m)
M07(m)
M06(s)
M05(s)
M04(m)
M03(m)
M02(t)

^{13}C NMR of **9**

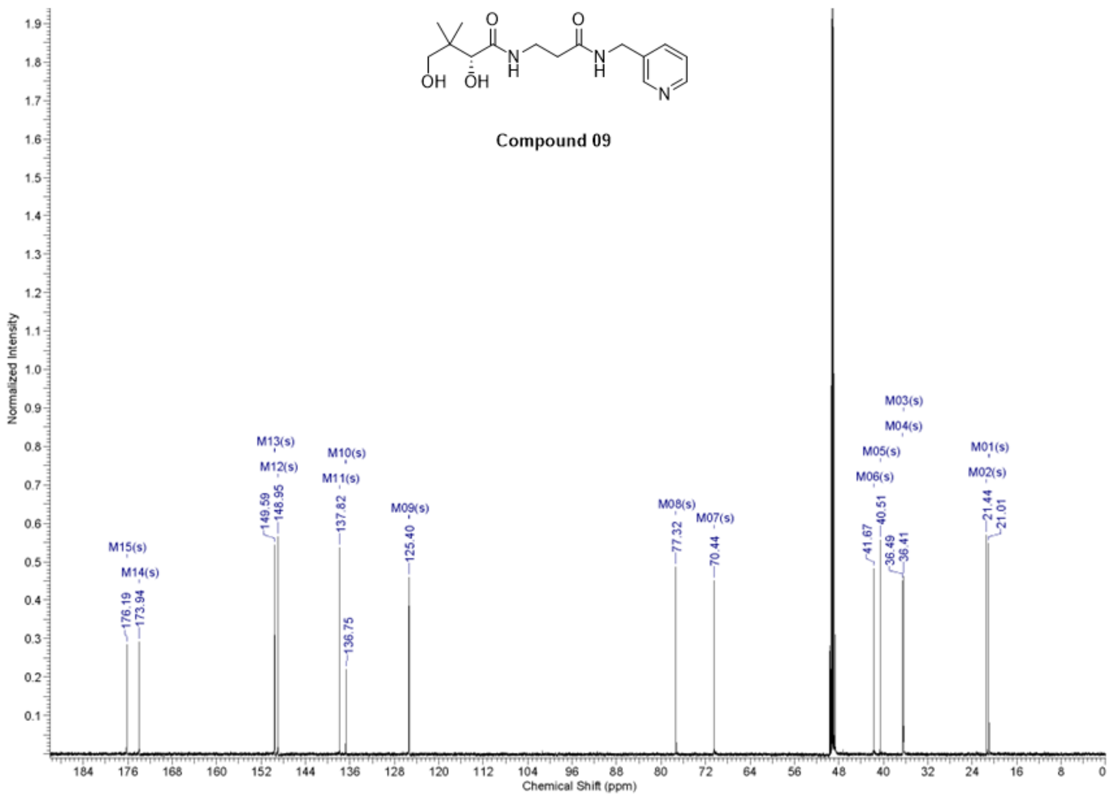
Compound 09
Normalized Intensity
Chemical Shift (ppm)
M15(s) 176.19
M14(s) 173.94
M13(s) 149.59
M12(s) 148.95
M10(s) 137.82
M11(s) 136.75
M09(s) 125.40
M08(s) 77.32
M07(s) 70.44
M06(s) 41.67
M05(s) 40.51
M04(s) 36.49
M03(s) 36.41
M01(s) 21.44
M02(s) 21.01

HRMS of **9**

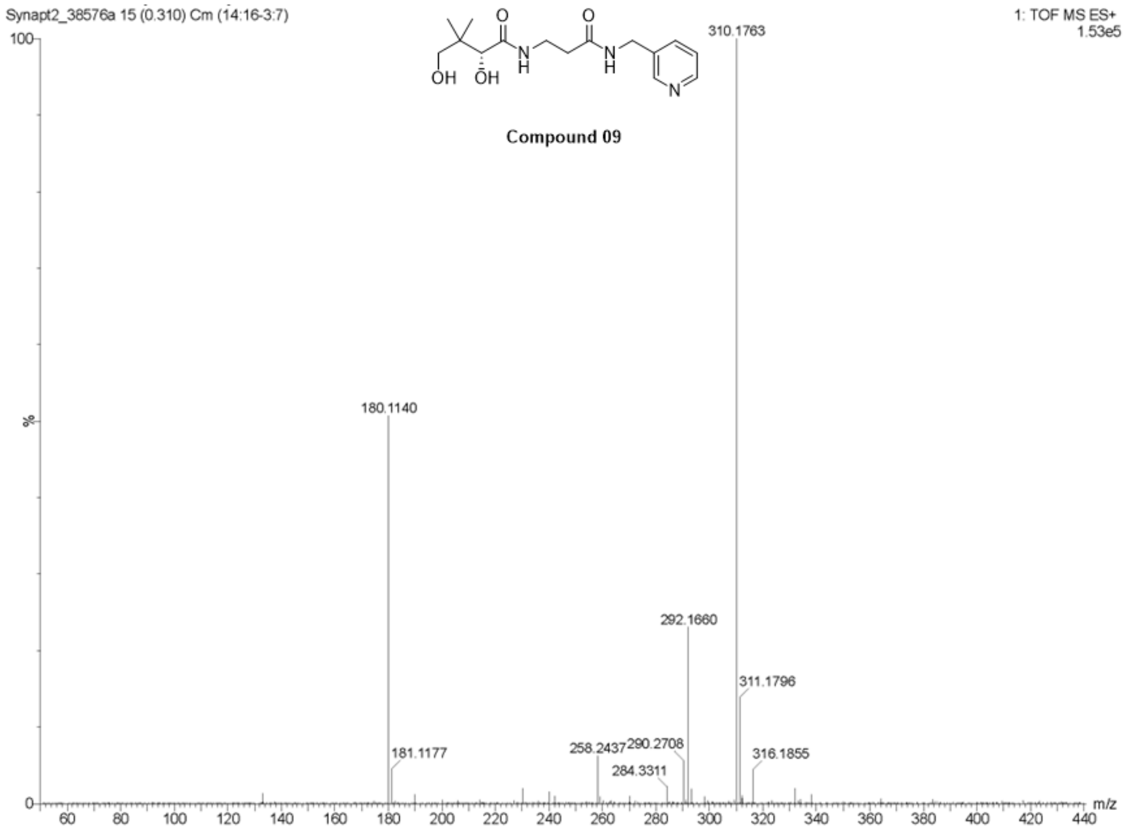

Synapt2_38576a 15 (0.310) Cm (14:16-3:7)
1: TOF MS ES+
1.53e5
Compound 09
310.1763
180.1140
292.1660
311.1796
181.1177
258.2437
290.2708
284.3311
316.1855
100
%
0
m/z

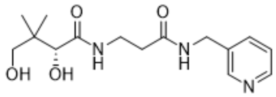

Elemental Composition Report Compound 09 **Page 1**

Single Mass Analysis
Tolerance = 5.0 PPM / DBE: min = -1.5, max = 50.0
Element prediction: Off
Number of isotope peaks used for i-FIT = 3

Monoisotopic Mass, Even Electron Ions
348 formula(e) evaluated with 1 results within limits (up to 50 best isotopic matches for each mass)
Elements Used:
C: 0-50 H: 0-80 N: 0-5 O: 0-7 Na: 0-1

Order# 22611 Meyers Lab djm-14-25B-01 MSL, SCS, UIUC SYNAPTG2-Si#UGA354
Synapt2_38576a 15 (0.310) Cm (14:16-3:7) 1: TOF MS ES+
1.53e+005

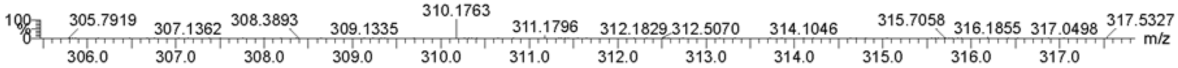

Minimum:				-1.5				
Maximum:		5.0	5.0	50.0				
Mass	Calc. Mass	mDa	PPM	DBE	i-FIT	Norm	Conf(%)	Formula
310.1763	310.1767	-0.4	-1.3	5.5	144.1	n/a	n/a	C15 H24 N3 O4

^{1}H NMR of **10**

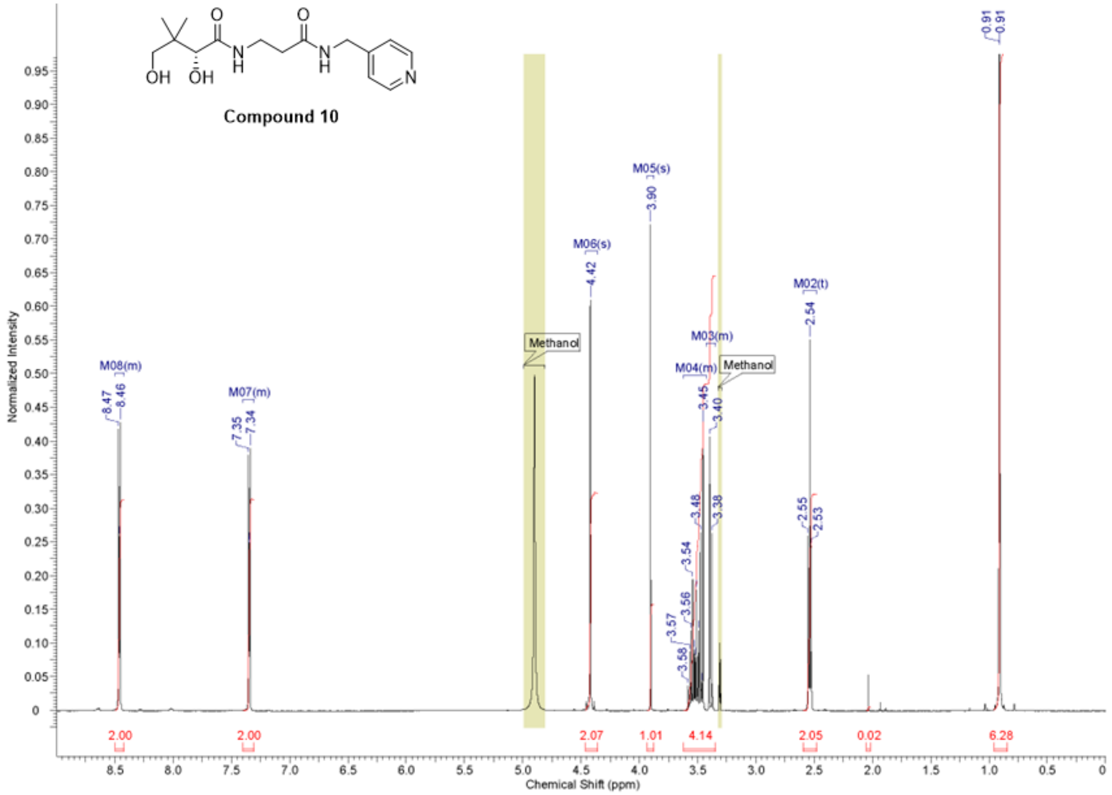
Compound 10
Normalized Intensity
Chemical Shift (ppm)
M08(m)
8.47
8.46
M07(m)
7.35
7.34
Methanol
M06(s)
4.42
M05(s)
3.90
M04(m)
M03(m)
3.58
3.57
3.56
3.54
3.48
3.45
3.40
3.38
Methanol
M02(t)
2.55
2.54
2.53
0.91
0.91
2.00
2.00
2.07
1.01
4.14
2.05
0.02
6.28

^{13}C NMR of **10**

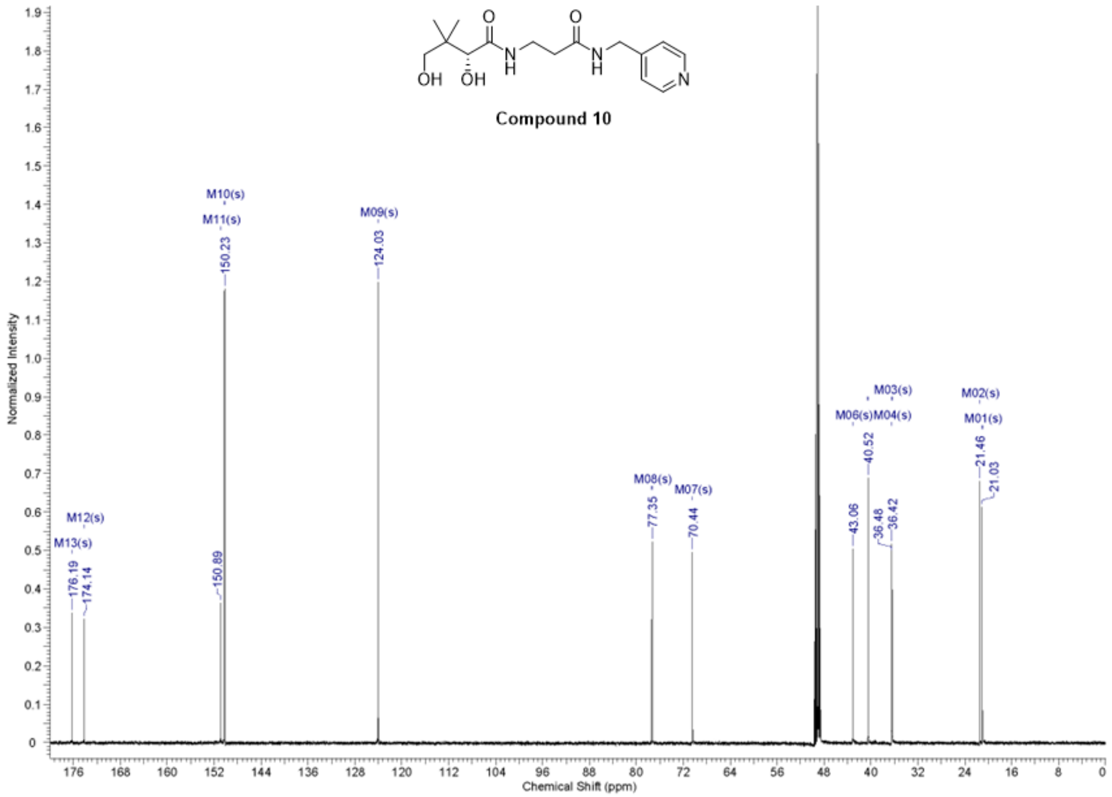

Compound 10
OH
OH
O
O
N
H
N
H
N
M13(s)
M12(s)
176.19
174.14
M10(s)
M11(s)
150.89
150.23
M09(s)
124.03
M08(s)
77.35
M07(s)
70.44
M06(s)
M03(s)
M04(s)
43.06
40.52
36.48
36.42
M02(s)
M01(s)
21.46
21.03
Normalized Intensity
Chemical Shift (ppm)

HRMS of **10**

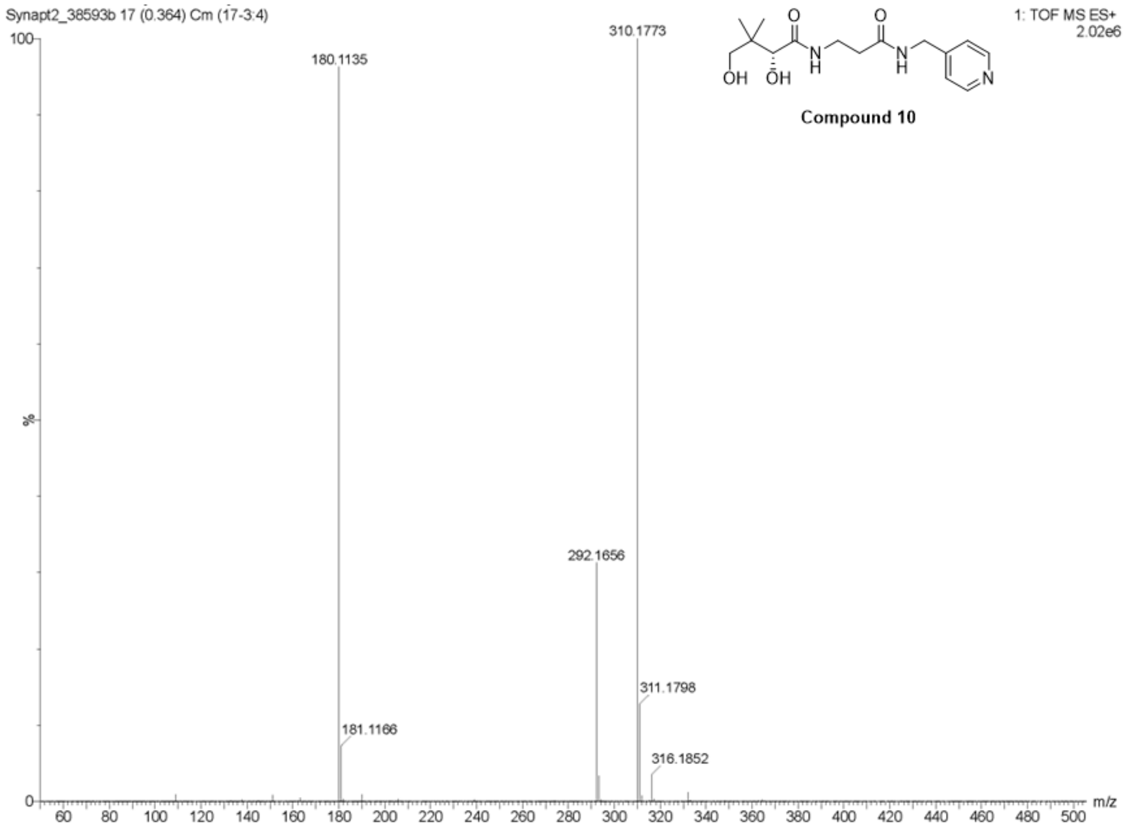

Synapt2_38593b 17 (0.364) Cm (17-3:4)
1: TOF MS ES+
2.02e6
Compound 10
310.1773
180.1135
292.1656
311.1798
181.1166
316.1852
%
m/z

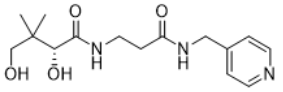

Compound 10

Elemental Composition Report

Page 1

Single Mass Analysis

Tolerance = 5.0 PPM / DBE: min = -1.5, max = 50.0
Element prediction: Off
Number of isotope peaks used for i-FIT = 3

Monoisotopic Mass, Even Electron Ions
162 formula(e) evaluated with 1 results within limits (up to 50 best isotopic matches for each mass)
Elements Used:
C: 0-50 H: 0-80 N: 0-5 O: 0-6

Order# 22631 Meyers Lab djm-14-25H-01
Synapt2_38593b 17 (0.364) Cm (17-3:4)

MSL, SCS, UIUC

SYNAPTG2-Si#UGA354
1: TOF MS ES+
2.02e+006

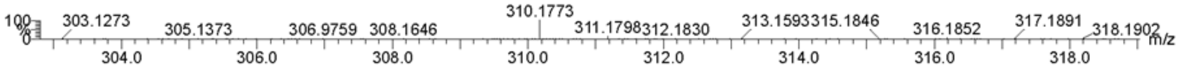

Minimum:				-1.5				
Maximum:		5.0	5.0	50.0				
Mass	Calc. Mass	mDa	PPM	DBE	i-FIT	Norm	Conf(%)	Formula
310.1773	310.1767	0.6	1.9	5.5	188.8	n/a	n/a	C15 H24 N3 O4

^{1}H NMR of **11**

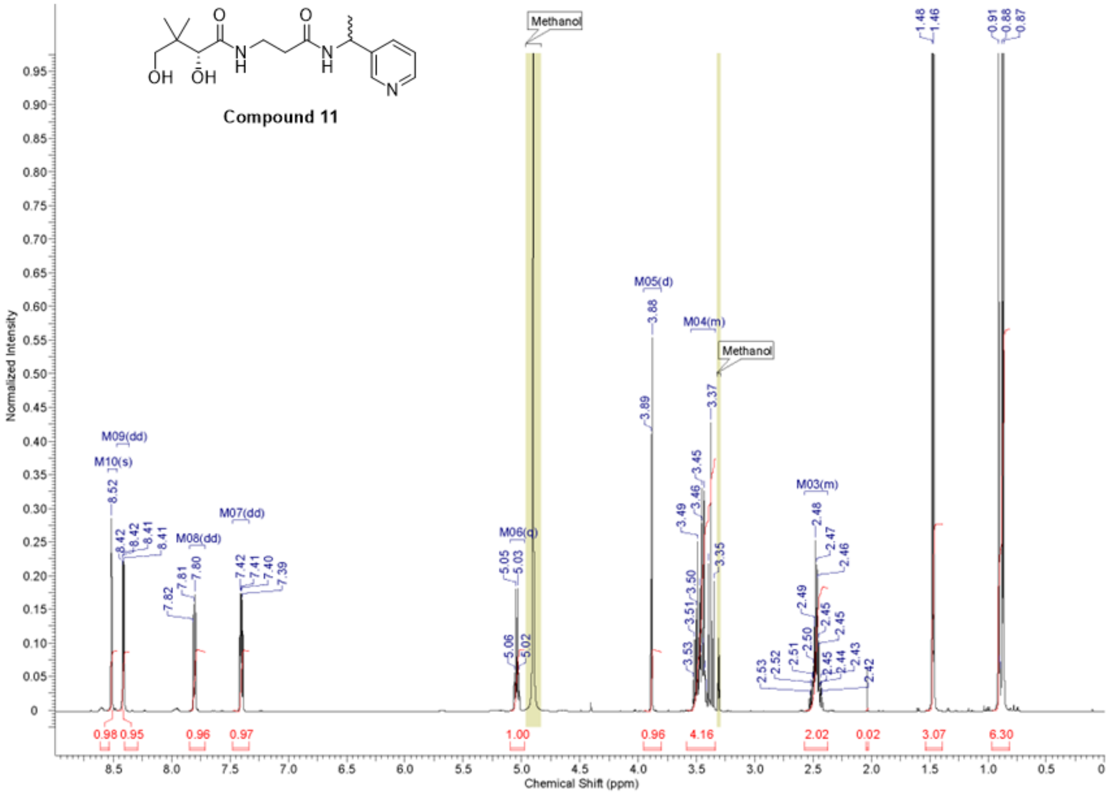
Compound 11
Methanol
Normalized Intensity
Chemical Shift (ppm)
M10(s)
M09(dd)
M08(dd)
M07(dd)
M06(q)
M05(d)
M04(m)
M03(m)

^{13}C NMR of **11**

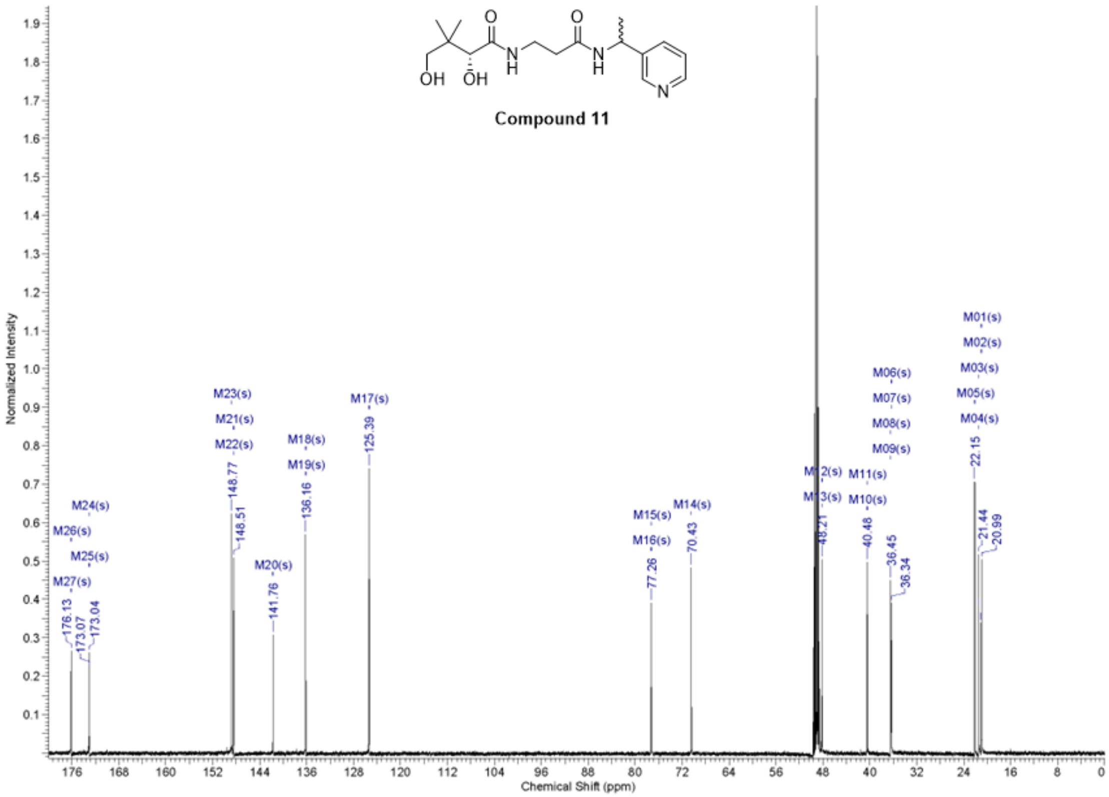
Compound 11
Normalized Intensity
Chemical Shift (ppm)
176.13
173.07
173.04
148.77
148.51
141.76
136.16
125.39
77.26
70.43
48.21
40.48
36.45
36.34
22.15
21.44
20.99

HRMS of **11**

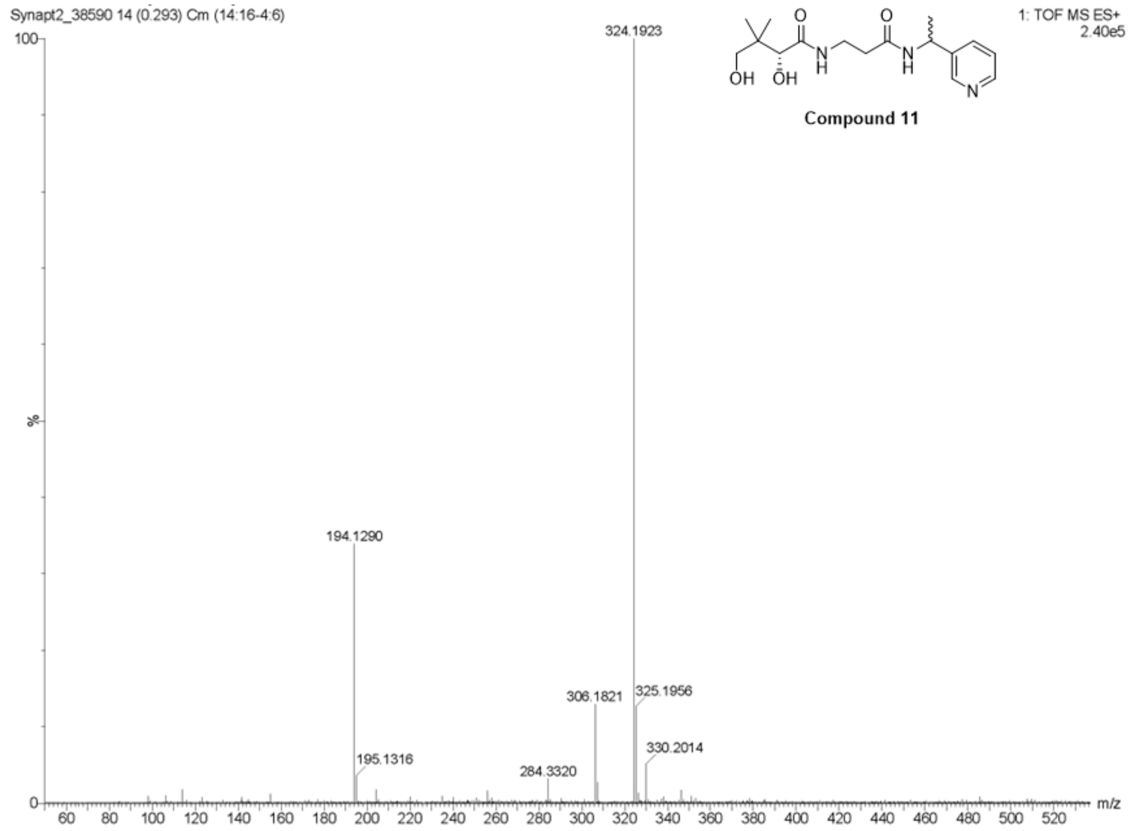

Synapt2_38590 14 (0.293) Cm (14:16-4:6)
1: TOF MS ES+
2.40e5
100
%
0
324.1923
194.1290
306.1821
325.1956
330.2014
195.1316
284.3320
m/z
60
80
100
120
140
160
180
200
220
240
260
280
300
320
340
360
380
400
420
440
460
480
500
520
OH
OH
O
O
N
H
N
H
N
Compound 11

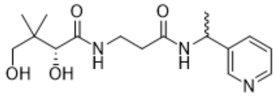

Compound 11

Elemental Composition Report

Page 1

Single Mass Analysis

Tolerance = 5.0 PPM / DBE: min = -1.5, max = 50.0
Element prediction: Off
Number of isotope peaks used for i-FIT = 3

Monoisotopic Mass, Even Electron Ions
364 formula(e) evaluated with 1 results within limits (up to 50 best isotopic matches for each mass)
Elements Used:
C: 0-50 H: 0-80 N: 0-5 O: 0-7 Na: 0-1

Order# 22626 Meyers Lab djm-14-25E-01 MSL, SCS, UIUC SYNAPTG2-Si#UGA354
Synapt2_38590 14 (0.293) Cm (14:16-4:6) 1: TOF MS ES+ 2.40e+005

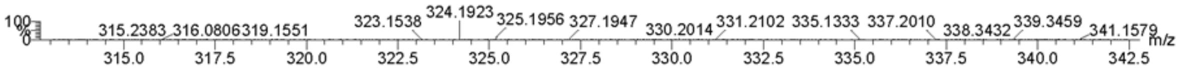

Minimum:				-1.5				
Maximum:		5.0	5.0	50.0				
Mass	Calc. Mass	mDa	PPM	DBE	i-FIT	Norm	Conf(%)	Formula
324.1923	324.1923	0.0	0.0	5.5	330.0	n/a	n/a	C16 H26 N3 O4

^{1}H NMR of **12**

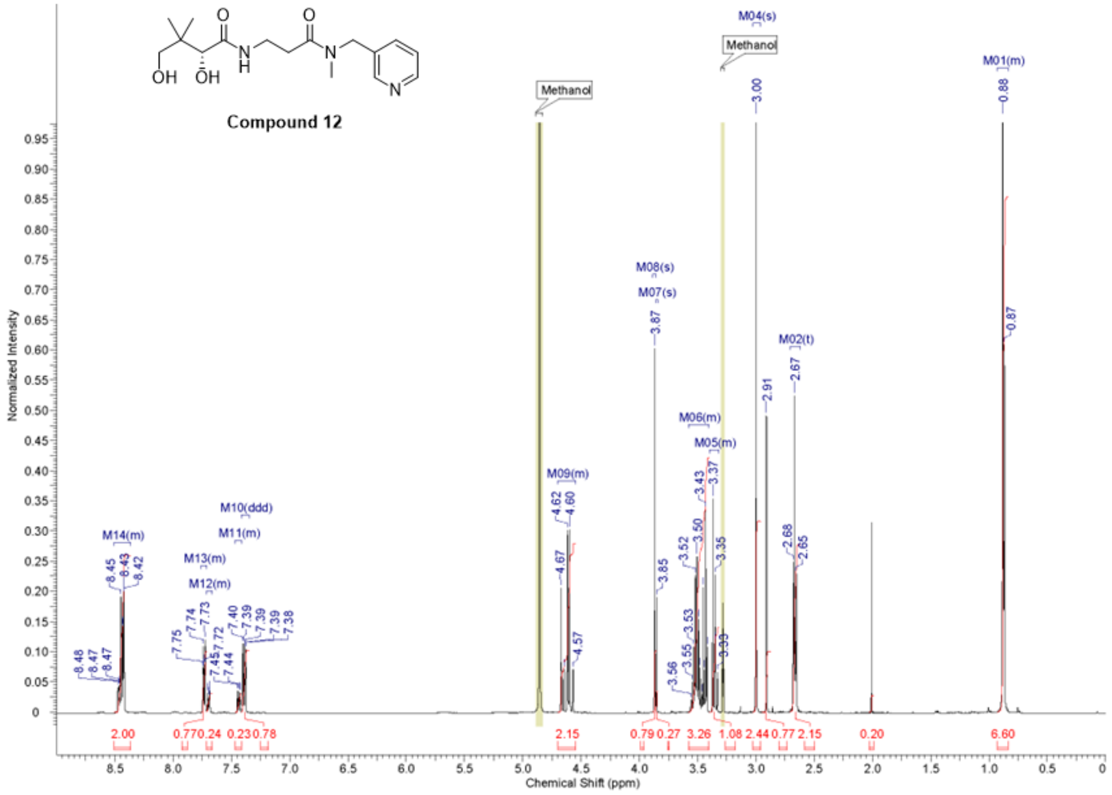
Compound 12
Methanol
Methanol
M01(m)
M02(t)
M04(s)
M05(m)
M06(m)
M07(s)
M08(s)
M09(m)
M10(ddd)
M11(m)
M12(m)
M13(m)
M14(m)
Normalized Intensity
Chemical Shift (ppm)

^{13}C NMR of **12**

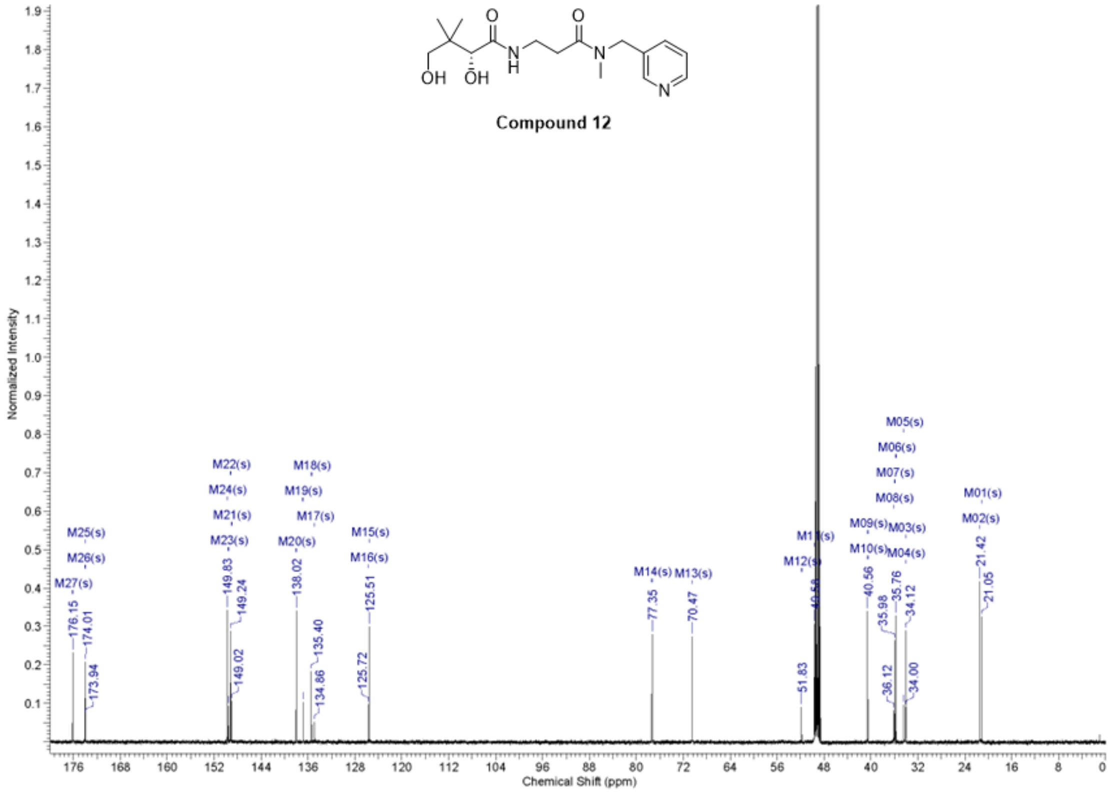
Compound 12
Normalized Intensity
Chemical Shift (ppm)
176.15
174.01
173.94
149.83
149.24
149.02
138.02
135.40
134.86
125.72
125.51
77.35
70.47
51.83
40.56
36.12
35.98
35.76
34.12
34.00
21.42
21.05

HRMS of **12**

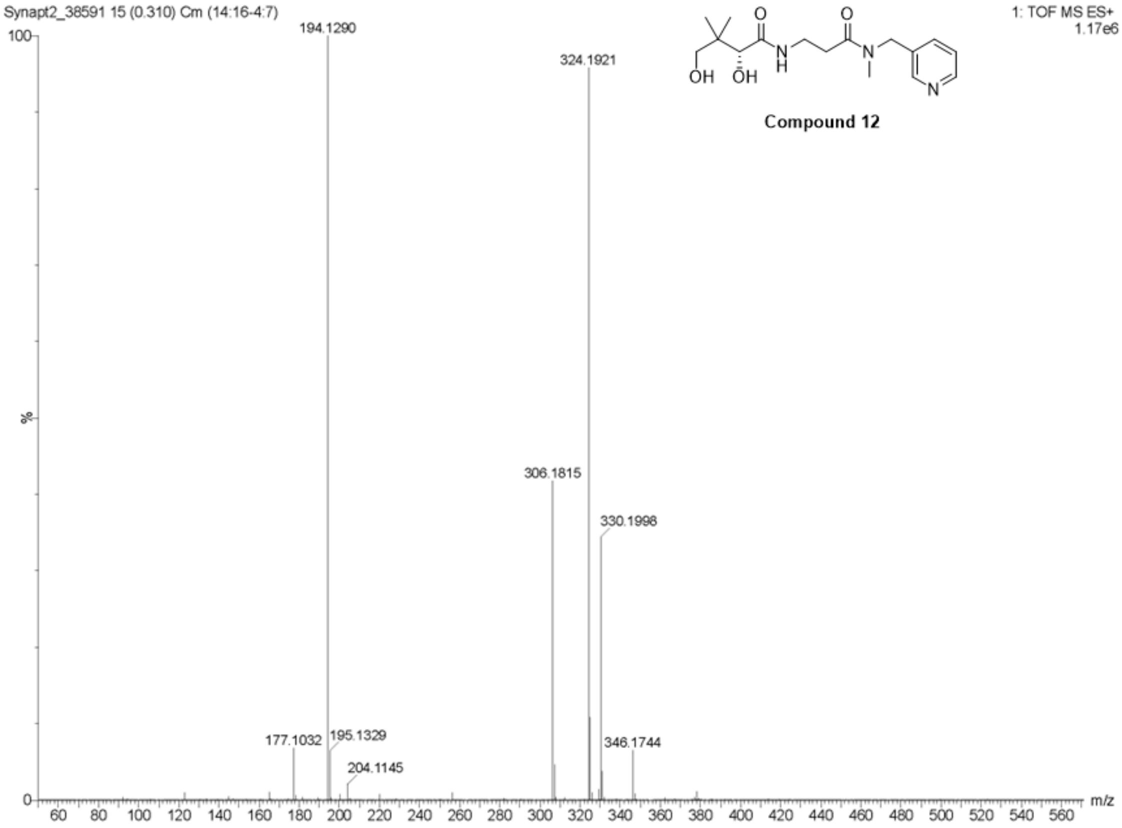
Synapt2_38591 15 (0.310) Cm (14:16-4:7)
1: TOF MS ES+
1.17e6
Compound 12
194.1290
324.1921
306.1815
330.1998
177.1032
195.1329
204.1145
346.1744
%
m/z

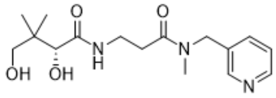

Compound 12

Elemental Composition Report **Page 1**

Single Mass Analysis
Tolerance = 5.0 PPM / DBE: min = -1.5, max = 50.0
Element prediction: Off
Number of isotope peaks used for i-FIT = 3

Monoisotopic Mass, Even Electron Ions
364 formula(e) evaluated with 1 results within limits (up to 50 best isotopic matches for each mass)
Elements Used:
C: 0-50 H: 0-80 N: 0-5 O: 0-7 Na: 0-1

Order# 22627 Meyers Lab djm-14-25F-01 MSL, SCS, UIUC SYNAPTG2-Si#UGA354
Synapt2_38591 15 (0.310) Cm (14:16-4:7) 1: TOF MS ES+
1.12e+006

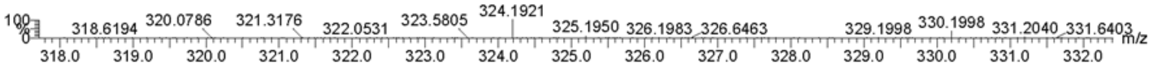

Minimum:				-1.5				
Maximum:		5.0	5.0	50.0				
Mass	Calc. Mass	mDa	PPM	DBE	i-FIT	Norm	Conf(%)	Formula
324.1921	324.1923	-0.2	-0.6	5.5	218.8	n/a	n/a	C16 H26 N3 O4

^{1}H NMR of **13**

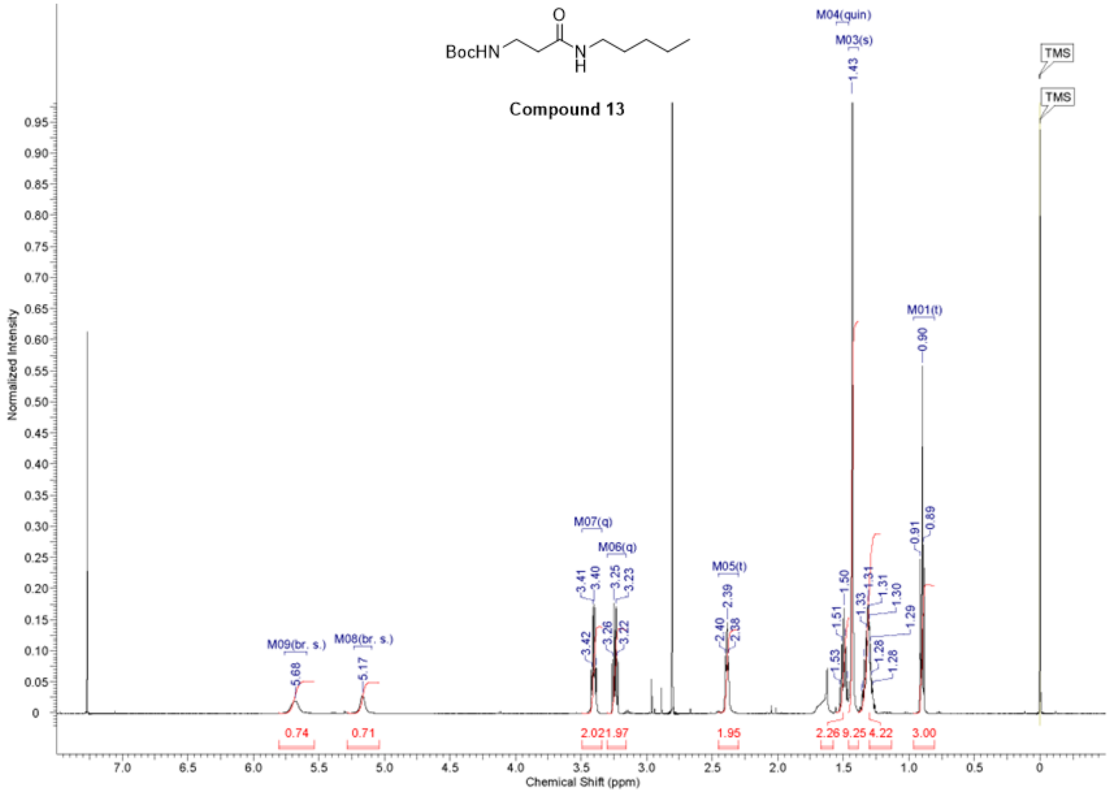

BocHN
O
N
H
Compound 13
M04(quin)
M03(s)
TMS
TMS
M01(t)
M07(q)
M06(q)
M05(t)
M09(br. s.)
M08(br. s.)
Normalized Intensity
Chemical Shift (ppm)
0.74
0.71
2.02
1.97
1.95
2.26
9.25
4.22
3.00

^{1}H NMR of **14**

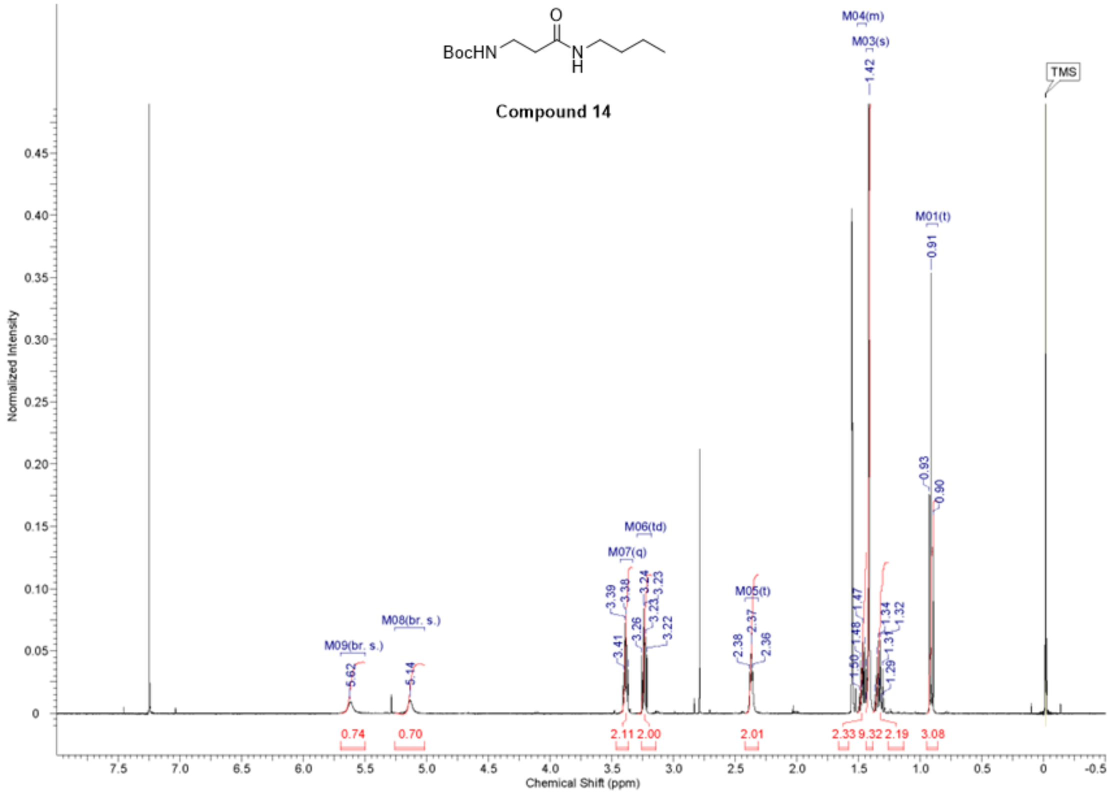
BocHN
O
N
H
Compound 14
M04(m)
M03(s)
1.42
TMS
M01(t)
0.91
0.93
0.90
M06(td)
M07(q)
3.39
3.38
3.24
3.23
3.23
3.22
3.26
3.41
M05(t)
2.38
2.37
2.36
M08(br. s.)
5.14
M09(br. s.)
5.62
1.47
1.48
1.50
1.34
1.32
1.31
1.29
0.74
0.70
2.11
2.00
2.01
2.33
9.32
2.19
3.08
Normalized Intensity
Chemical Shift (ppm)

^{1}H NMR of **15**

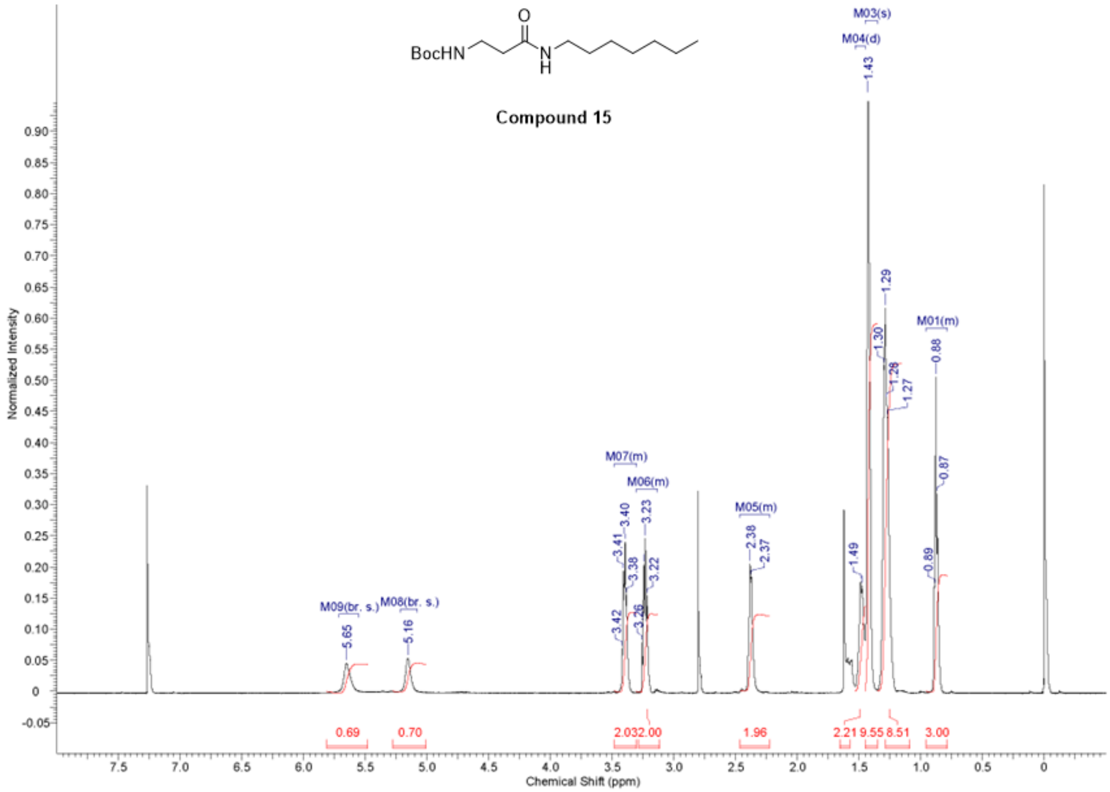
BocHN
O
N
H
Compound 15
M03(s)
M04(d)
M01(m)
M07(m)
M06(m)
M05(m)
M09(br. s.)
M08(br. s.)
0.69
0.70
2.03
2.00
1.96
2.21
9.55
8.51
3.00
Normalized Intensity
Chemical Shift (ppm)

^{1}H NMR of **16**

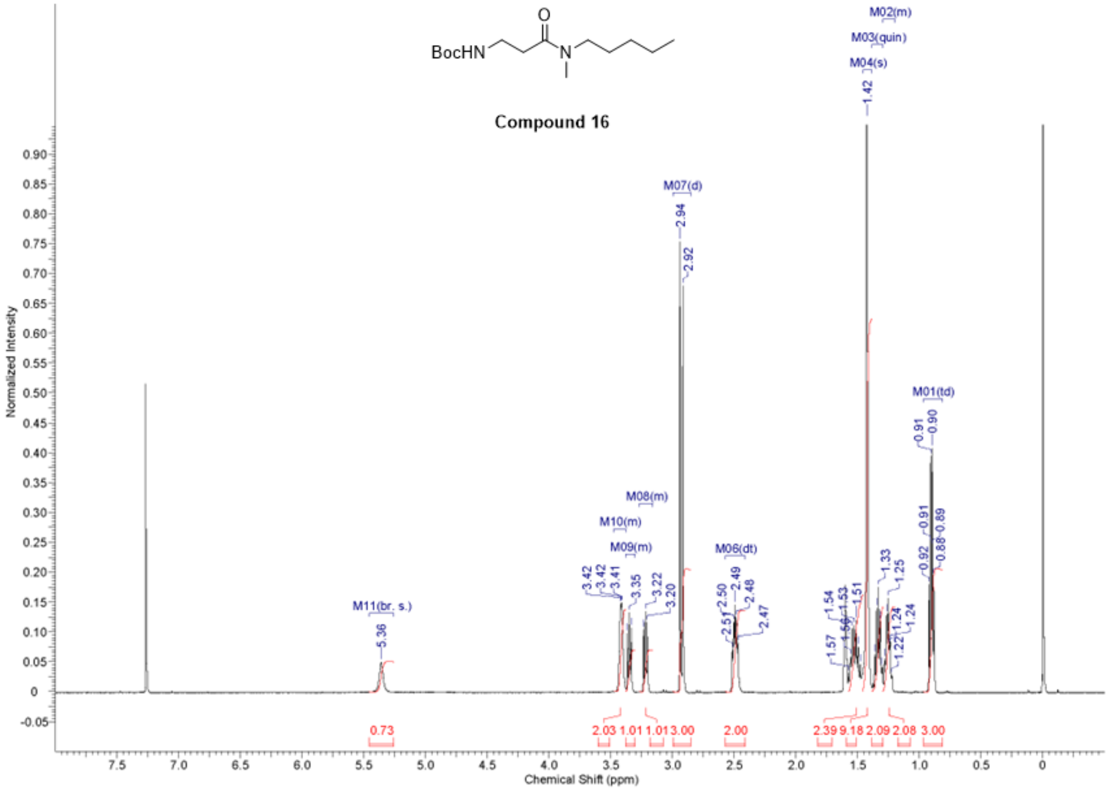
BocHN
O
N
Compound 16
Normalized Intensity
Chemical Shift (ppm)
M01(td)
M02(m)
M03(quin)
M04(s)
M06(dt)
M07(d)
M08(m)
M09(m)
M10(m)
M11(br. s.)
0.73
2.03
1.01
1.01
3.00
2.00
2.39
9.18
2.09
2.08
3.00

^{1}H NMR of **17**

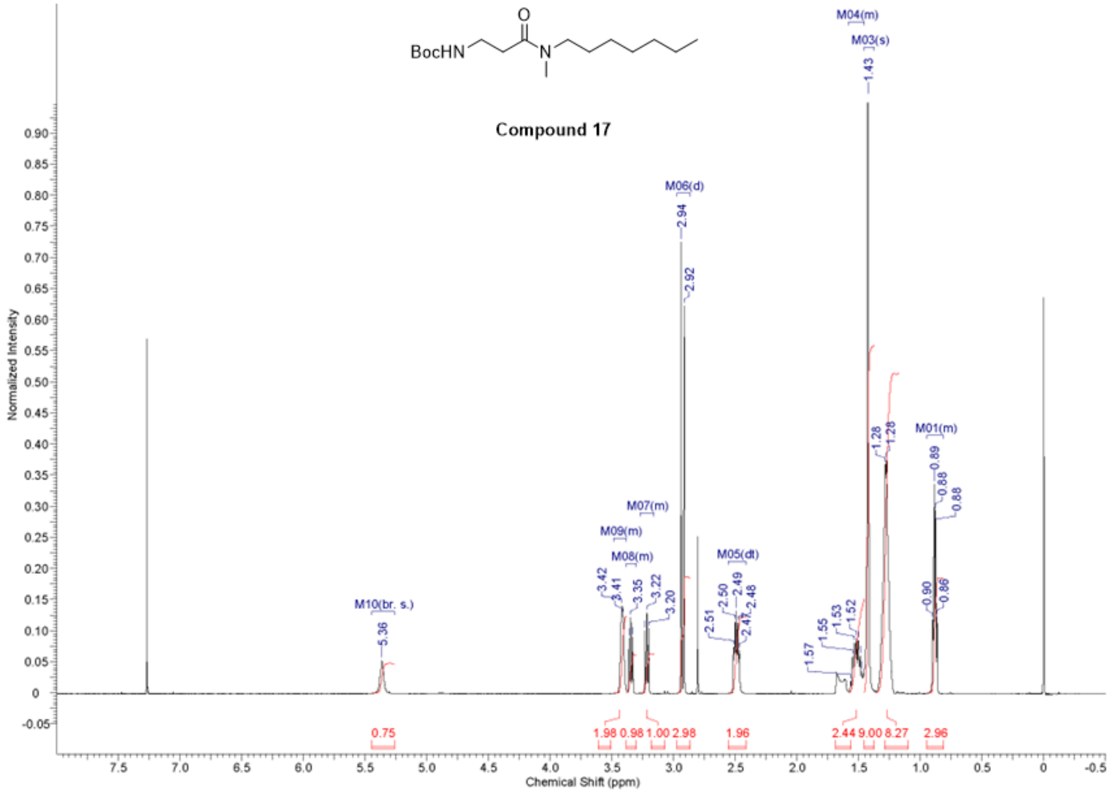

BocHN
O
N
Compound 17
Normalized Intensity
Chemical Shift (ppm)
M10(br. s.)
M09(m)
M08(m)
M07(m)
M06(d)
M05(dt)
M04(m)
M03(s)
M01(m)
0.75
1.98
0.98
1.00
2.98
1.96
2.44
9.00
8.27
2.96

^{1}H NMR of **18**

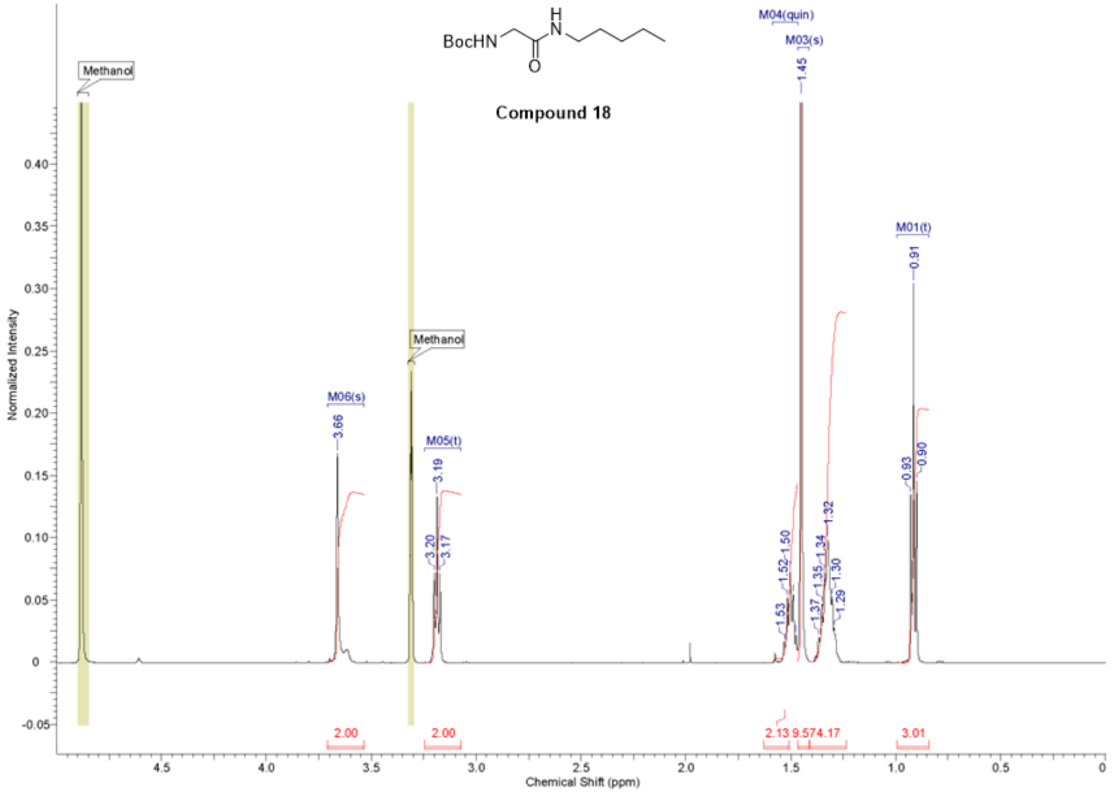
BocHN
Compound 18
Methanol
Methanol
M06(s)
M05(t)
M04(quin)
M03(s)
M01(t)
Normalized Intensity
Chemical Shift (ppm)

^{1}H NMR of **19**

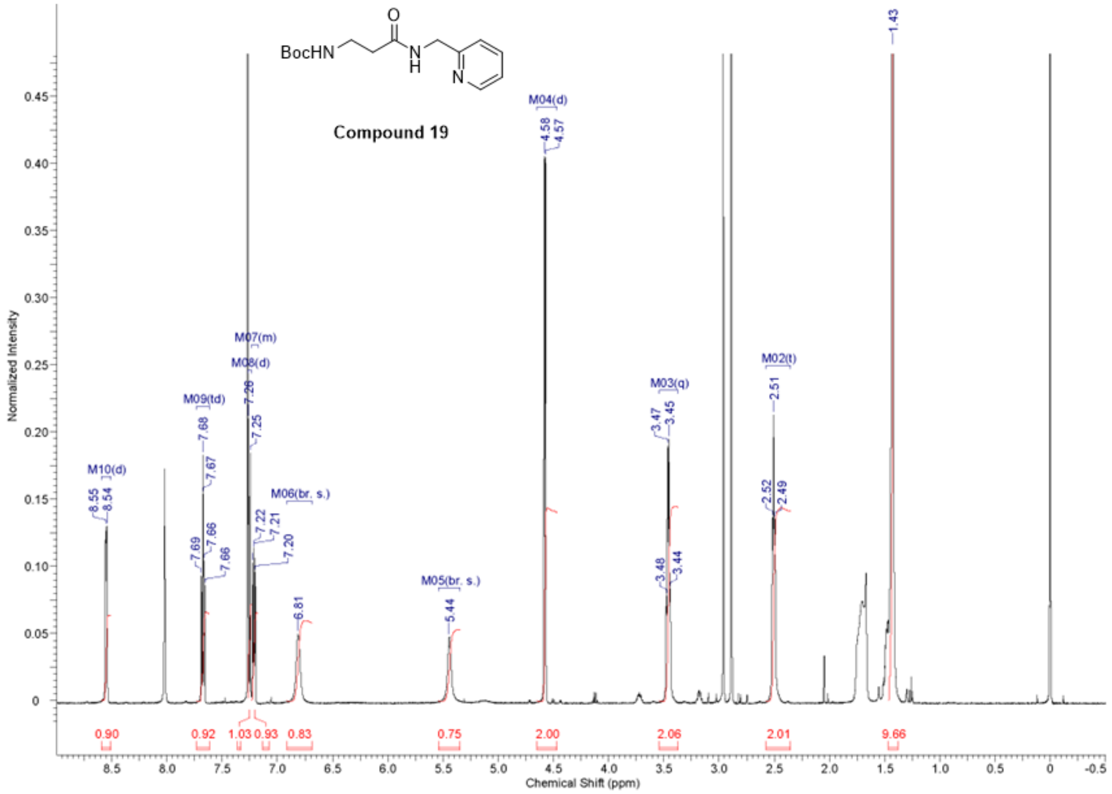
BocHN
O
N
H
N
Compound 19
M04(d)
4.58
4.57
M10(d)
8.55
8.54
M09(td)
7.68
7.67
7.69
7.66
7.66
M07(m)
M08(d)
7.28
7.25
7.22
7.21
7.20
M06(br. s.)
6.81
M05(br. s.)
5.44
M03(q)
3.47
3.45
3.48
3.44
M02(t)
2.51
2.52
2.49
1.43
0.90
0.92
1.03
0.93
0.83
0.75
2.00
2.06
2.01
9.66
Normalized Intensity
Chemical Shift (ppm)

^{1}H NMR of **20**

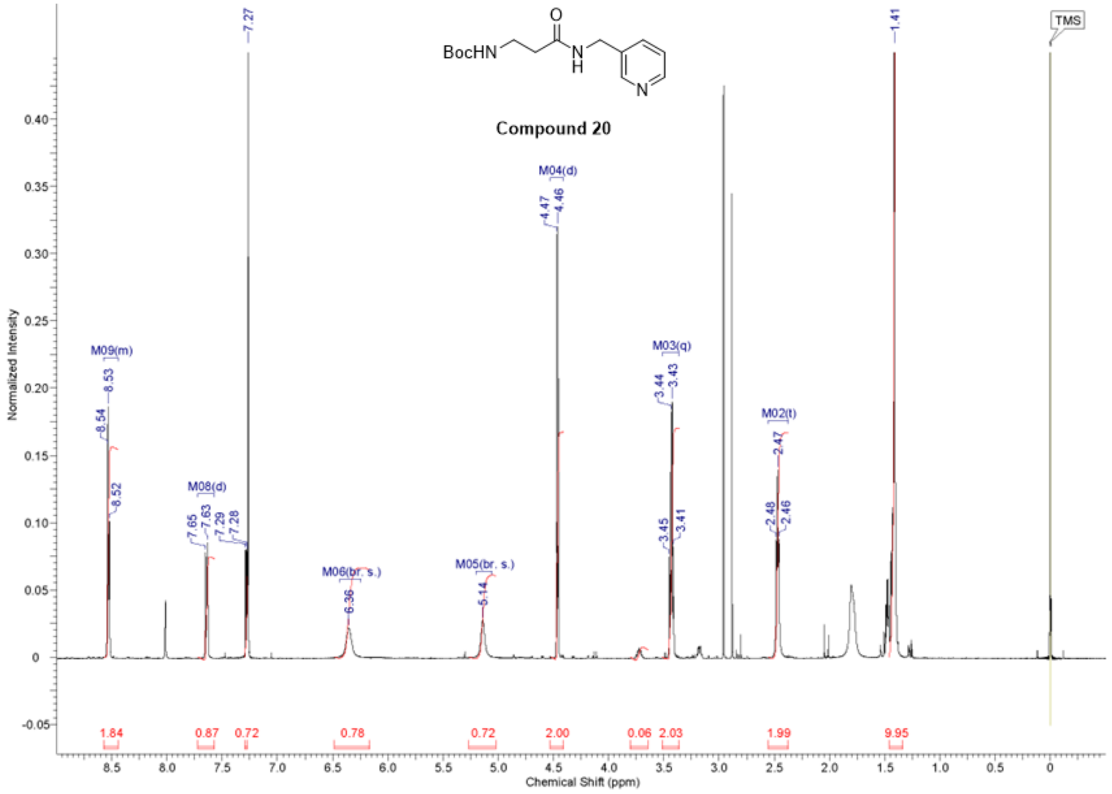

BocHN
O
N
H
N
Compound 20
TMS
M09(m)
M08(d)
M06(br. s.)
M05(br. s.)
M04(d)
M03(q)
M02(t)
1.84
0.87
0.72
0.78
0.72
2.00
0.06
2.03
1.99
9.95
Normalized Intensity
Chemical Shift (ppm)

^{1}H NMR of **21**

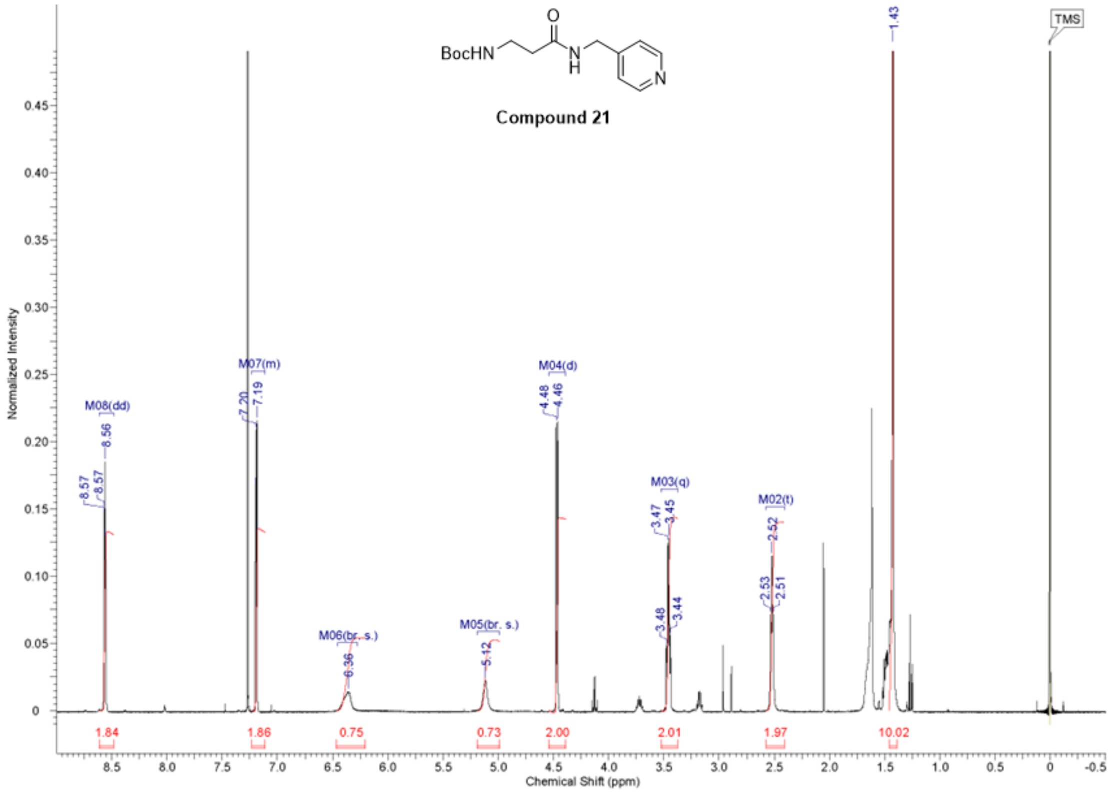
BocHN
Compound 21
TMS
1.43
M08(dd)
8.57
8.57
8.56
M07(m)
7.20
7.19
M06(br. s.)
6.36
M05(br. s.)
5.12
M04(d)
4.48
4.46
M03(q)
3.47
3.45
3.48
3.44
M02(t)
2.52
2.53
2.51
1.84
1.86
0.75
0.73
2.00
2.01
1.97
10.02
Normalized Intensity
Chemical Shift (ppm)

^{1}H NMR of **22**

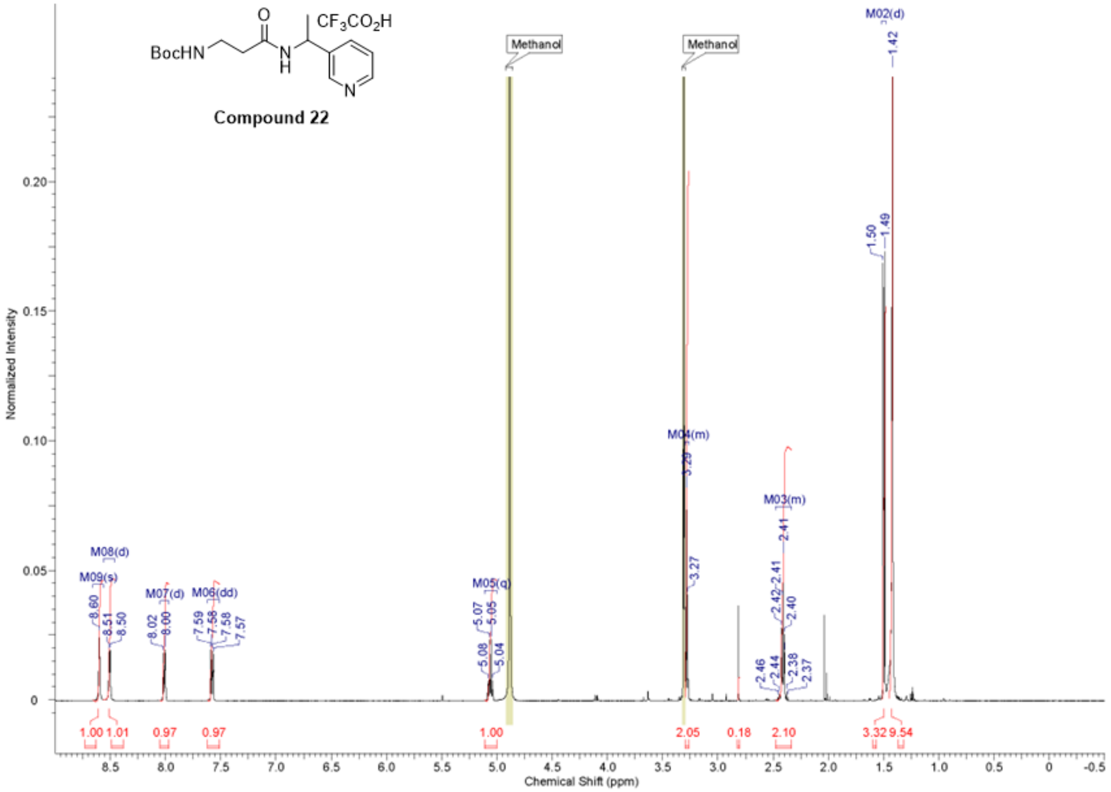

BocHN
CF3CO2H
N
H
O
Compound 22
Methanol
Methanol
M02(d)
M03(m)
M04(m)
M05(q)
M06(dd)
M07(d)
M08(d)
M09(s)
1.00
1.01
0.97
0.97
1.00
2.05
0.18
2.10
3.32
9.54
Normalized Intensity
Chemical Shift (ppm)

^{1}H NMR of **23**

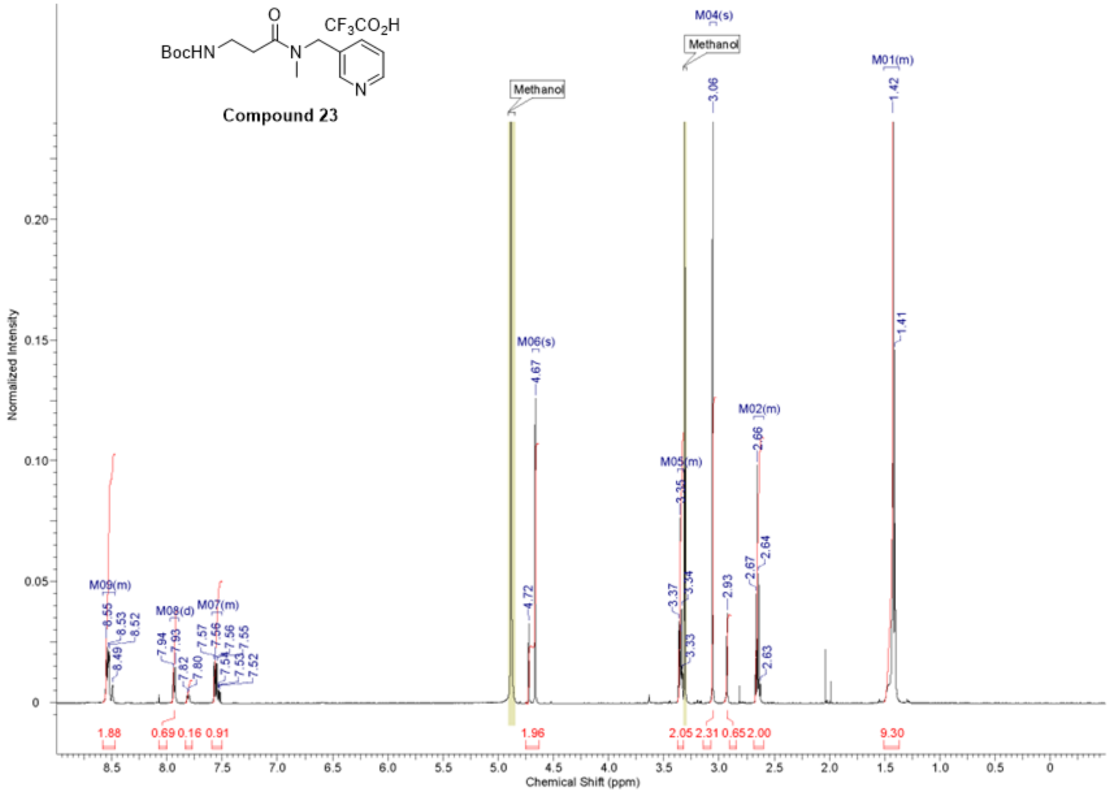

BocHN
O
N
CF3CO2H
N
Compound 23
Methanol
Methanol
M04(s)
M01(m)
M06(s)
M05(m)
M02(m)
M09(m)
M08(d)
M07(m)
3.06
1.42
1.41
4.67
4.72
3.35
3.37
3.34
3.33
2.93
2.66
2.67
2.64
2.63
8.55
8.53
8.52
8.49
7.94
7.93
7.82
7.80
7.57
7.56
7.56
7.55
7.54
7.53
7.52
1.88
0.69
0.16
0.91
1.96
2.05
2.31
0.65
2.00
9.30
Normalized Intensity
0.20
0.15
0.10
0.05
0
8.5
8.0
7.5
7.0
6.5
6.0
5.5
5.0
4.5
4.0
3.5
3.0
2.5
2.0
1.5
1.0
0.5
0
Chemical Shift (ppm)

^{1}H NMR of **24**

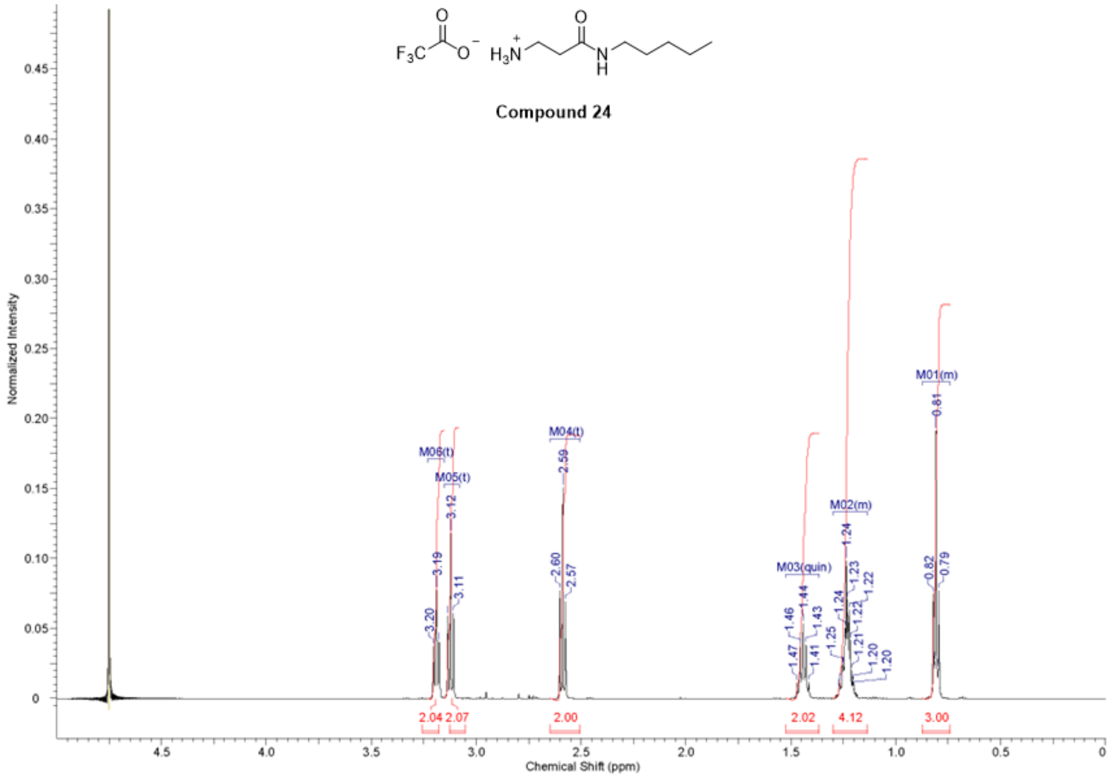
Compound 24
Normalized Intensity
Chemical Shift (ppm)
M06(t)
M05(t)
M04(t)
M03(quin)
M02(m)
M01(m)
2.04
2.07
2.00
2.02
4.12
3.00

^{13}C NMR of **24**

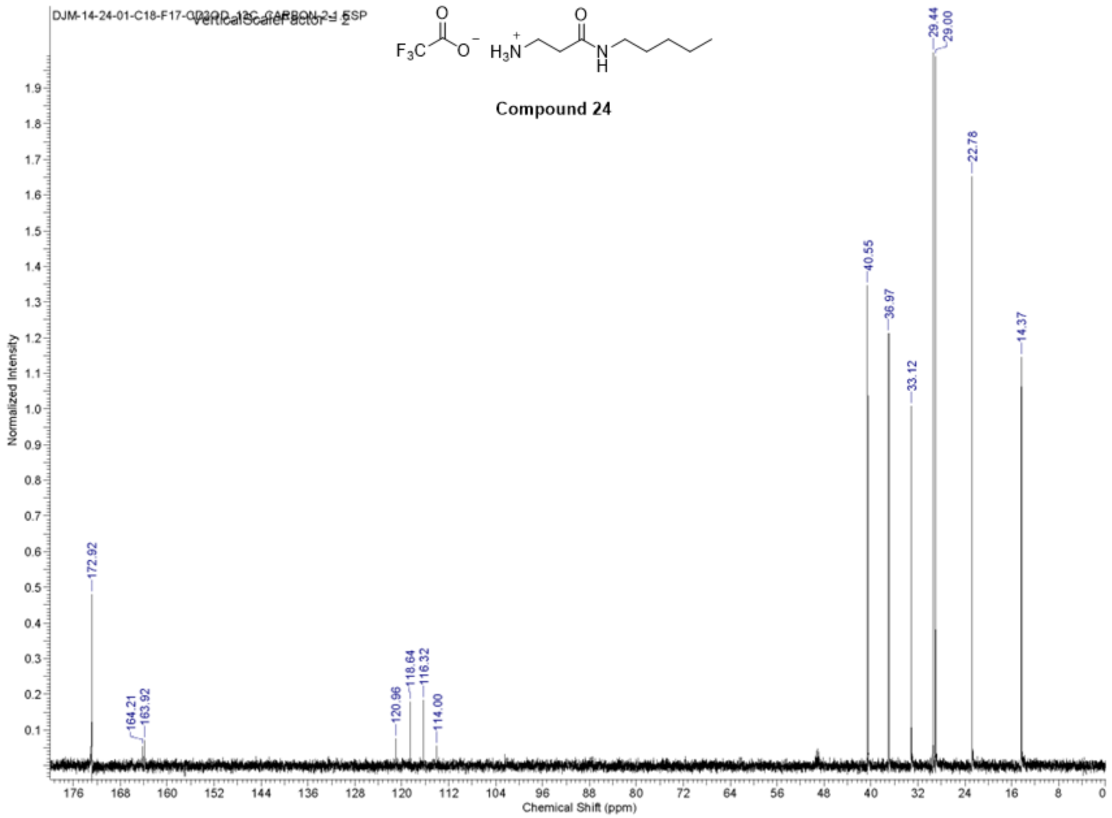

Compound 24
172.92
164.21
163.92
120.96
118.64
116.32
114.00
40.55
36.97
33.12
29.44
29.00
22.78
14.37
Normalized Intensity
Chemical Shift (ppm)

^{1}H NMR of **25**

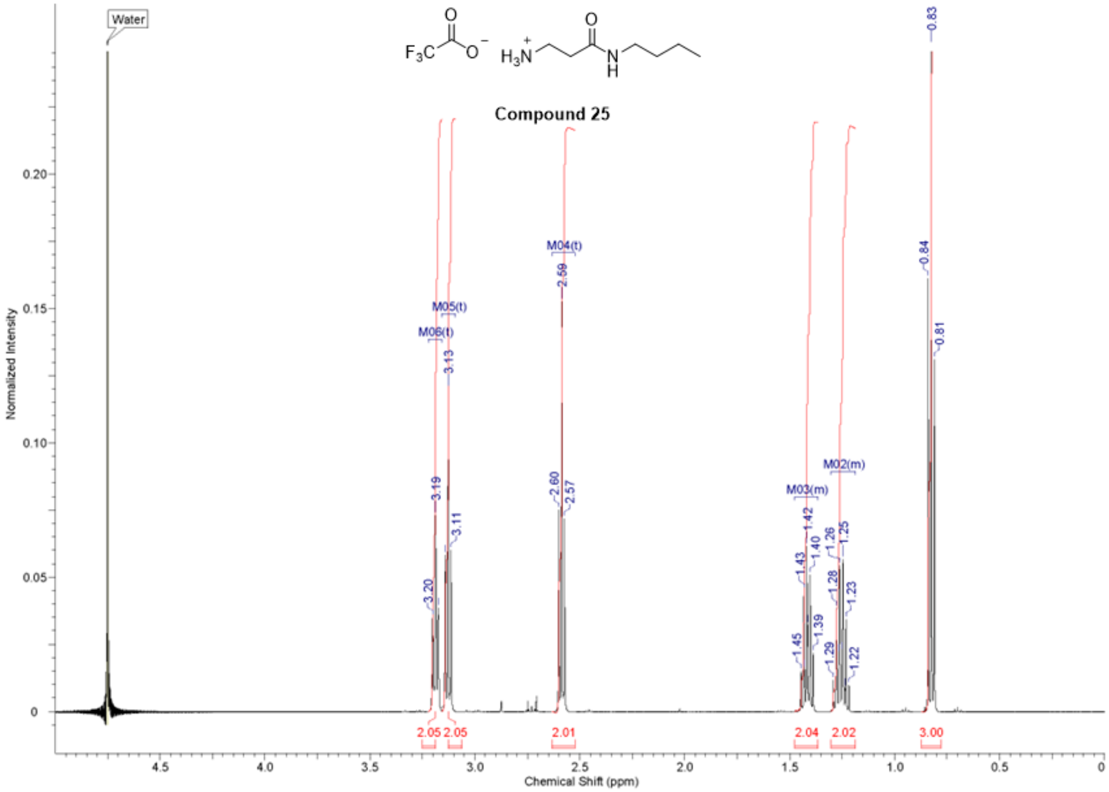
Compound 25
Water
Normalized Intensity
Chemical Shift (ppm)
M06(t)
M05(t)
M04(t)
M03(m)
M02(m)
2.05
2.05
2.01
2.04
2.02
3.00

^{13}C NMR of **25**

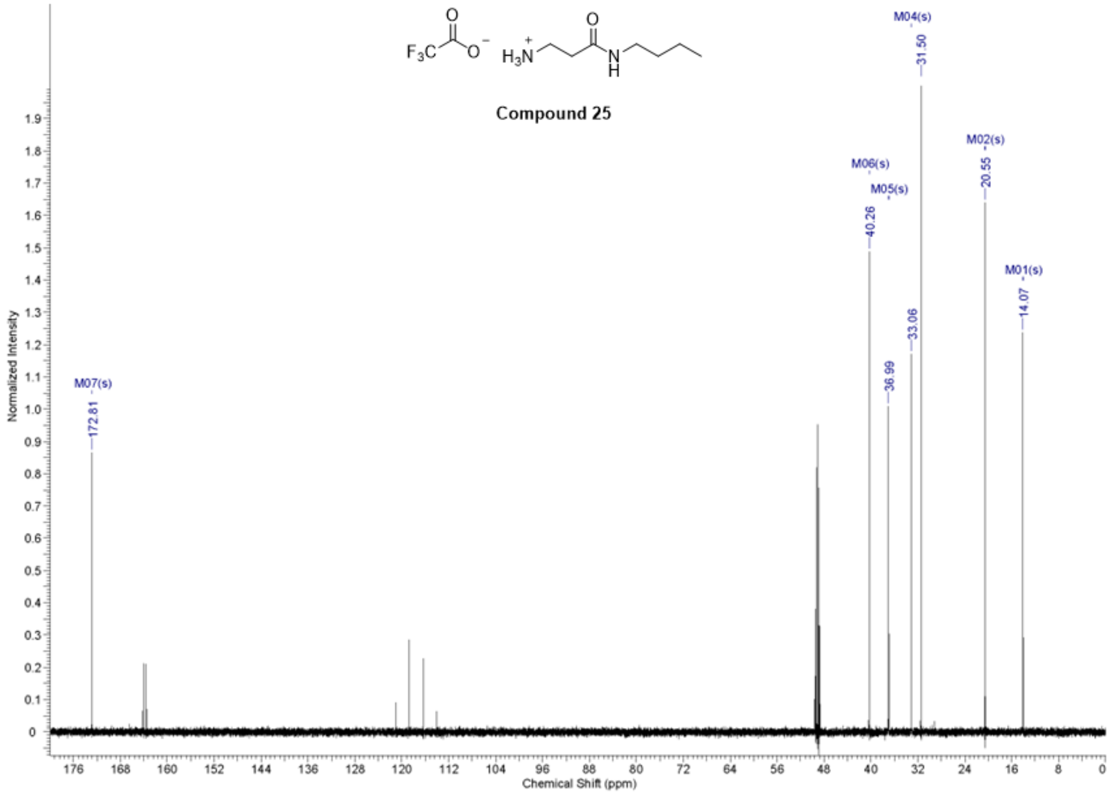

Compound 25
M07(s)
172.81
M06(s)
40.26
M05(s)
36.99
33.06
M04(s)
31.50
M02(s)
20.55
M01(s)
14.07
Normalized Intensity
Chemical Shift (ppm)

^{1}H NMR of **26**

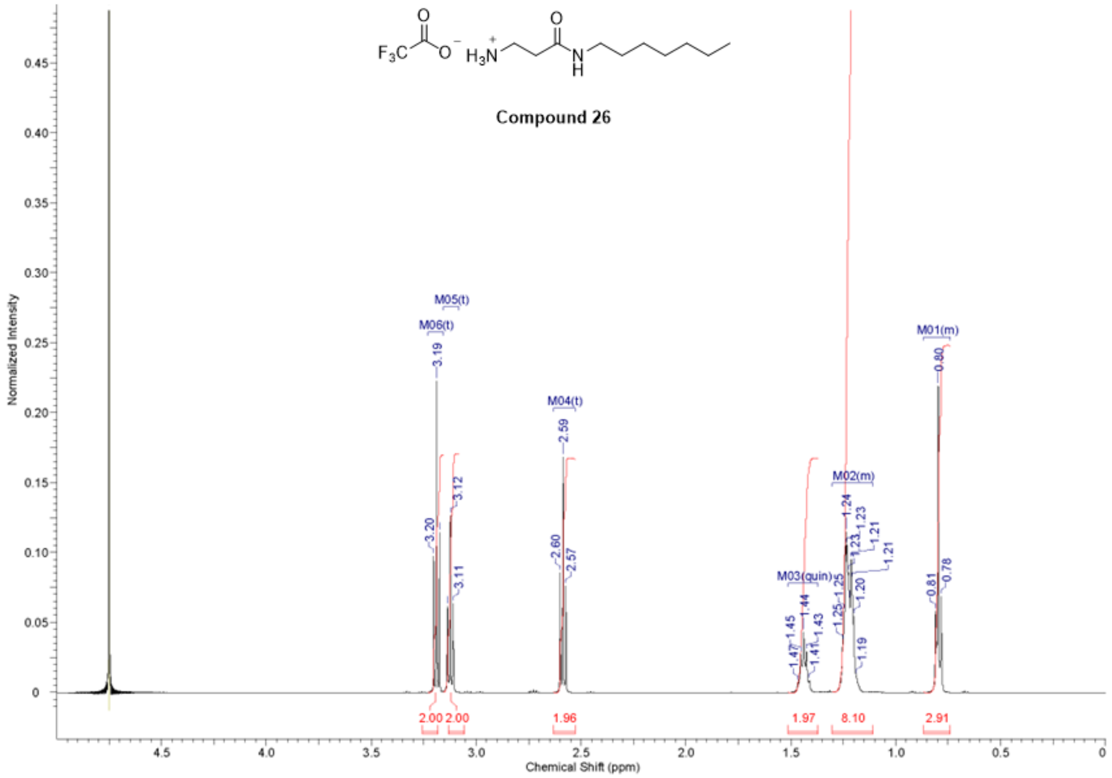

Compound 26
Normalized Intensity
Chemical Shift (ppm)
M06(t)
M05(t)
M04(t)
M03(quin)
M02(m)
M01(m)
2.00
2.00
1.96
1.97
8.10
2.91

^{13}C NMR of **26**

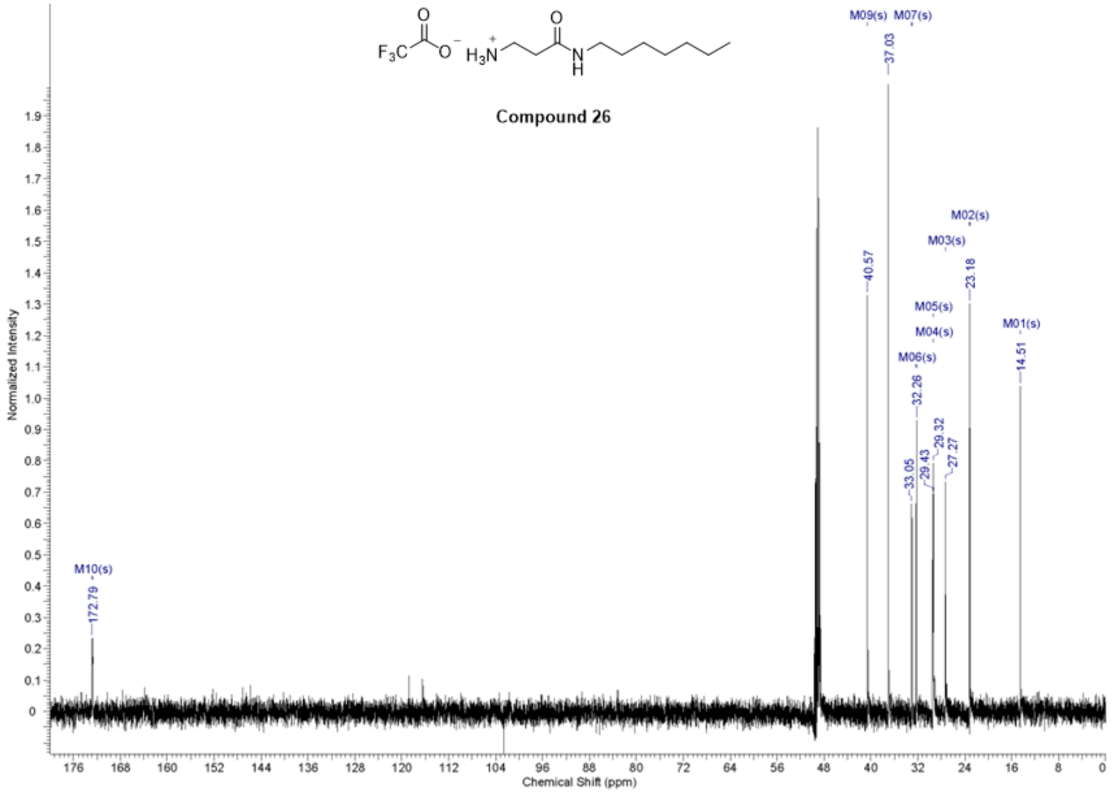

F3C O− H3N+ O N H
Compound 26
M09(s) M07(s)
37.03
40.57
M02(s)
M03(s)
23.18
M05(s)
M04(s)
M06(s)
32.26
33.05
29.43
29.32
27.27
M01(s)
14.51
M10(s)
172.79
Normalized Intensity
Chemical Shift (ppm)

^{1}H NMR of **27**

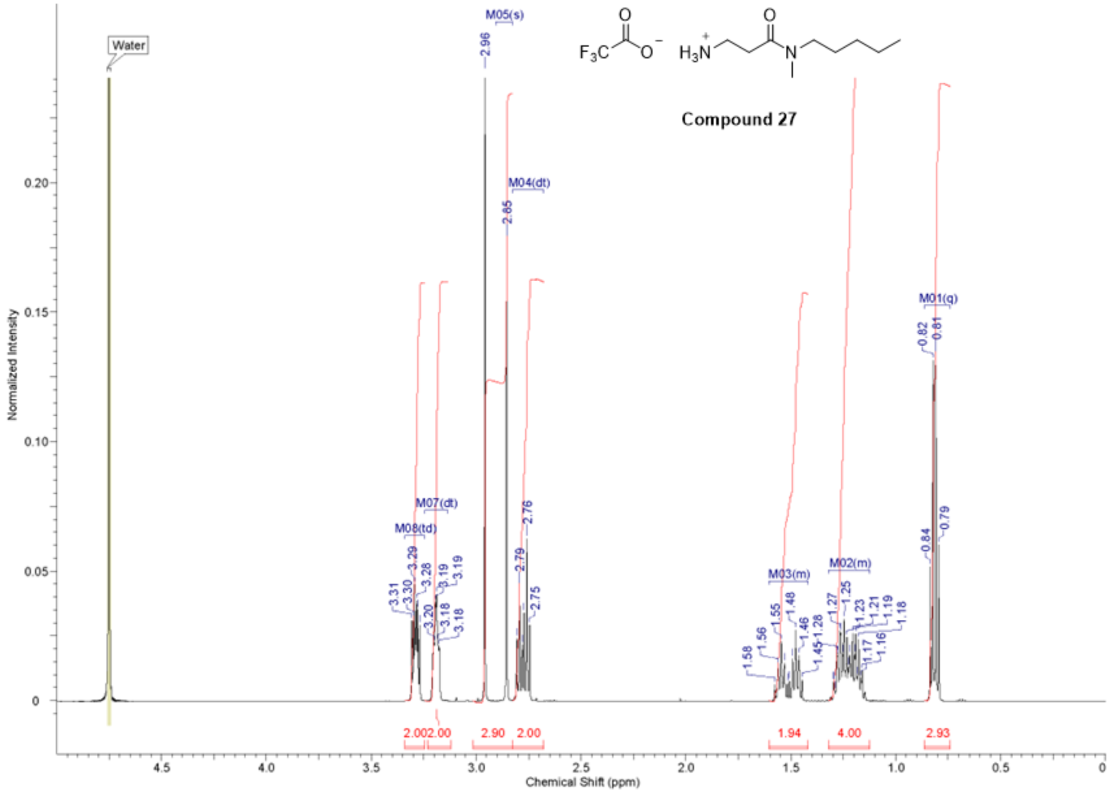

Compound 27
Water
M05(s)
M04(dt)
M07(dt)
M08(td)
M03(m)
M02(m)
M01(q)
2.00
2.00
2.90
2.00
1.94
4.00
2.93
Normalized Intensity
Chemical Shift (ppm)

^{13}C NMR of **27**

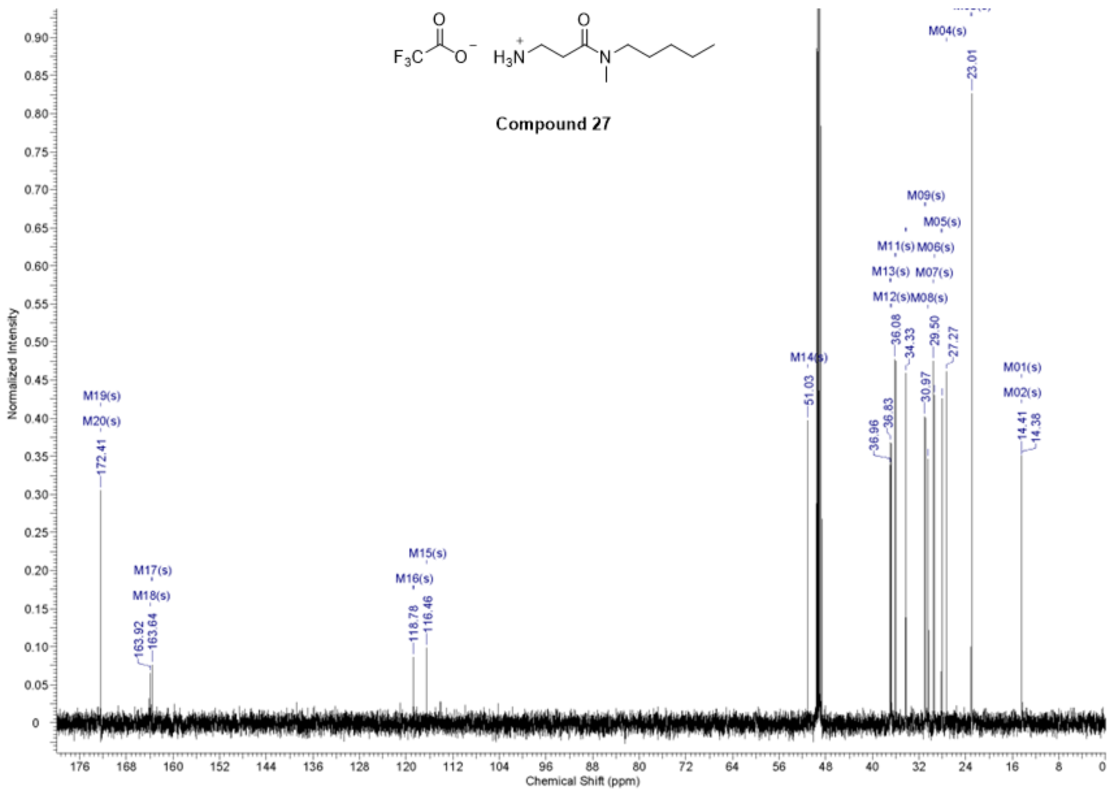
Compound 27
M19(s)
M20(s)
172.41
M17(s)
M18(s)
163.92
163.64
M15(s)
M16(s)
118.78
116.46
M14(s)
51.03
M09(s)
M05(s)
M11(s) M06(s)
M13(s) M07(s)
M12(s) M08(s)
36.96
36.83
36.08
34.33
30.97
29.50
27.27
M04(s)
23.01
M01(s)
M02(s)
14.41
14.38
Normalized Intensity
Chemical Shift (ppm)

^{1}H NMR of **28**

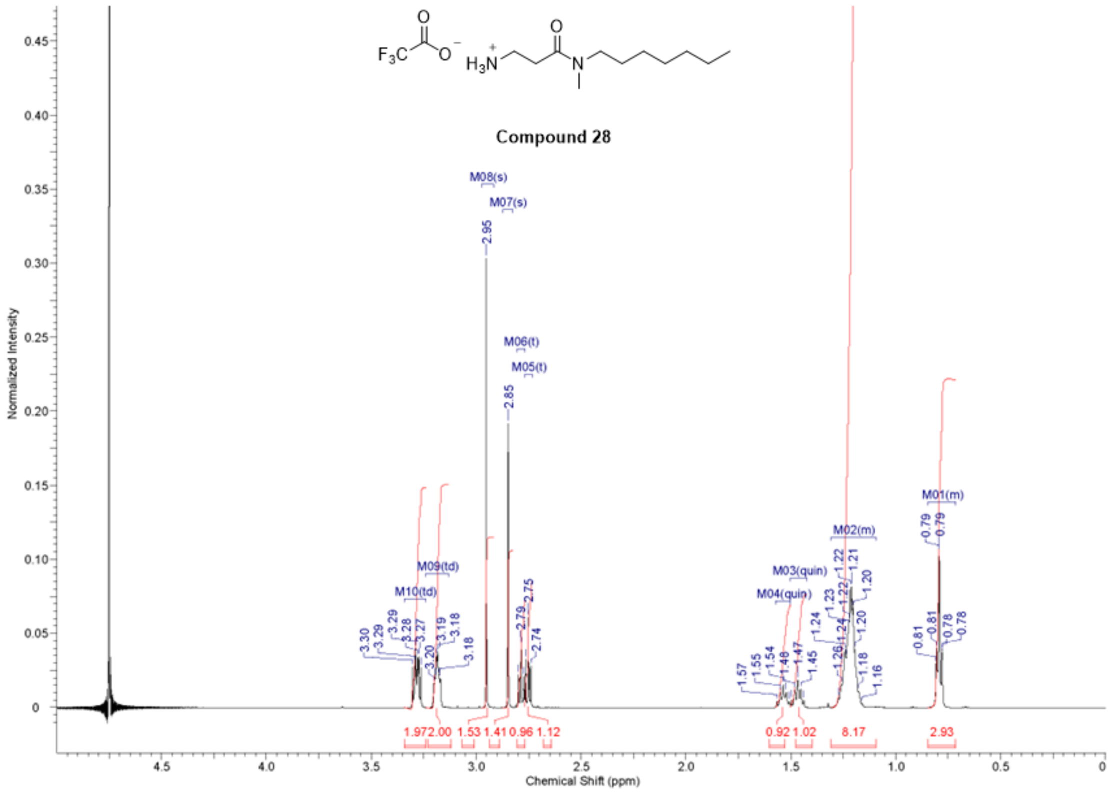

^{1}H NMR of **29**

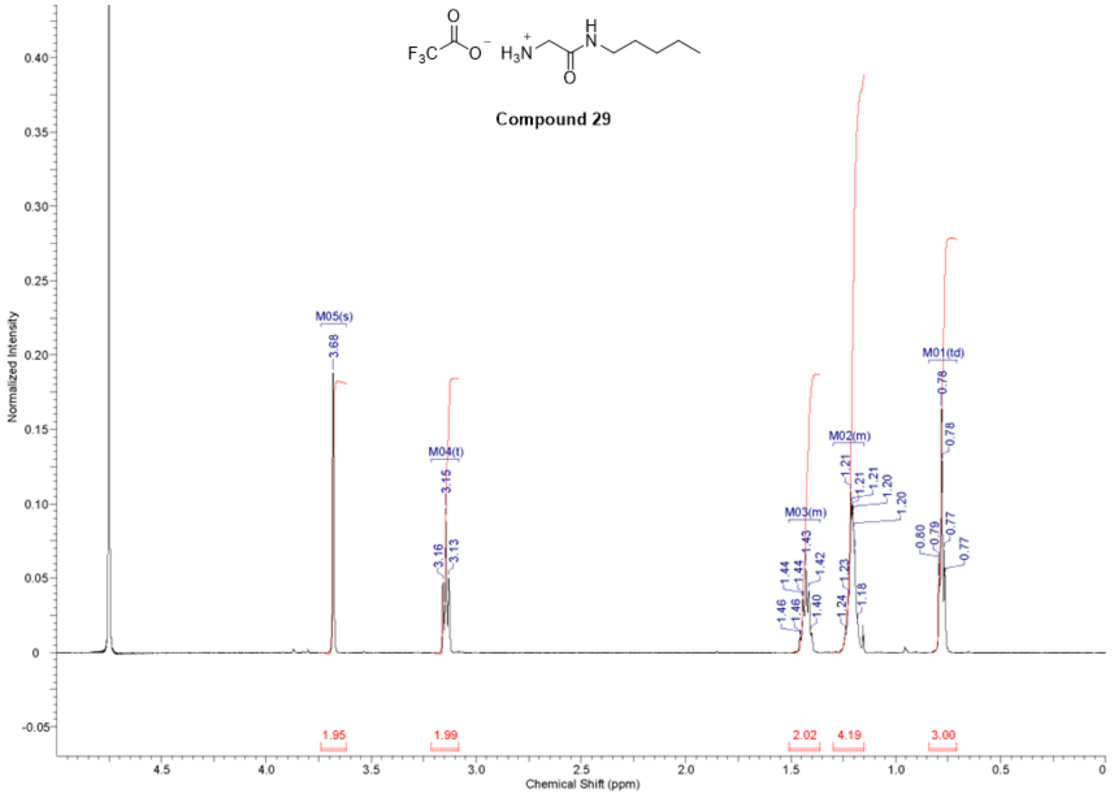

Compound 29
M05(s)
M04(t)
M03(m)
M02(m)
M01(td)
1.95
1.99
2.02
4.19
3.00
Normalized Intensity
Chemical Shift (ppm)

^{1}H NMR of **30**

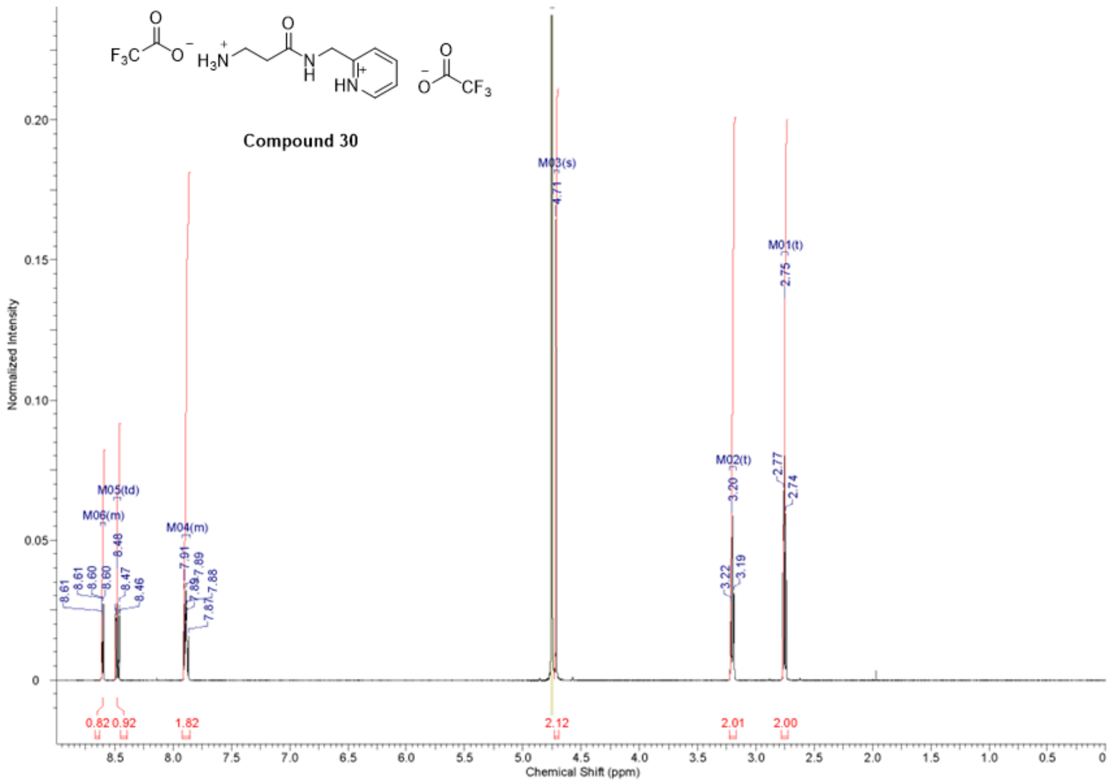

Compound 30
Normalized Intensity
Chemical Shift (ppm)
M01(t)
M02(t)
M03(s)
M04(m)
M05(td)
M06(m)
0.82
0.92
1.82
2.12
2.01
2.00

^{13}C NMR of **30**

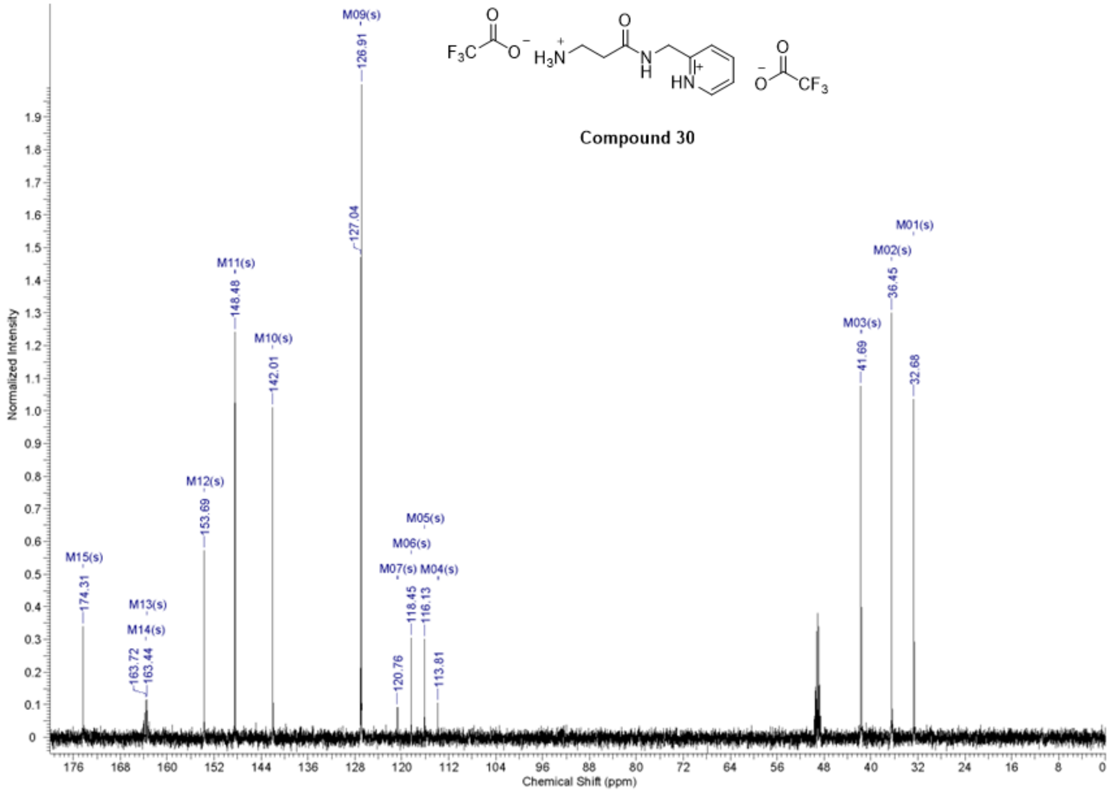

Compound 30
M09(s)
126.91
127.04
M11(s)
148.48
M10(s)
142.01
M12(s)
153.69
M15(s)
174.31
M13(s)
M14(s)
163.72
163.44
M05(s)
M06(s)
M07(s) M04(s)
118.45
116.13
120.76
113.81
M01(s)
M02(s)
36.45
M03(s)
41.69
32.68
Normalized Intensity
Chemical Shift (ppm)

^{1}H NMR of **31**

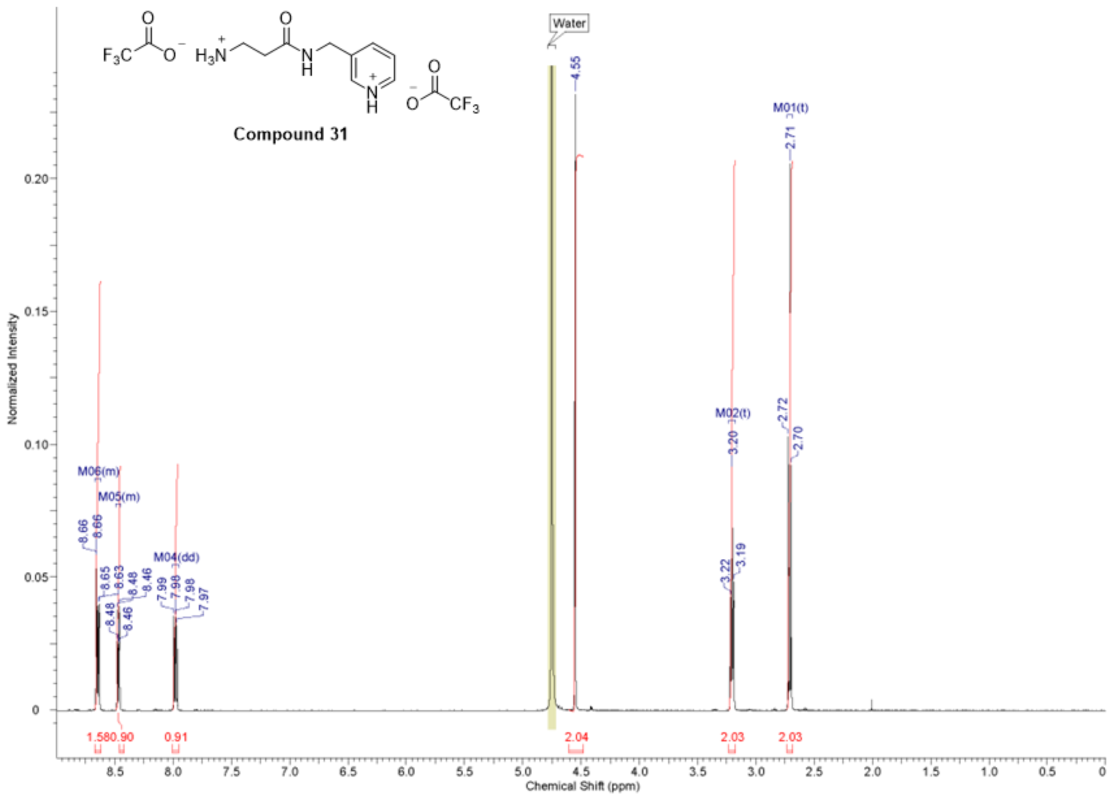
Compound 31
Water
Normalized Intensity
Chemical Shift (ppm)
M01(t)
M02(t)
M04(dd)
M05(m)
M06(m)

^{13}C NMR of **31**

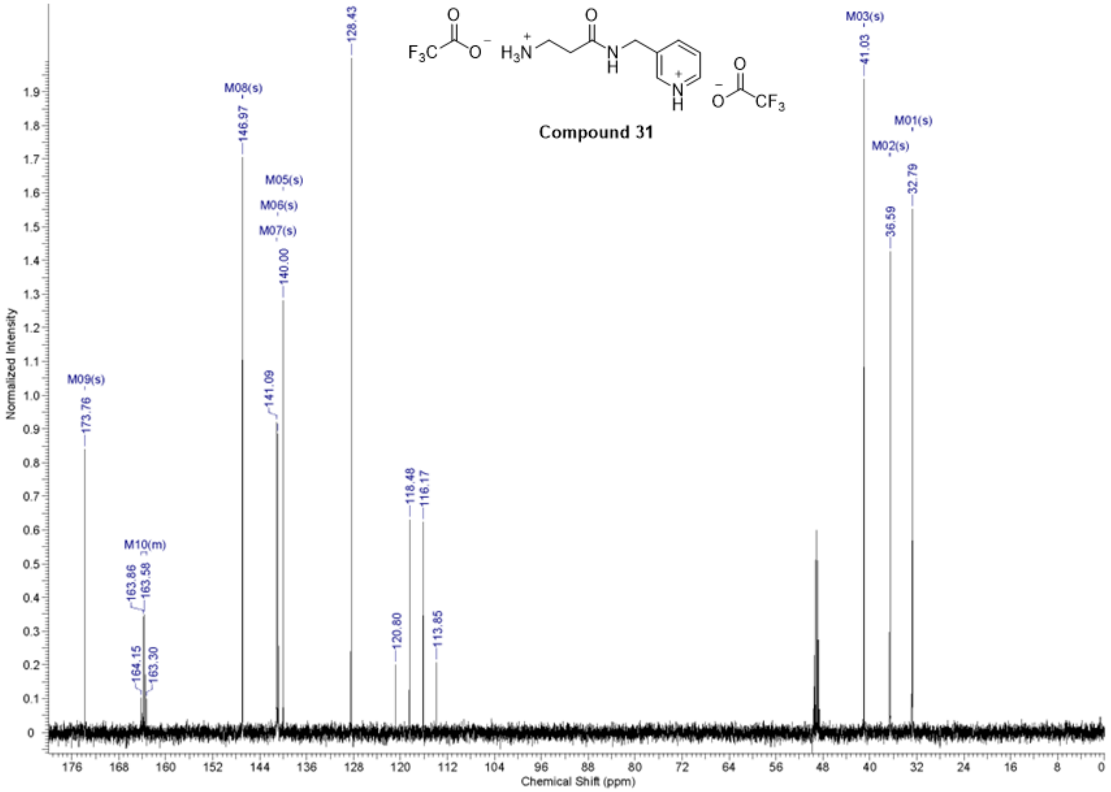

Compound 31
M01(s)
M02(s)
M03(s)
M05(s)
M06(s)
M07(s)
M08(s)
M09(s)
M10(m)
173.76
164.15
163.86
163.58
163.30
146.97
141.09
140.00
128.43
120.80
118.48
116.17
113.85
41.03
36.59
32.79
Normalized Intensity
Chemical Shift (ppm)

^{1}H NMR of **32**

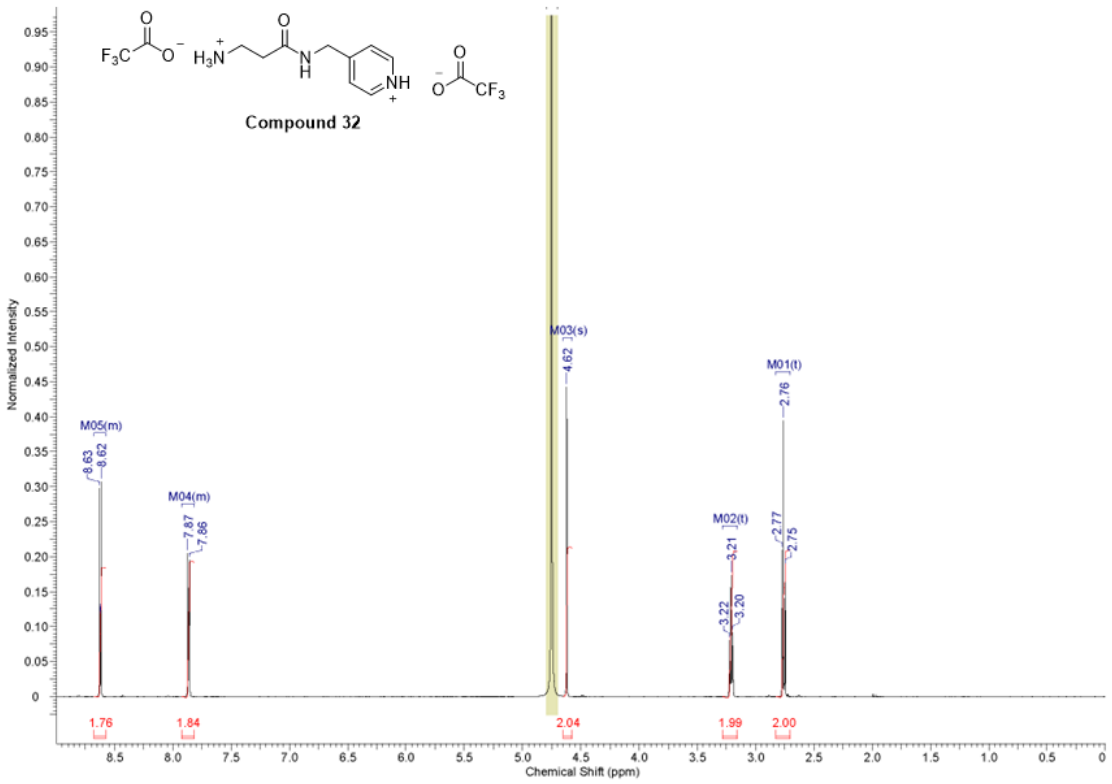

Compound 32
M05(m)
8.63
8.62
M04(m)
7.87
7.86
M03(s)
4.62
M02(t)
3.22
3.21
3.20
M01(t)
2.77
2.76
2.75
1.76
1.84
2.04
1.99
2.00
Normalized Intensity
Chemical Shift (ppm)

^{13}C NMR of **32**

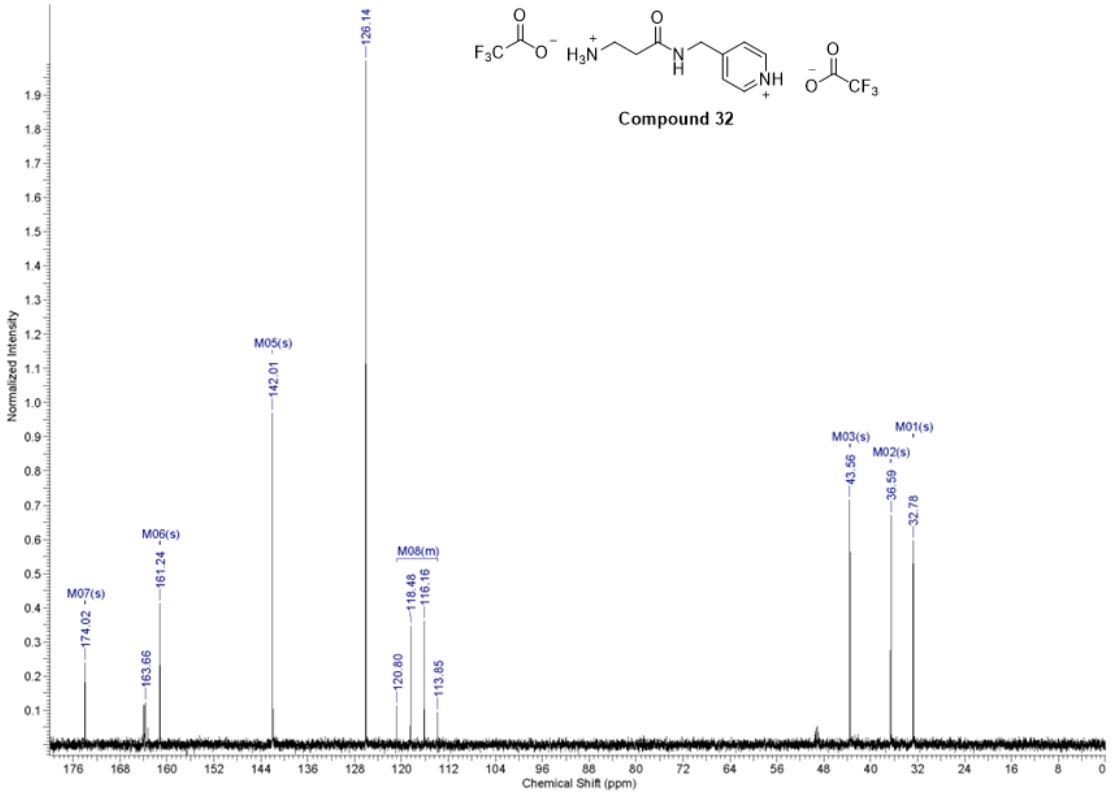
Compound 32
Normalized Intensity
Chemical Shift (ppm)
M07(s)
174.02
163.66
M06(s)
161.24
M05(s)
142.01
126.14
M08(m)
120.80
118.48
116.16
113.85
M03(s)
43.56
M02(s)
36.59
M01(s)
32.78

^{1}H NMR of **33**

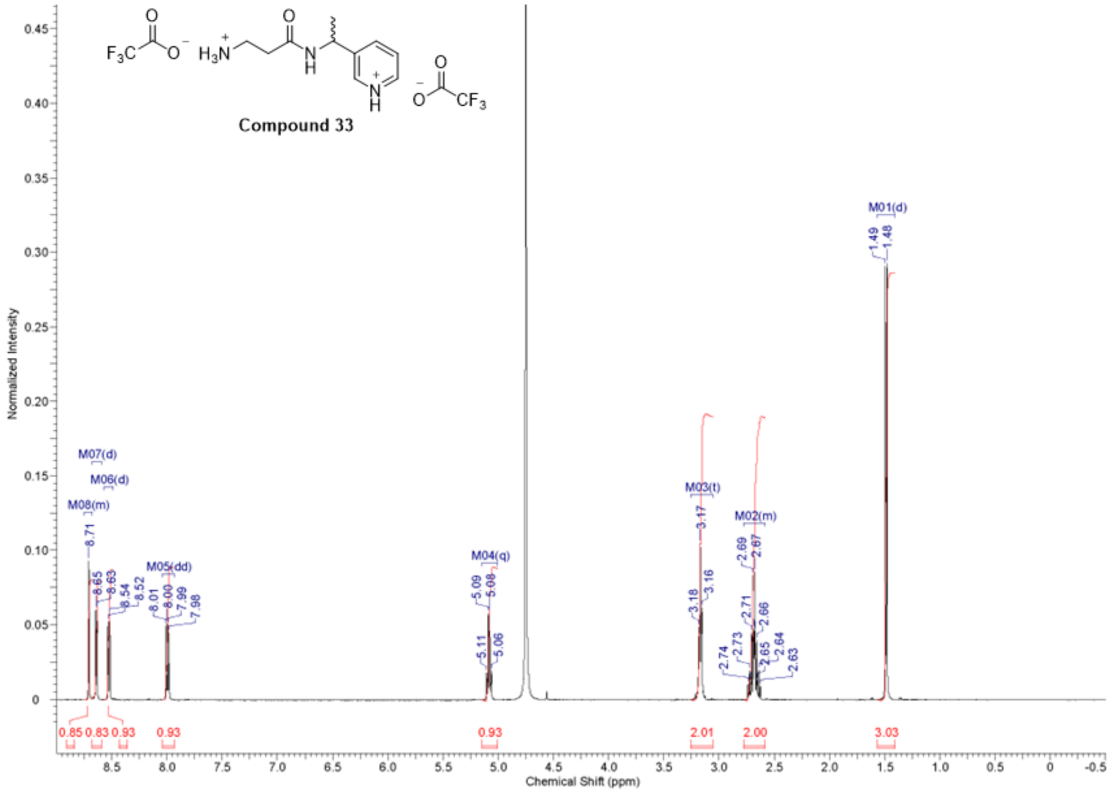

Compound 33
M01(d)
1.49
1.48
M02(m)
M03(t)
M04(q)
M05(dd)
M06(d)
M07(d)
M08(m)
0.85 0.83 0.93 0.93 0.93 2.01 2.00 3.03
Normalized Intensity
Chemical Shift (ppm)

^{13}C NMR of **33**

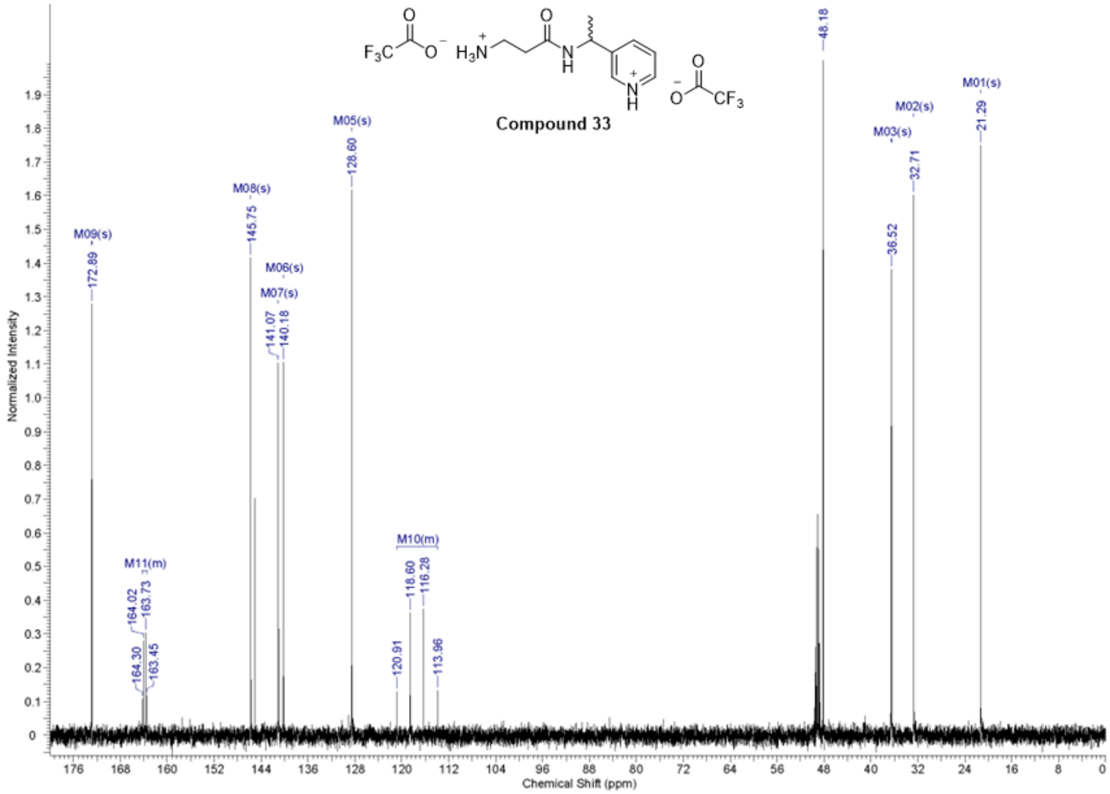

Compound 33
M09(s)
172.89
M11(m)
164.30
164.02
163.73
163.45
M08(s)
145.75
M06(s)
M07(s)
141.07
140.18
M05(s)
128.60
M10(m)
120.91
118.60
116.28
113.96
48.18
M03(s)
36.52
M02(s)
32.71
M01(s)
21.29
Normalized Intensity
Chemical Shift (ppm)

^{1}H NMR of **34**

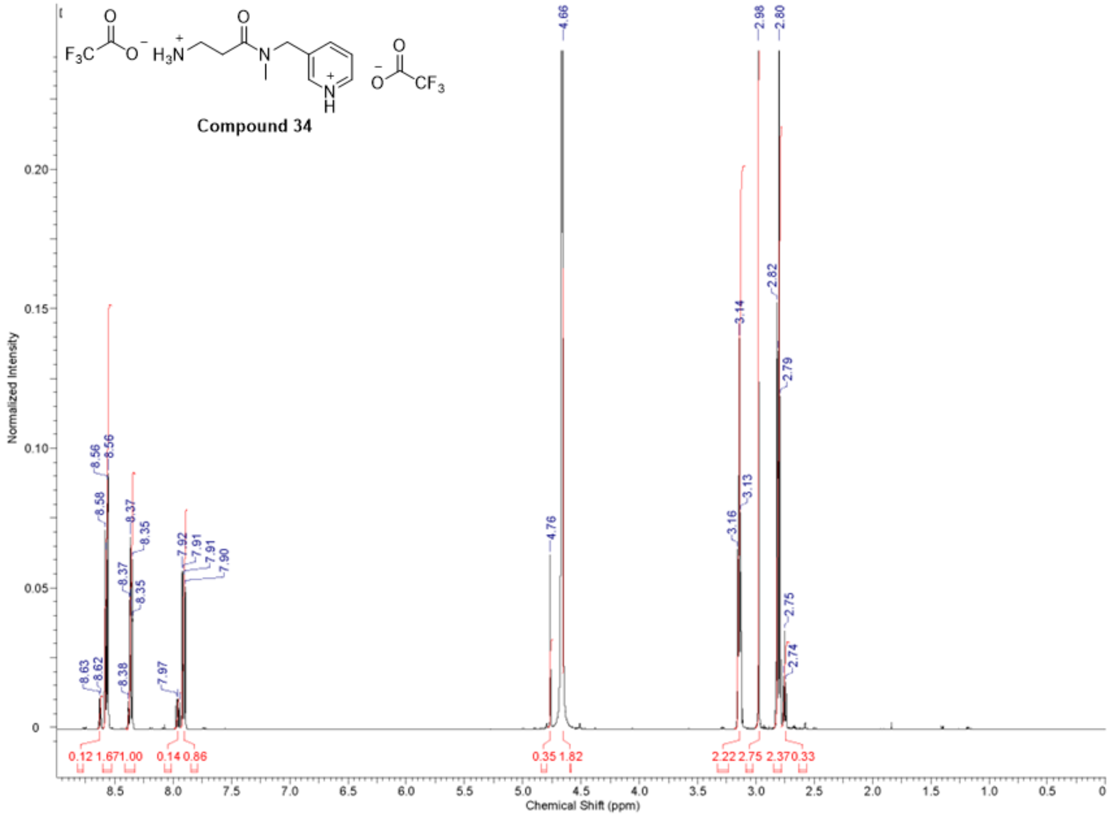

^{1}H NMR of **35**

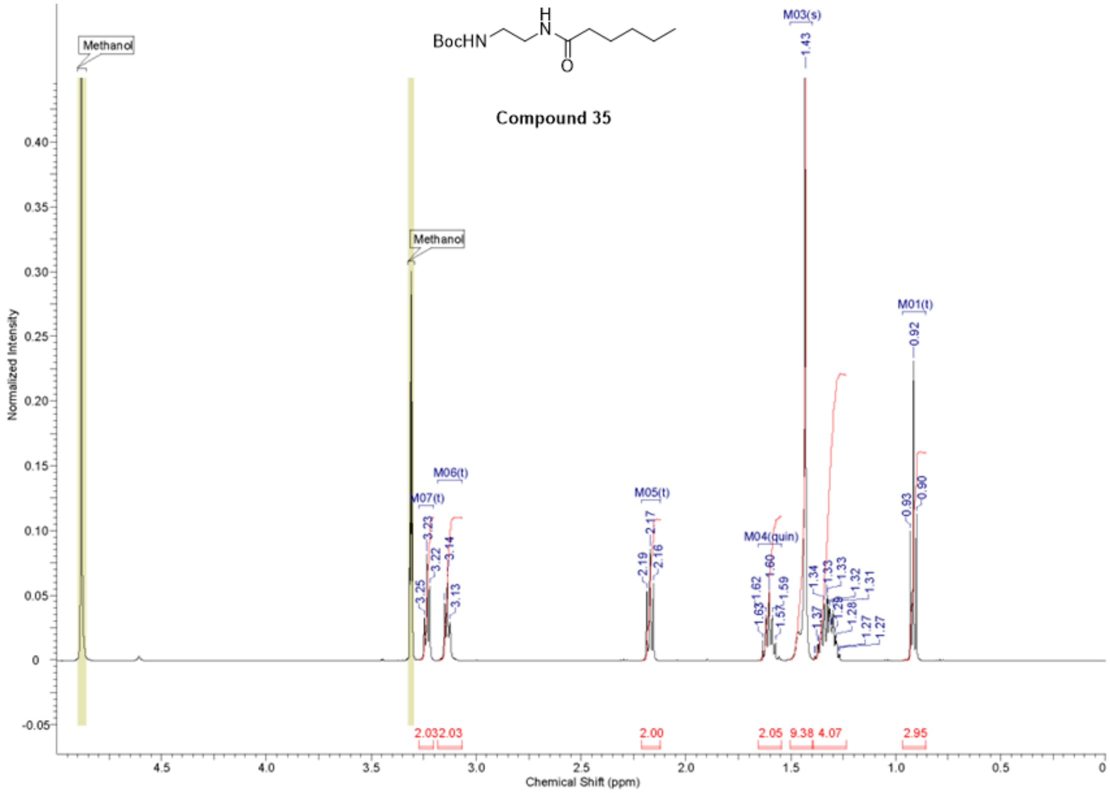

BocHN
H
N
O
Compound 35
Methanol
Methanol
M03(s)
M01(t)
M06(t)
M07(t)
M05(t)
M04(quin)
Normalized Intensity
Chemical Shift (ppm)
2.03
2.03
2.00
2.05
9.38
4.07
2.95

^{1}H NMR of **36**

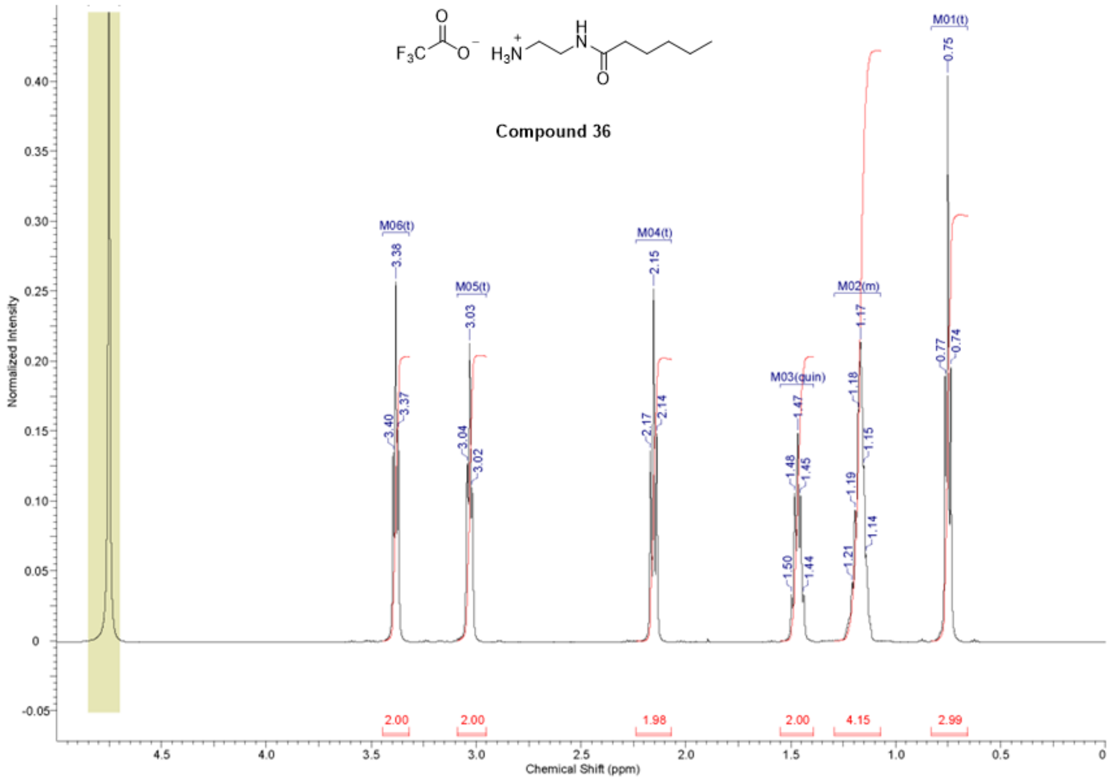
Compound 36
Normalized Intensity
Chemical Shift (ppm)
M06(t)
M05(t)
M04(t)
M03(quin)
M02(m)
M01(t)
2.00
2.00
1.98
2.00
4.15
2.99
